# Novel function of HATs and HDACs in homologous recombination through acetylation of human RAD52 at double-strand break sites

**DOI:** 10.1371/journal.pgen.1007277

**Published:** 2018-03-28

**Authors:** Takeshi Yasuda, Wataru Kagawa, Tomoo Ogi, Takamitsu A. Kato, Takehiro Suzuki, Naoshi Dohmae, Kazuya Takizawa, Yuka Nakazawa, Matthew D. Genet, Mika Saotome, Michio Hama, Teruaki Konishi, Nakako Izumi Nakajima, Masaharu Hazawa, Masanori Tomita, Manabu Koike, Katsuko Noshiro, Kenichi Tomiyama, Chizuka Obara, Takaya Gotoh, Ayako Ui, Akira Fujimori, Fumiaki Nakayama, Fumio Hanaoka, Kaoru Sugasawa, Ryuichi Okayasu, Penny A. Jeggo, Katsushi Tajima

**Affiliations:** 1 Research Center for Radiation Emergency Medicine, National Institute of Radiological Sciences (NIRS), Anagawa, Inage-ku, Chiba, Japan; 2 Program in Chemistry and Life Science, Department of Interdisciplinary Science and Engineering, School of Science and Engineering, Meisei University, Hodokubo, Hino-shi, Tokyo, Japan; 3 Department of Genetics, Research Institute of Environmental Medicine, Nagoya University, Furo-cho, Chikusa-ku, Nagoya, Japan; 4 Department of Environmental and Radiological Health Sciences, Colorado State University, Fort Collins, CO, United States of America; 5 Biomolecular Characterization Unit, RIKEN Center for Sustainable Resource Science, Hirosawa, Wako, Saitama, Japan; 6 Department of Genome Repair, Atomic Bomb Disease Institute, Nagasaki University, Sakamoto, Nagasaki, Japan; 7 Department of Basic Medical Sciences for Radiation Damage, NIRS, National Institutes for Quantum and Radiation Sciences and Technology (QST), Anagawa, Inage-ku, Chiba, Japan; 8 Research Center for Charged Particle Therapy, NIRS, Anagawa, Inage-ku, Chiba, Japan; 9 Cell-Bionomics Research Unit, Innovative Integrated Bio-Research Core, Institute for Frontier Science Initiative, Kanazawa University, Kakuma-machi, Kanazawa, Japan; 10 Radiation Safety Research Center, Nuclear Technology Research Laboratory, Central Research Institute of Electric Power Industry, Iwado Kita, Komae-shi, Tokyo, Japan; 11 Research Center for Radiation Protection, NIRS, 4-9-1 Anagawa, Inage-ku, Chiba, Japan; 12 Genome regulation and Molecular pharmacogenomics, School of Bioscience and Biotechnology, Tokyo University of Technology, Katakuramachi, Hachioji City, Tokyo, Japan; 13 International Open Laboratory (IOL), NIRS, Anagawa, Inage-ku, Chiba, Japan; 14 Faculty of Science, Gakushuin University, Mejiro, Toshima-ku, Tokyo, Japan; 15 Biosignal Research Center, and Graduate School of Science, Kobe University, Rokkodai-cho, Nada-ku, Kobe, Japan; 16 Genome Damage and Stability Centre, University of Sussex, Brighton, United Kingdom; Duke University, UNITED STATES

## Abstract

The p300 and CBP histone acetyltransferases are recruited to DNA double-strand break (DSB) sites where they induce histone acetylation, thereby influencing the chromatin structure and DNA repair process. Whether p300/CBP at DSB sites also acetylate non-histone proteins, and how their acetylation affects DSB repair, remain unknown. Here we show that p300/CBP acetylate RAD52, a human homologous recombination (HR) DNA repair protein, at DSB sites. Using *in vitro* acetylated RAD52, we identified 13 potential acetylation sites in RAD52 by a mass spectrometry analysis. An immunofluorescence microscopy analysis revealed that RAD52 acetylation at DSBs sites is counteracted by SIRT2- and SIRT3-mediated deacetylation, and that non-acetylated RAD52 initially accumulates at DSB sites, but dissociates prematurely from them. In the absence of RAD52 acetylation, RAD51, which plays a central role in HR, also dissociates prematurely from DSB sites, and hence HR is impaired. Furthermore, inhibition of ataxia telangiectasia mutated (ATM) protein by siRNA or inhibitor treatment demonstrated that the acetylation of RAD52 at DSB sites is dependent on the ATM protein kinase activity, through the formation of RAD52, p300/CBP, SIRT2, and SIRT3 foci at DSB sites. Our findings clarify the importance of RAD52 acetylation in HR and its underlying mechanism.

## Author summary

DNA double strand breaks (DSBs) are the most dangerous type of DNA damage in cells. Homologous recombination (HR) is a DSB repair system in which a central player, RAD51, functions with several proteins, including RAD52. DSBs activate the DNA damage response signaling network, in which the ataxia telangiectasia mutated (ATM) protein plays a chief role, by phosphorylating numerous target proteins. As compared to phosphorylated proteins, relatively few acetylated proteins have been functionally characterized in DNA repair. In addition, beyond the roles in phosphorylation signaling, much less is known about whether ATM functions are linked with other protein modifications, such as acetylation. Here, we found that RAD52 at DSB sites is acetylated by p300/CBP acetyltransferases and then deacetylated by SIRT2/SIRT3 deacetylases. RAD52 acetylation is required for sustained RAD51 colocalization at DSB sites, and is therefore essential in HR. ATM is required for the recruitment of RAD52, p300/CBP and SIRT2/SIRT3 to DSB sites, and therefore is essential for RAD52 acetylation. Thus, the RAD52-acetylation state is critical for HR, and its regulation is linked to ATM signaling. Our work demonstrates the importance of the regulation of RAD52 acetylation in HR and its underlying mechanism.

## Introduction

Ionizing radiation (IR) induces deleterious DNA lesions, such as DNA double-strand breaks (DSB). In response to DSBs, DNA damage response (DDR) signaling is induced. Ataxia telangiectasia mutated (ATM) protein kinase is one of the central players for phosphorylation-mediated DDR signaling, which is activated at DSB sites and phosphorylates numerous proteins, including the histone variant H2AX, and cell cycle checkpoint and DNA repair proteins [1]. Homologous recombination (HR) is an important mechanism for the repair of DSBs [2]. HR repairs DSBs through DNA strand invasion and exchange, in which the damaged DNA strand retrieves genetic information from an undamaged homologous DNA strand. After DSB formation, HR is initiated by a 5' to 3' end resection generating 3' single-stranded (ss) DNA overhangs. In mammalian cells, DSB end resection is mediated by the MRE11-RAD50-NBS1 (MRN)-CtIP complex and the EXO1 protein [3,4,5]. Afterwards, replication protein A (RPA) rapidly coats the 3'-overhang ssDNA regions, thereby removing secondary structures that form on the ssDNA region. Subsequently, the RPA coating the ssDNA regions is displaced by the RAD51 recombinase, to form a right-handed nucleoprotein filament. The RAD51 nucleoprotein filament then catalyzes DNA strand invasion and exchange between ssDNA and the homologous sequence within double-stranded (ds) DNA.

The replacement of RPA with RAD51 requires additional proteins, such as recombination mediators, because prior binding of RPA to ssDNA inhibits the nucleation of RAD51 on ssDNA. Biochemical studies using recombinant proteins demonstrated that the yeast Rad52 protein stimulates the Rad51-mediated displacement of RPA from ssDNA regions [6]. In the mouse, the targeted inactivation of *RAD52* reduces HR and may be involved in certain types of DSB repair processes [7]. However, this mediator function of human RAD52 for the loading of RAD51 onto the RPA-coated ssDNA region has never been demonstrated, despite extensive biochemical analyses [2,8]. Instead, biochemical studies have revealed that the human BRCA2 protein, which does not have a yeast homologue, promotes the RAD51 nucleoprotein filament formation on RPA-covered ssDNA *in vitro* [9,10]. Therefore, instead of RAD52, BRCA2 is thought to mediate RAD51-dependent HR in human cells. This is supported by the fact that a knockdown of BRCA2 in human cells decreases the efficiency of IR-induced RAD51 foci formation. Interestingly, a RAD52 knockdown in BRCA2-knockdown or BRCA2-deficient cells almost completely inhibits IR-induced RAD51 foci formation [11], which suggests that human RAD52 could act as a RAD51 mediator or complement the RAD51-dependent pathway in HR. Since most previous biochemical studies of RAD52 have utilized an unmodified, recombinant RAD52 protein expressed in *Escherichia coli*, it is possible that a recombination mediator activity of RAD52 might only be revealed upon post-translational modifications, as discussed by San Filippo et al. [2].

RAD52 preferentially binds ssDNA [12] rapidly and tightly, by wrapping the ssDNA around itself [13]. In contrast, RAD52 binds dsDNA slowly and weakly, but changes the dsDNA mechanics probably by intercalating into the DNA helix [13]. RAD52 also interacts with RPA and RAD51 [14,15]. Both yeast and human RAD52 exhibit ssDNA annealing activity [12,16], which may be required in the steps following strand invasion mediated by RAD51 [17,18], as well as in the RAD51-independent single-strand annealing (SSA) pathway [2]. Human RAD52 also has a D-loop formation activity [19]. Both human and yeast RAD52 are multimeric proteins [6,20]. Three-dimensional reconstitution from electron microscopy images revealed that full-length human RAD52 exists as a heptameric ring [21]. The crystal structure of the amino (N)-terminal half of RAD52 revealed an undecameric ring with a highly positively-charged groove outside the ring [22,23]. The N-terminal half of human RAD52 encompasses the catalytic domain for homologous pairing. Structure-based alanine scan mutagenesis of the N-terminal half of RAD52 revealed that several lysine (K) residues within the positively-charged groove are essential for DNA binding [22,24]. The carboxyl (C)-terminal region of human RAD52 contains domains that interact with RAD51 and RPA.

Post-translational modifications, such as phosphorylation, ubiquitylation, small ubiquitin-like modifier (SUMO)ylation, and acetylation, regulate biological processes by controlling a wide variety of protein functions. Previously, some post-translational modifications of Rad52 were identified. Yeast Rad52 is modified by SUMO at the K10, K11, and K220 sites, and the SUMOylation is induced by a treatment with DNA-damaging agents [25]. SUMOylation of yeast Rad52 protects it from proteasomal degradation. Human RAD52 is also modified by SUMO, but the SUMOylation sites of human RAD52 differ from those of yeast Rad52. The *in vitro* SUMOylation sites of human RAD52 are K411, K412, and K414, which are located within the putative nuclear localization signal near the C-terminus (Saito et al., 2010). SUMOylation does not affect the biochemical activities of human RAD52, but mutations at SUMOylation sites inhibit RAD52 nuclear localization [26]. Nuclear phosphatase and tensin homolog on chromosome 10 (PTEN) was recently found to be involved in regulating RAD52 SUMOylation [27]. PTEN is also modified by SUMOylation, which is involved in the exclusion of the protein from the nucleus [28]. The function of the SUMOylation of human RAD52, however, remains poorly understood. Human RAD52 is also phosphorylated at tyrosine (Y) 104 by c-ABL tyrosine kinase upon exposure to IR, and the phosphorylation deficiency inhibits the IR-induced foci formation of RAD52 [29]. Phosphorylation at Y104 enhances the ssDNA annealing activity of RAD52 by increasing the binding specificity for ssDNA [30]. No other post-translational modifications of RAD52 have yet been identified.

Among the several post-translational modifications, acetylation occurs on specific lysine residues and is catalyzed by histone acetyltransferases (HATs). Histones are well-known target proteins for acetylation. Histone acetylation influences chromatin structure, thereby regulating a wide variety of DNA transaction processes, such as transcription [31], DNA replication [32], DNA recombination [33], and DNA repair [34]. HATs can also acetylate non-histone proteins, including some DNA repair proteins [35,36]. During HR, CtIP is deacetylated by SIRT6 histone deacetylase (HDAC), and deacetylation is required for DNA end-resection, although the specific acetyltransferase for CtIP has not yet been identified [37]. The HATs, p300 and CBP, accumulate at laser microirradiation- or I-SceI-induced DSB sites, and promote histone acetylation at DSB sites [34]. Whether the accumulated p300 and CBP at DSB sites also induce the acetylation of non-histone proteins involved in DSB repair, however, is unclear.

Here, we provide evidence for human RAD52 acetylation by p300/CBP upon DSB induction, and its involvement in RAD51 localization at DSB sites during HR repair. We show how HR is regulated via RAD52 acetylation and reveal the link between the acetylation event and the ATM-dependent phosphorylation.

## Results

### Human RAD52 is acetylated by p300/CBP *in vitro*

**Fig 1:**
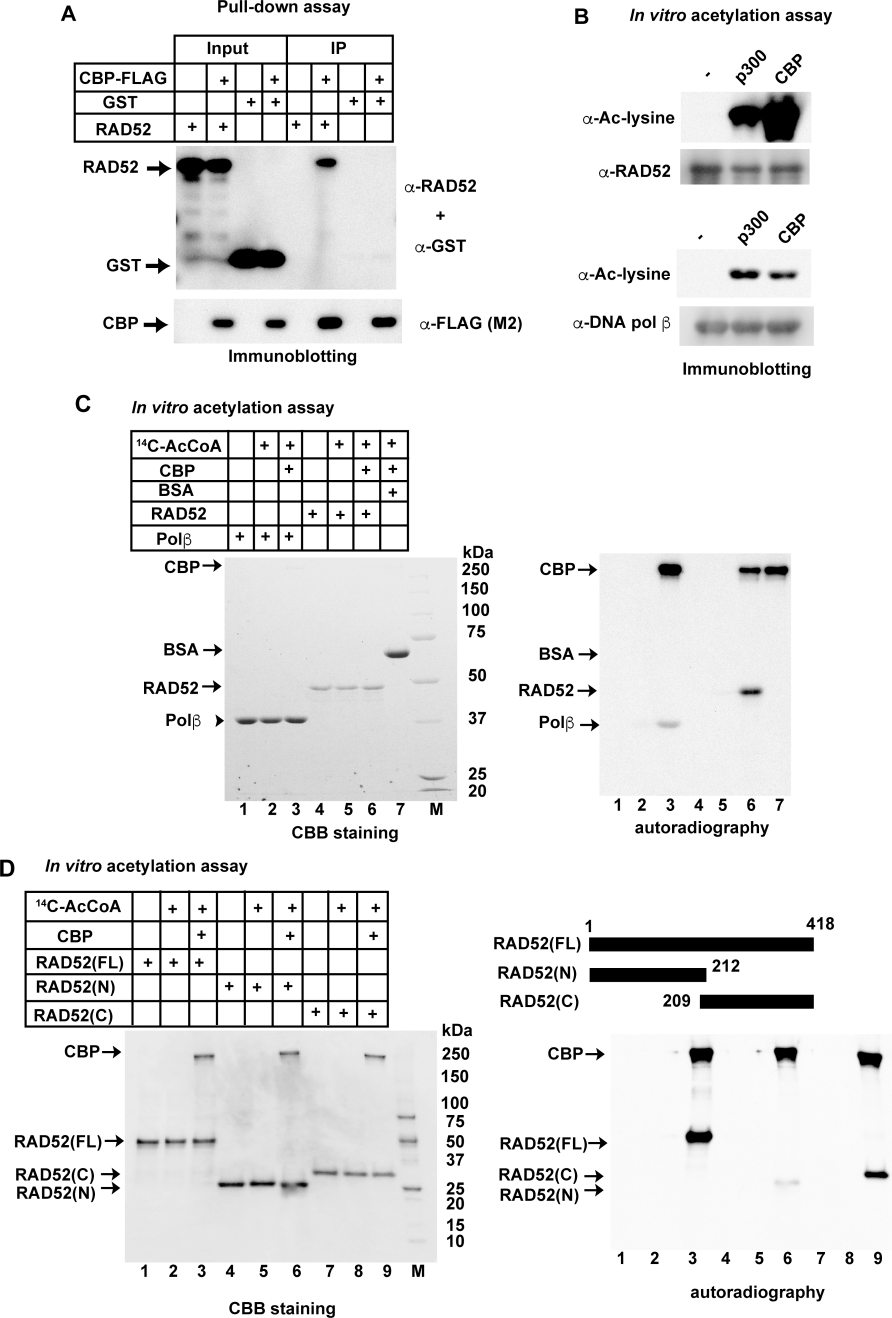
Human RAD52 is directly acetylated by p300/CBP *in vitro*. (A) Physical interaction of human RAD52 with CBP. The RAD52 or GST protein was incubated with or without CBP-FLAG in buffer P, and a pull-down assay was performed as described in the Supporting Materials and Methods. Input or immunoprecipitated (IP) proteins were detected by a mixture of anti-RAD52 and anti-GST antibodies (top) or an anti-FLAG (M2) antibody (bottom). (B) RAD52 (0.2 μg; top) or DNA polymerase β (0.2 μg; bottom) was incubated in 10 μl HAT buffer A containing 10 mM sodium butyrate and 0.4 μg acetyl coenzyme A (Ac-CoA) in the absence (-) or presence of HATs (42.5 ng of FLAG-p300 or 275 ng of CBP-FLAG) at 30°C for 90 min. Reaction mixtures were subjected to immunoblotting analyses. (C, D) *In vitro* acetylation assays were performed as described in the Supporting Materials and Methods, using HAT buffer A containing sodium butyrate. [^14^C]Ac-CoA was added where indicated. The reactions were analyzed by Coomassie Brilliant Blue staining (left) or autoradiography (right). Acetylated proteins can be detected by autoradiography. Bovine serum albumin (BSA), as a negative control of acetylation, was not detected in this assay. (C) RAD52 (3 μg), DNA polymerase β (3 μg), or BSA (3 μg) was incubated with CBP-FLAG (2 μg) where indicated. (D) RAD52 (FL, 2 μg), RAD52 (N, 2 μg), or RAD52 (C, 2 μg) was incubated with CBP-FLAG (1 μg), as indicated.

The acetylation of non-histone DNA repair proteins has attracted recent attention [35,36,37]. We searched for new HAT substrates among human DNA repair proteins, and found that human RAD52 interacted with CBP, one of the well-known HATs (Fig 1A). FLAG-tagged CBP coimmunoprecipitated with RAD52, but not with the glutathione S-transferase (GST) control. RAD52 also specifically interacted with p300, which is structurally and functionally similar to CBP (S1A Fig). An *in vitro* acetylation assay was performed to examine whether RAD52 is acetylated by either CBP or p300. DNA polymerase β was used as a positive acetylation control substrate [35]. Strikingly, the incubation of human RAD52 with p300 or CBP, in the presence of acetyl CoA, promoted RAD52 acetylation, which was detected by immunoblotting using an anti-acetyl lysine antibody (Fig 1B). RAD52 acetylation was also confirmed by an *in vitro* acetylation assay using ^14^C-labeled acetyl CoA; the ^14^C-labeled acetyl group was transferred onto the ε-amino group of the lysine residue. Acetylation of RAD52 was specifically detected when RAD52 was incubated with CBP (Fig 1C; lane 6) or p300 (S1B Fig; lane 6) in the presence of ^14^C-labeled acetyl CoA. Notably, RAD52 was more efficiently acetylated than the control substrate, DNA polymerase β (lane 3 in Fig 1C and S1B Fig). By contrast, neither RAD51 nor DNA polymerase κ [38], which are key factors in homologous recombination, was acetylated by CBP or p300 *in vitro* (S1C–S1E Fig).

### Identification of acetylated sites of RAD52

**Fig 2:**
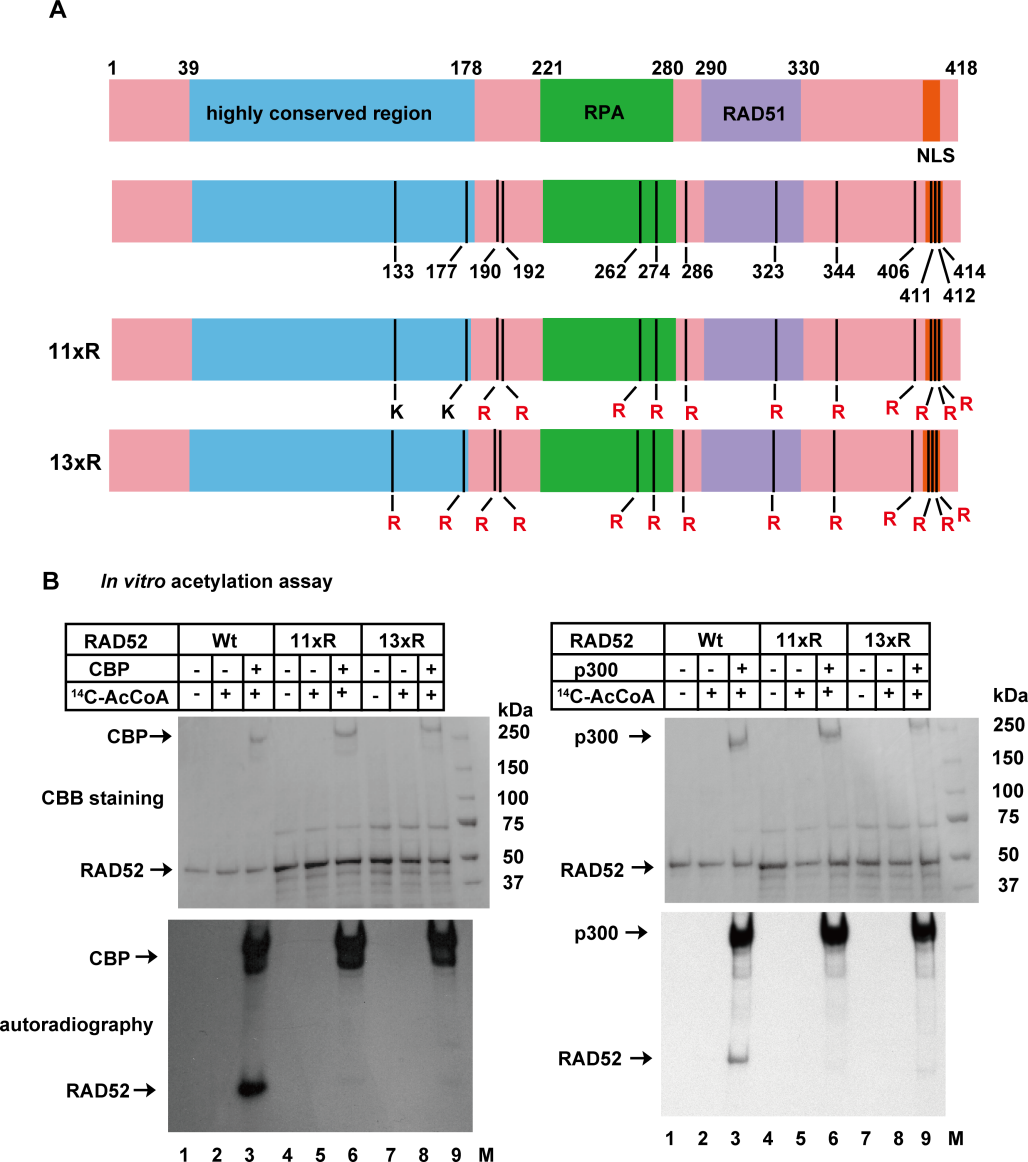
Identification of human RAD52 acetylation sites. (A) Location of RAD52 acetylation sites identified by LC-MS analysis. Acetylated amino acid residues, highly conserved region responsible for DNA binding and self-association, RPA binding domain (RPA), RAD51 binding domain (RAD51), and NLS are indicated in the schematic representation. The RAD52 (11xR) and RAD52 (13xR) mutant proteins used in Fig 2B are shown as schematic representations. (B) *In vitro* acetylation assays were performed as described in the Supporting Materials and Methods, using HAT buffer A containing sodium butyrate. RAD52 (Wt, 2 μg), RAD52 (11xR, 2 μg), or RAD52 (13xR, 2 μg) was incubated with CBP-FLAG (500 ng; left), FLAG-p300 (500 ng; right), and [^14^C] Ac-CoA where indicated. The reactions were analyzed by Coomassie Brilliant Blue staining (upper) or autoradiography (bottom).

To map the acetylated sites in RAD52, we performed an *in vitro* acetylation assay with the N- or C-terminal half of RAD52 (Fig 1D and S1F Fig). Both RAD52 fragments were acetylated by CBP or p300, although the C-terminal fragment of RAD52 (209–418) was more efficiently acetylated. To identify the acetylated residues, we performed a liquid chromatography mass spectrometry (LC-MS) analysis using *in vitro* acetylated full-length, N-terminal, and C-terminal RAD52 fragments. We identified 11 acetylation sites in RAD52 (FL), and two additional acetylation sites (K133, K177) in the C-terminally truncated RAD52 (N) (S1–S4 Tables and Fig 2A). We next purified two full-length RAD52 mutants, one containing arginine substitutions at the 11 acetylation sites (11xR), and the other containing arginine substitutions at all 13 identified acetylation sites (13xR). Using 11xR and 13xR, we confirmed that the p300/CBP-mediated acetylation of RAD52 was diminished when 11 or all 13 of the identified lysine residues were mutated to arginine (Fig 2B). The identified sites are well conserved among different species (S2 Fig). Lysines 411, 412, and 414 were previously identified as SUMOylation sites, and overlap with the nuclear localization signal (NLS) [26]. Mutations at these SUMOylation sites inhibited RAD52 nuclear localization. Another notable acetylation site is lysine 133, which is an important site for DNA binding [24]. To study the RAD52 acetylation in detail, we used the acetylation-deficient, lysine-to-arginine substituted mutants in *in vivo* studies (S3 Fig). The 11xR mutant and the unmodified RAD52 (Wt) displayed similar ssDNA binding activities, suggesting that the multiple lysine to arginine substitutions do not affect the RAD52 activity (S4A and S4B Fig).

### Human RAD52 is acetylated *in vivo*

To examine whether RAD52 is acetylated in human cells, we expressed an N-terminally FLAG-tagged RAD52 in human embryonic kidney 293 (HEK293) cells, and immunoprecipitated RAD52 using anti-FLAG antibody-conjugated agarose. Overexpression of CBP induced RAD52 acetylation, based on immunoblotting using an anti-acetyl lysine antibody (S5A Fig).

**Fig 3:**
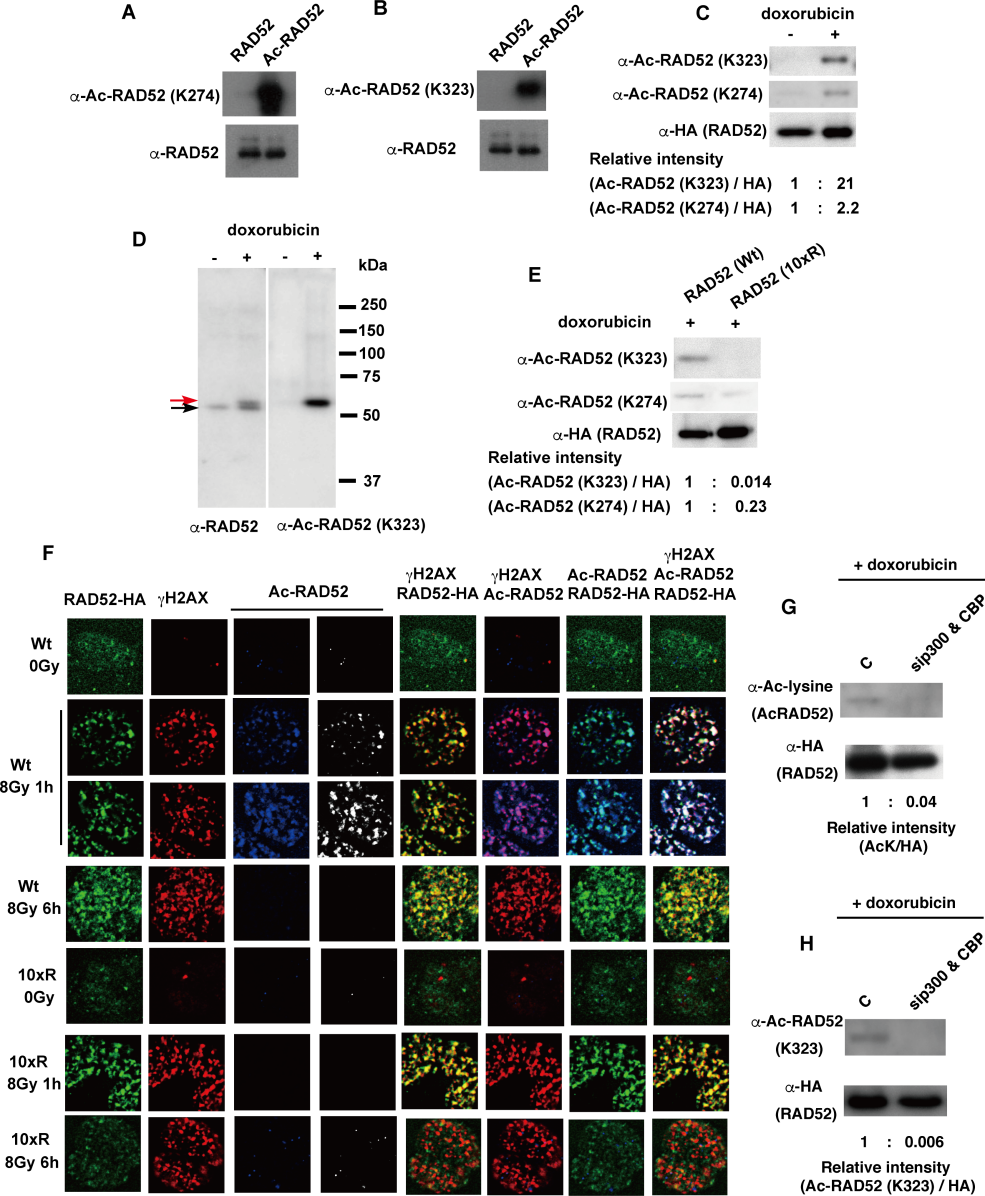
Human RAD52 is acetylated *in vivo*. (A, B) An *in vitro* acetylation assay was performed by incubating RAD52 (0.25 μg) in 10 μl HAT buffer A containing 10 mM sodium butyrate, in the presence of 0.5 μg Ac-CoA and CBP-FLAG (54 ng), at 30°C for 60 min. Unacetylated and acetylated RAD52 were subjected to immunoblotting analyses using the indicated antibodies. (C, D, E) Acetylation of the FLAG-RAD52-HA protein purified from MSCs was detected as described in the Supporting Materials and Methods, using the indicated antibodies. The expression plasmid, pT-Rex-DEST30 containing FLAG-RAD52 (Wt)-HA or FLAG-RAD52 (10xR)-HA, was transfected into the cells. FLAG-RAD52-HA was purified from cell extracts 24 h after transfection. Cells were treated with doxorubicin for 2 h, as indicated. (D) The red arrow indicates the mobility-shifted band. (F) MSCs expressing FLAG-RAD52 (Wt)-HA or FLAG-RAD52 (10xR)-HA were unirradiated or irradiated with γ-rays (8 Gy). At the indicated time after irradiation, the cells were subjected to immunofluorescent staining with anti-HA (green), anti-γH2AX (red), and anti-acetylated RAD52 at K323 (blue or white) antibodies. (G, H) T-Rex-293 cells expressing FLAG-RAD52-HA were transfected with either a negative control siRNA or mixture of p300 and CBP-specific siRNAs. At 24 h after transfection, the cells were treated with doxorubicin for 2 h. Immunoprecipitated FLAG-RAD52-HA proteins from the cell extracts were subjected to immunoblotting analyses with the indicated antibodies. (C, E, G, H) The relative band intensities normalized to those of the HA bands are shown below the immunoblots.

To evaluate the acetylation status and localization of RAD52 *in vivo*, we produced anti-acetyl RAD52 antibodies against the acetylated lysine residues 274 or 323. The antibody specificity for acetylated RAD52 was confirmed by comparison with the *in vitro* acetylated or non-acetylated RAD52 protein (Fig 3A and 3B). Immunoblotting with each antibody revealed a positive reaction to the acetylated RAD52 protein (Fig 3A and 3B). We then used these anti-acetyl RAD52 antibodies to examine whether the induction of DSBs changes the acetylation status of RAD52 in cells. We used the chemical DSB inducer doxorubicin, because it constitutively produces DSBs in the cells, and thus we thought it may induce a stronger DSB signal. Doxorubicin induced RAD52 acetylation in repair-proficient mesenchymal stem cells (MSCs; Fig 3C) [39]. A band shift of *in vivo* acetylated RAD52 was observed following doxorubicin treatment (Fig 3D). The migration distance of the *in vitro* acetylated RAD52 was indistinguishable from that of the non-acetylated RAD52 in sodium dodecyl sulfate-polyacrylamide gel electrophoresis (SDS-PAGE) (Fig 1B). This was not the case for the acetylated RAD52 produced *in vivo* (Fig 3A), raising the possibility that the *in vivo* acetylation induces additional modifications of RAD52. Furthermore, we expressed a RAD52 construct in which 10 of the acetylation sites, except for lysine 411, 412, and 414 involved in the nuclear localization of RAD52 [26], were substituted with arginine (10xR; Fig 3E and S3 Fig), and performed immunoblotting analyses to examine whether the mutations affect the interaction between RAD52 and the anti-acetyl RAD52 antibody. We did not achieve a positive signal with the anti-acetyl RAD52 antibody (K323) in the presence of the 10xR mutant (Fig 3E) or in the absence of RAD52-HA expression by the vector (S6 Fig). These findings indicate that the anti-acetyl RAD52 antibody (K323) specifically detects and precisely evaluates the cellular acetylation status of RAD52. The anti-acetyl RAD52 antibody (K274) also specifically detected the acetylation of RAD52, but the specificity of the anti-acetyl RAD52 antibody (K323) against acetylated RAD52 was superior to that of the anti-acetyl RAD52 antibody (K274) (Fig 3C and 3E).

To examine the RAD52 acetylation at DSB sites, MSCs expressing RAD52 (Wt) or the RAD52 (10xR) mutant were irradiated with 8 Gy γ-rays and examined by an immunofluorescence approach. IR treatment transiently induces DSBs in cells, and thus IR is better suited for examining the level of RAD52 acetylation as a function of time. RAD52 foci, which colocalize with phosphorylated H2AX (γH2AX), were detected with both wild-type and mutant RAD52 (Fig 3F). In contrast, radiation-induced acetylated RAD52 (assessed using the anti-acetyl RAD52 antibody) was only detected in cells expressing RAD52 (Wt) protein at 1 h after IR. Acetylation of RAD52 (Wt) was not detected at 6 h after IR, suggesting that deacetylation of RAD52 occurs between 1–6 h at DSB sites. Significant acetylated RAD52 signals were not detected in cells expressing the RAD52 (10xR) protein throughout the time period studied, indicating that the anti-acetyl RAD52 antibody specifically recognized acetylated RAD52 by immunofluorescence. We also investigated the distribution of p300 and CBP in these irradiated cells, and observed p300 and CBP foci, which colocalized with γH2AX, at 1 h and 6 h after IR in human fetal lung fibroblast (MRC5) cells (S5B and S5C Fig). This finding is consistent with a previous report that describes the recruitment of p300/CBP to laser-induced DSB sites [34]. The p300 and CBP foci also colocalized with the RAD52 foci, as observed at 1 h or 6 h after IR in HEK293 cells (S5D and S5E Fig). In addition, the specific interaction of p300 or CBP with RAD52, but not GST, was detected by dithiobis succinimidyl propionate (DSP)-mediated *in vivo* cross-linking experiments in exogenously p300- or CBP-overexpressing HEK293 cells (S5F and S5G Fig). The interaction of p300 with RAD52 was increased upon doxorubicin treatment (S5F Fig). Finally, the knockdown of both p300 and CBP inhibited the DSB-induced acetylation of RAD52 (Fig 3G and 3H; S5H and S5I Fig). Taken together, our findings indicate that IR induces p300/CBP foci formation and acetylation of RAD52 at DSB sites.

### RAD52 acetylation is inhibited by its binding to DNA and RPA

**Fig 4:**
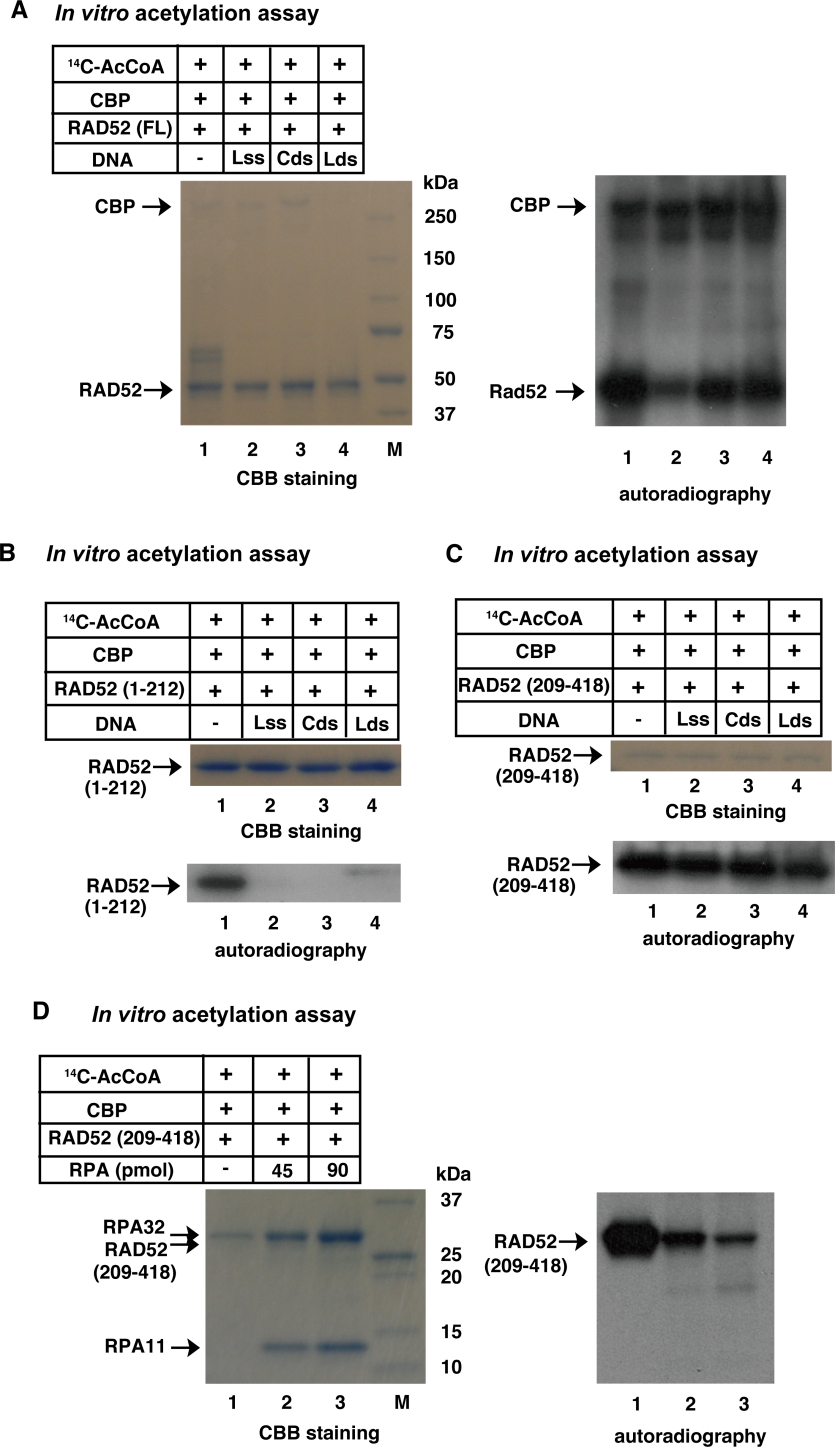
*In vitro* acetylation of RAD52 is inhibited in the presence of DNA or RPA. *In vitro* acetylation assays were performed as described in the Supporting Materials and Methods, using HAT buffer A containing sodium butyrate. The full-length (A), N-terminal half (B), or C-terminal half (C, D) of RAD52 (2 μg) was incubated with [^14^C] Ac-CoA and CBP-FLAG (500 ng). (A, B, C) RAD52 was premixed with 8,500 pmol (in nucleotides) of linear ssDNA, circular dsDNA, or linear dsDNA before the addition of CBP and Ac-CoA to the reaction mixture. (D) RAD52 was premixed with the indicated amount of RPA, before adding CBP and Ac-CoA to the reaction mixture.

By analogy with yeast Rad52, human RAD52 is reported to bind to RPA-coated ssDNA followig resection. RAD52 is also thought to bind to both ssDNA and dsDNA during the DNA strand exchange reaction. The N-terminal half of RAD52 contains a DNA binding region, whereas the C-terminal half contains an RPA binding region. Therefore, using an *in vitro* acetylation assay, we examined whether RAD52 binding to DNA or RPA affects the level of its acetylation. The addition of linear (L) ssDNA to the reaction mixture for the *in vitro* acetylation of RAD52 (FL) decreased the level of RAD52 acetylation (Fig 4A). The addition of circular (C) or linear dsDNA also decreased the acetylation of RAD52 (FL), but less than that of the linear ssDNA. Auto-acetylation of CBP was not affected by the addition of these DNA substrates. The N-terminal half of RAD52, RAD52 (1–212), contains the DNA-binding domain (Fig 2A). The acetylation of RAD52 (1–212) was completely inhibited by the addition of any one of the DNA substrates (Fig 4B), whereas the acetylation of the C-terminal half of RAD52, RAD52 (209–418), was not affected by the addition of the DNA substrates (Fig 4C). RAD52 (209–418) contains the RPA-binding domain (Fig 2A), and RAD52 (209–418) acetylation was inhibited by the addition of RPA in a dose-dependent manner (Fig 4D). Therefore, these results suggest that at least some of the acetylation sites of RAD52 are located at the DNA and RPA interacting surfaces.

### SIRT2 and SIRT3 are involved in the deacetylation of acetylated RAD52 at DSB sites

**Fig 5:**
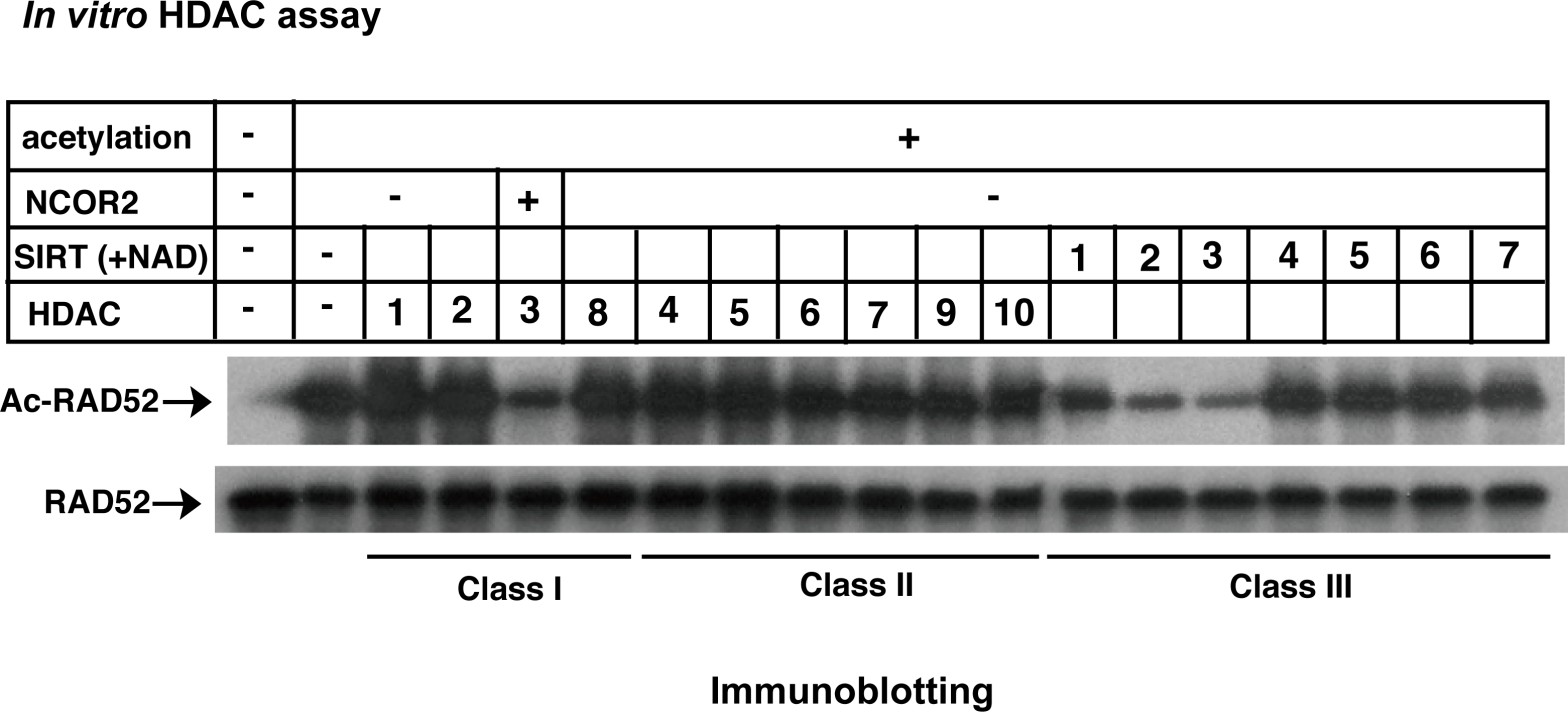
Screening of HDACs for RAD52 *in vitro*. *In vitro* HDAC assays were performed as described in the Supporting Materials and Methods, using acetylated RAD52 and recombinant HDAC proteins. Reaction mixtures were analyzed by immunoblotting with an anti-acetyl lysine antibody (top) or an anti-RAD52 (bottom) antibody.

HATs and HDACs regulate protein acetylation levels. Therefore, we next examined which HDACs are involved in the deacetylation of acetylated RAD52. After RAD52 acetylation by CBP *in vitro*, linear ssDNA was added to the reaction mixture to inhibit further RAD52 acetylation. The reaction mixture was divided, and each sample was subjected to an *in vitro* deacetylation assay using recombinant HDAC proteins. Among the HDAC proteins examined, the recombinant HDAC3/NCOR2 complex, SIRT2, and SIRT3 proteins deacetylated the acetylated RAD52 (Fig 5).

**Fig 6:**
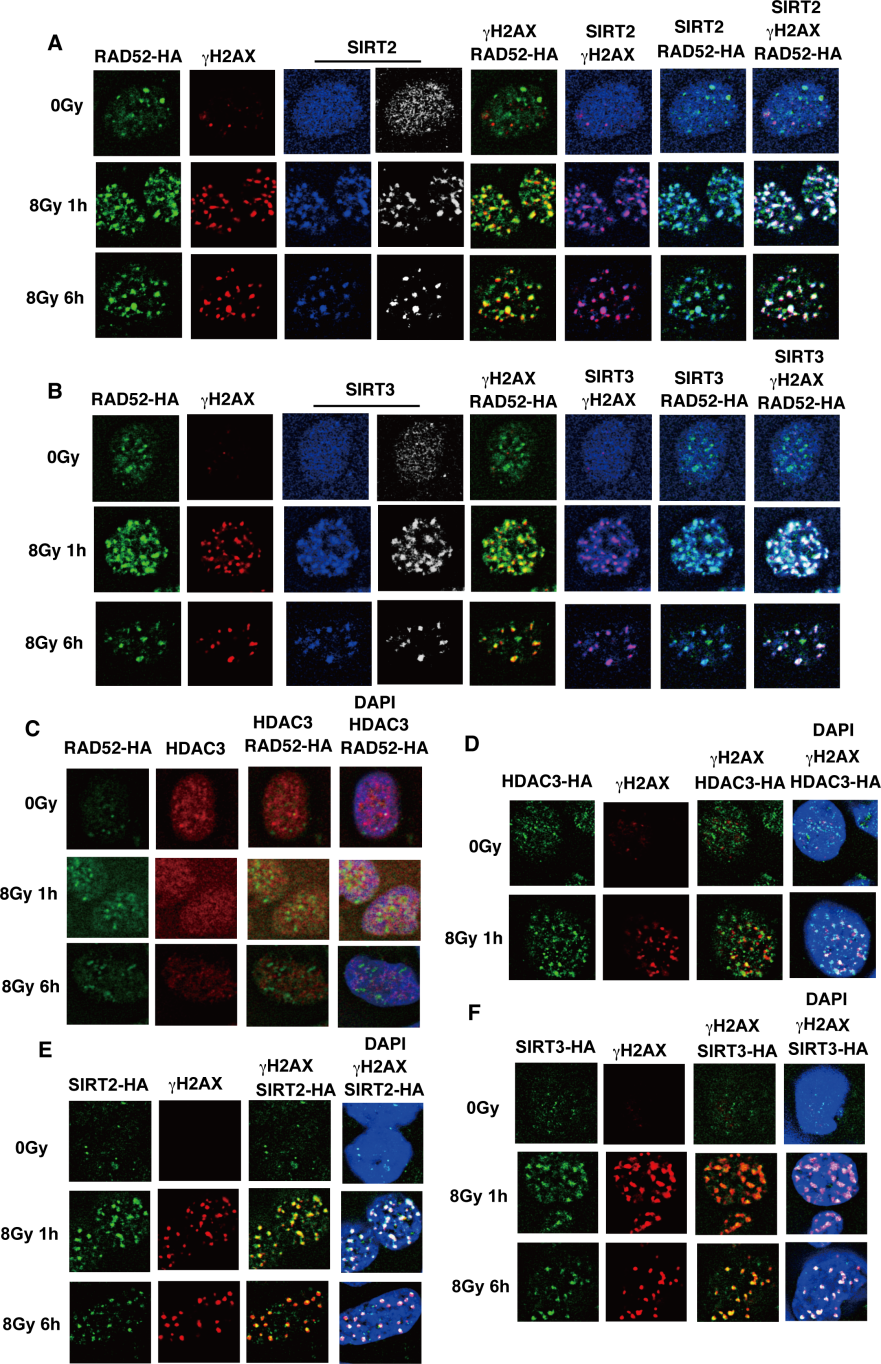
Colocalization of SIRT2 and SIIR3 with RAD52 at IR-induced DSB sites. (A, B, C) T-Rex-293 cells expressing FLAG-RAD52 (Wt)-HA were unirradiated or irradiated with γ-rays (8 Gy), and subjected to immunofluorescent staining at the indicated time after IR, using (A) anti-HA (green), anti-γH2AX (red), and anti-SIRT2 (blue or white) antibodies. (B) Anti-HA (green), anti-γH2AX (red), and anti-SIRT3 (blue or white) antibodies were used. (C) An anti-HA (green) antibody, an anti-HDAC3 (red) antibody, and DAPI (blue) were used. (D, E, F) T-Rex-293 cells expressing HDAC3-HA (D), SIRT2-HA (E) or SIRT3-HA (F) were unirradiated or irradiated with γ-rays (8 Gy). At the indicated time after irradiation, the cells were subjected to immunofluorescent staining with an anti-HA (green) antibody, an anti-γH2AX (red) antibody, and DAPI (blue).

**Fig 7:**
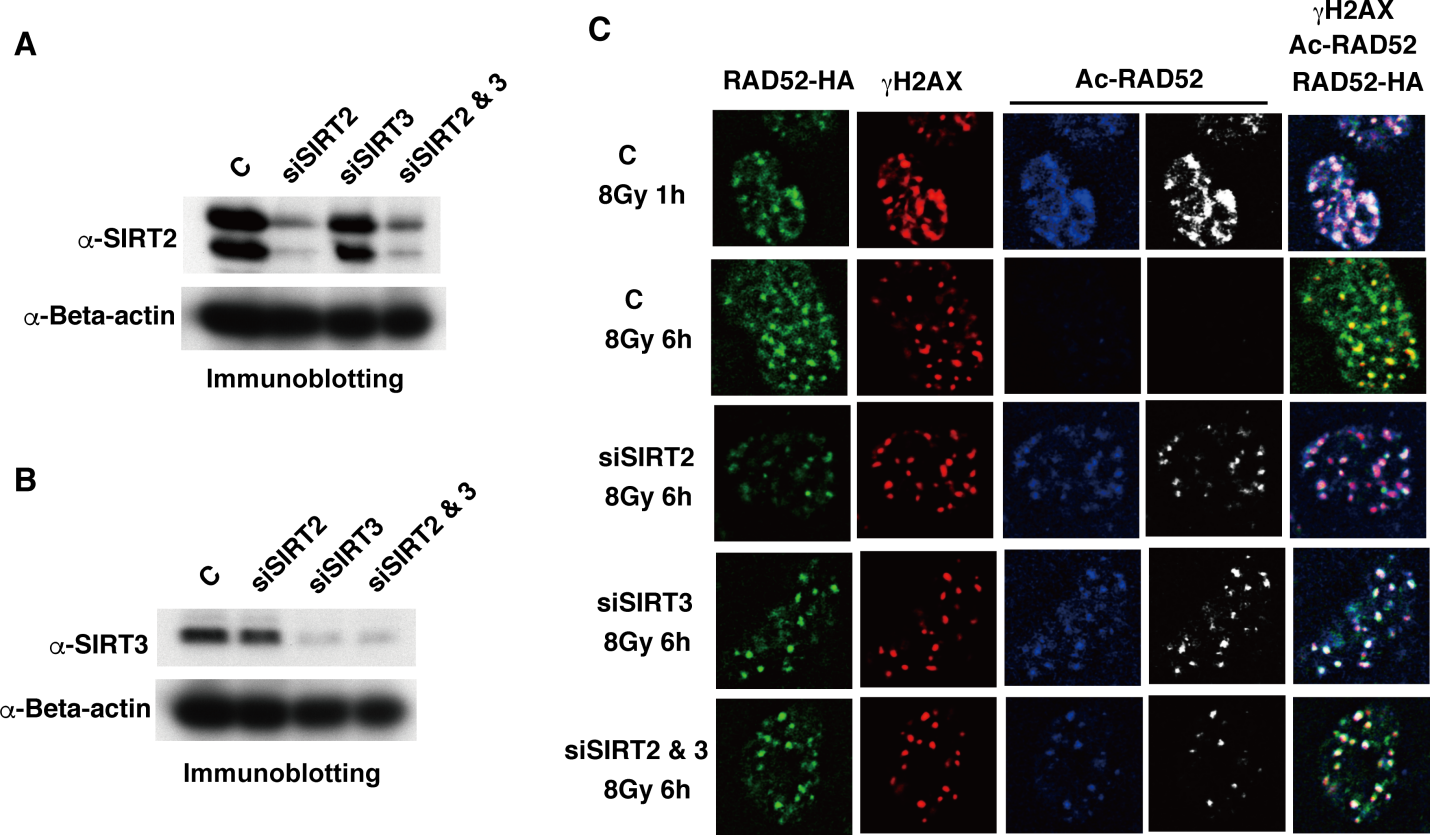
SIRT2 and SIRT3 are involved in the deacetylation of RAD52 at DSB sites. T-Rex-293 cells expressing FLAG-RAD52 (Wt)-HA were treated with either control siRNA (C), SIRT2-specific siRNA (siSIRT2), SIRT3-specific siRNA (siSIRT3), or both siRNAs (siSIRT2 & 3), as described in the Supporting Materials and Methods. (A, B) Cell extracts were prepared 48 h after siRNA transfection, and were subjected to immunoblotting analyses with the indicated antibodies. (C) Cells were irradiated with γ-rays (8 Gy) 48 h after siRNA transfection. At 1 h or 6 h after irradiation, cells were subjected to immunofluorescent staining with anti-HA (green), anti-γH2AX (red), and anti-acetylated RAD52 at K323 (blue or white) antibodies.

Since the acetylated RAD52 appeared to be deacetylated at DSB sites after IR (Fig 3F), we examined whether the identified HDACs for RAD52 were recruited to DSB sites after IR. We observed that SIRT2 and SIRT3, but not HDAC3, colocalized with γH2AX and/or RAD52 at 1 h after IR (Fig 6A–6F). The SIRT2 and SIRT3 foci colocalized with γH2AX and RAD52 even at 6 h after IR (Fig 6A, 6B, 6E and 6F), suggesting that SIRT2 and/or SIRT3 are involved in the deacetylation of acetylated RAD52 at DSB sites. Therefore, we examined whether the knockdown of *Sirt2* or *Sirt3* affects the decrease of RAD52 acetylation observed at 6 h after IR. The expression of SIRT2 and SIRT3 was effectively reduced by a small interfering (si)RNA treatment (Fig 7A and 7B, S7A and S7B Fig). Importantly, acetylated RAD52 was maintained at DSB sites even at 6 h after IR following SIRT2 or SIRT3 depletion (Fig 7C). Therefore, our results strongly suggest that SIRT2 and SIRT3 deacetylate RAD52 at DSB sites.

### RAD52 acetylation is required for efficient accumulation of RAD52 at DSB sites

**Fig 8:**
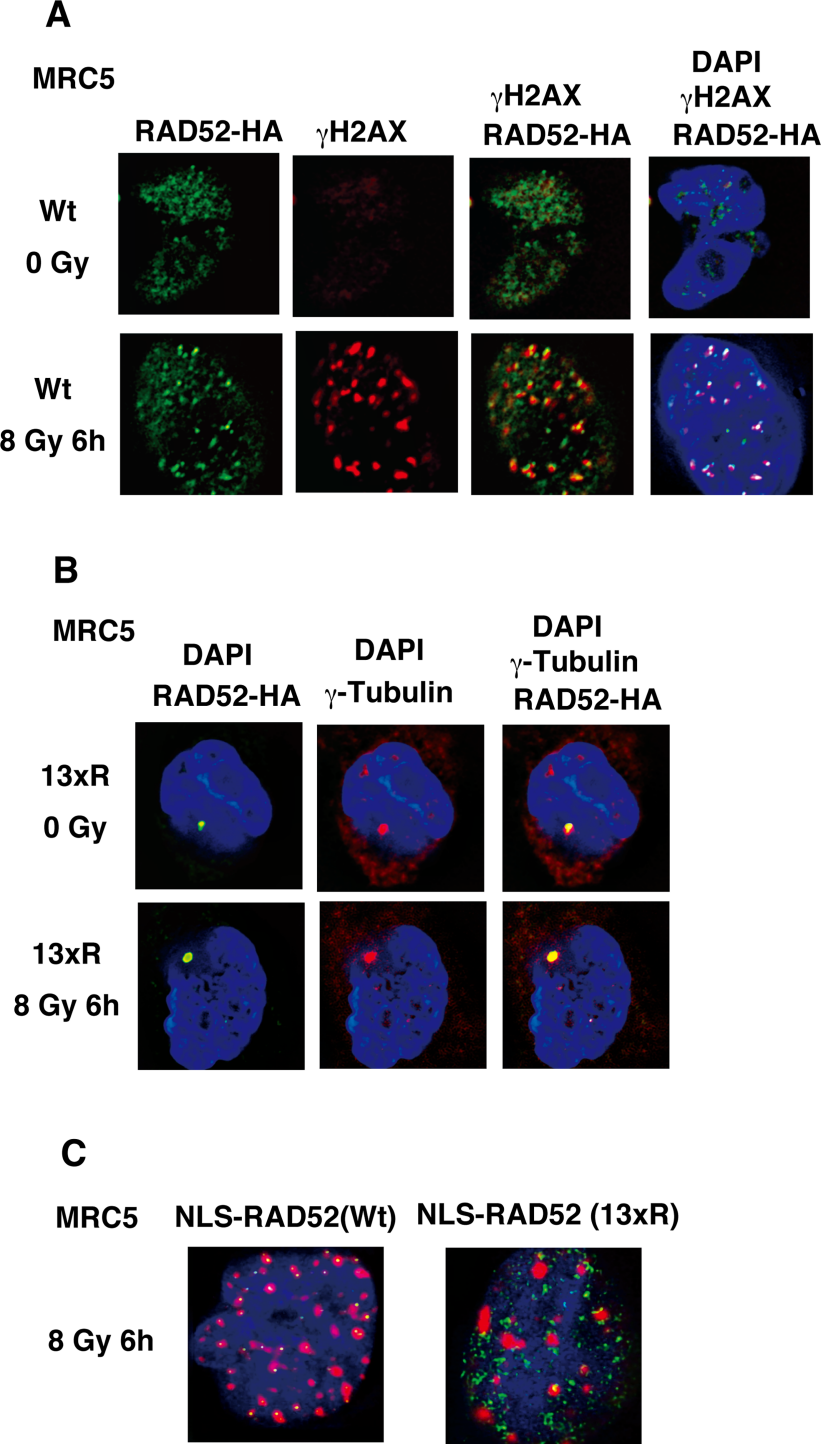
Effect of 13xR mutation on ionizing radiation-induced foci formation by RAD52. (A, B) MRC5V1-TR cells were unirradiated or irradiated with γ-rays (8 Gy), and subjected to immunofluorescent staining at the indicated time after irradiation. (A) Cells expressing FLAG-RAD52 (Wt)-HA were used. An anti-HA (green) antibody, an anti-γH2AX (red) antibody, and 4',6-diamidino-2-phenylindole (DAPI, blue) were used for immunofluorescent staining. (B) Cells expressing FLAG-RAD52 (13xR)-HA were used. An anti-HA (green) antibody, an anti-γ-tubulin (red) antibody, and DAPI (blue) were used for immunofluorescent staining. (C) MRC5V1-TR cells expressing FLAG-NLS-RAD52 (Wt)-HA or FLAG-NLS-RAD52 (13xR)-HA were irradiated with γ-rays (8 Gy), and subjected to immunofluorescent staining 6 h after irradiation. An anti-HA (green) antibody, an anti-γH2AX (red) antibody, and DAPI (blue) were used for immunofluorescent staining, and the overlaid images are shown.

The RAD52 protein localized to DSB sites after IR (Figs 3F and 8A). Therefore, we examined whether lysine to arginine substitutions at the acetylation sites influence the accumulation of RAD52 at DSB sites. The RAD52 (13xR) mutant (with arginine substituted for lysine at the 13 identified acetylated lysine sites, including 3 substitutions in the C-terminal NLS sequence; S3 Fig) localized to the cytoplasm rather than the nucleus, independently of IR (Fig 8B). Interestingly, the RAD52 (13xR) foci colocalized with γ-tubulin, indicating that RAD52 can localize to the centrosome.

**Fig 9:**
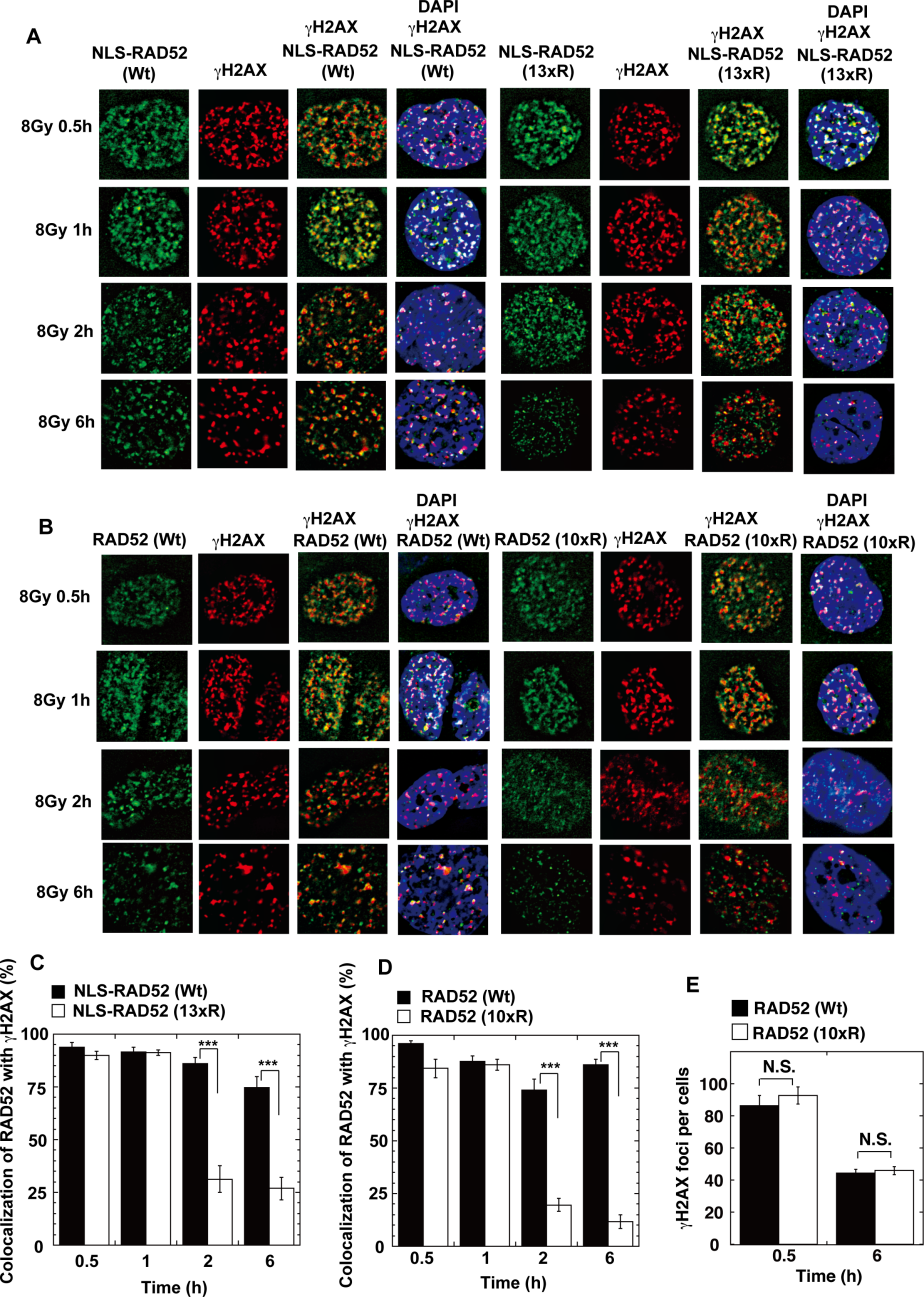
Effect of acetylation-deficient mutations on ionizing radiation-induced foci formation by RAD52. (A, B) MSCs stably expressing FLAG-RAD52-HA proteins were irradiated with γ-rays (8 Gy). At the indicated time after irradiation, the cells were subjected to immunofluorescent staining with an anti-HA (green) antibody, an anti-γH2AX (red) antibody, and DAPI (blue). (A) MSCs expressing NLS-RAD52 (Wt) or NLS-RAD52 (13xR) were used. (B) MSCs expressing RAD52 (Wt) or RAD52 (10xR) were used. (C, D) The percentages of RAD52 foci colocalized with γH2AX were calculated, as described in the Supporting Materials and Methods. Error bars indicate the standard error of the mean. Asterisks indicate statistically significant differences between the indicated pairs of groups (***, *p*<0.001 by t-test). (E) The number of γH2AX foci per cell was counted in MSCs expressing RAD52 (Wt) or RAD52 (10xR) at the indicated time after irradiation with γ-rays (8 Gy), as described in the Supporting Materials and Methods (N.S., not significant by t-test).

To examine the acetylation-deficient effect of the RAD52 (13xR) mutant in the nucleus, we constructed a RAD52 (13xR) mutant with an N-terminally fused NLS (NLS-RAD52 [13xR]; S3 Fig). NLS-RAD52 (13xR) was detected in the nucleus, but did not colocalize with γH2AX in MRC5 cells at 6 h after IR (Fig 8C). We next examined the kinetics of the colocalization of RAD52 (Wt) and RAD52 acetylation-deficient mutants with γH2AX in irradiated MSCs. The control NLS-RAD52 (Wt) foci colocalized with γH2AX for 6 h after IR. In contrast, the NLS-RAD52 (13xR) foci initially colocalized with γH2AX, but then dissociated from γH2AX within 2 h after IR (Fig 9A and 9C). The RAD52 C-terminal region, including the acetylation sites K411, 412, and 414, is known to be essential for the nuclear localization of RAD52. Therefore, we next used the RAD52 (10xR) mutant harboring a normal NLS sequence for analyzing the acetylation effects of RAD52 on IR-induced foci-formation at DSB sites in cells. The RAD52 (10xR) mutant foci similarly showed an initial colocalization with γH2AX, followed by an early dissociation (Fig 9B and 9D). Strikingly, during the first 30 min or 1 h after γ-irradiation, both NLS-RAD52 (13xR) and RAD52 (10xR) showed clear colocalization with γH2AX (Fig 9A–9D).

In order to confirm the effect of the 10xR mutation on the cellular localization of RAD52 expressed by the native promoter, we generated RAD52 (Wt or 10xR) knock-in cells by using CRISPR/Cas9-mediated genome editing. The DNA donor plasmid shown in S9 Fig was constructed by the Multisite Gateway method, and was co-transfected with CRISPR Nuclease Vector plasmids into HeLa pDR-GFP or HEK293 cells. The expression of the HA-tagged RAD52 proteins from the native promoter was confirmed by an immunoblotting analysis (S9F and S9G Fig). We then used the knock-in cells for an immunostaining analysis. In order to deplete the RAD52 protein expressed from the untargeted allele, the knock-in cells were treated with an siRNA targeted to the 3'UTR region of RAD52 (S7C Fig). Consistent with the results described above, the colocalization of RAD52 with γH2AX foci was decreased in the 10xR mutant at 6h after irradiation, whereas both the Wt and 10xR RAD52 proteins colocalized with γH2AX at 1h after irradiation (S10A and S10B Fig).

**Fig 10:**
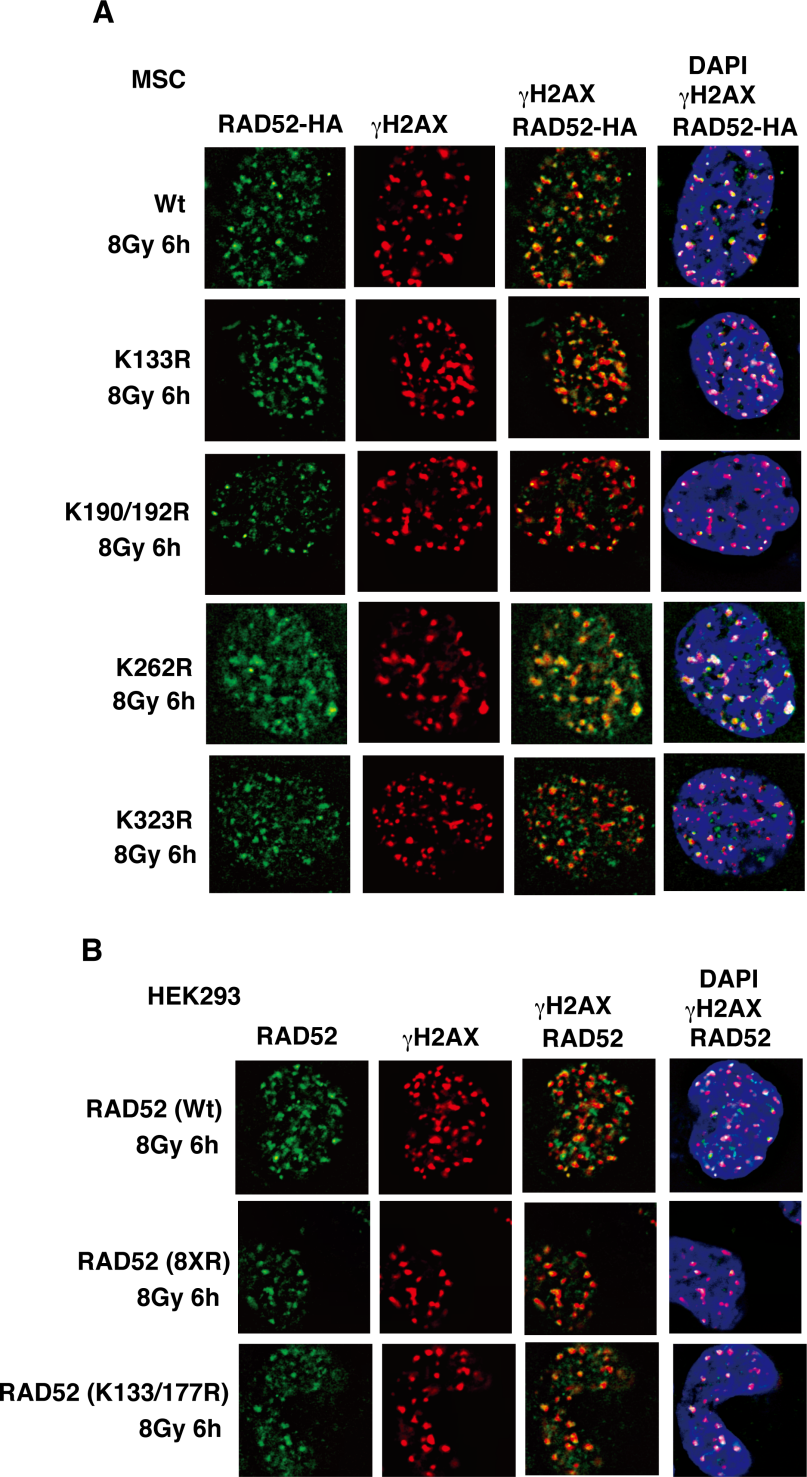
IR-induced foci formation of RAD52 acetylation-site mutant proteins. (A, B) MSCs (A) or T-Rex-293 (HEK293) cells (B) expressing FLAG-RAD52 (Wt or the indicated mutants)-HA were irradiated with γ-rays (8 Gy), and subjected to immunofluorescent staining 6 h after irradiation. An anti-HA (green) antibody, an anti-γH2AX (red) antibody, and DAPI (blue) were used for immunofluorescent staining.

To clarify the critical acetylation sites involved in this colocalization defect, we further examined various RAD52 proteins mutated in several functional domains, such as the highly conserved region (K133R, K133/K177R), the RPA binding region (K262R), and the RAD51 binding region (K323R), and also mutated residues outside the domains (190/192R). We also used the RAD52 (8xR) mutant containing multiple mutations, except in the highly conserved region and the C-terminal NLS region (S3 Fig). In contrast to the results obtained with the NLS-RAD52 (13xR) and RAD52 (10xR) mutants, the colocalization of these RAD52 mutants (K133R, K133/177R, K190/192R, K262R, K323R, and 8xR) with γH2AX was not affected after γ-irradiation (Fig 10A and 10B), indicating that this defect is associated with all of the acetylation sites except for those in the C-terminal NLS region. These findings demonstrate that RAD52 acetylation is required for its sustained retention at DSB sites.

### DSB-induced acetylation of RAD52 is triggered by ATM

**Fig 11:**
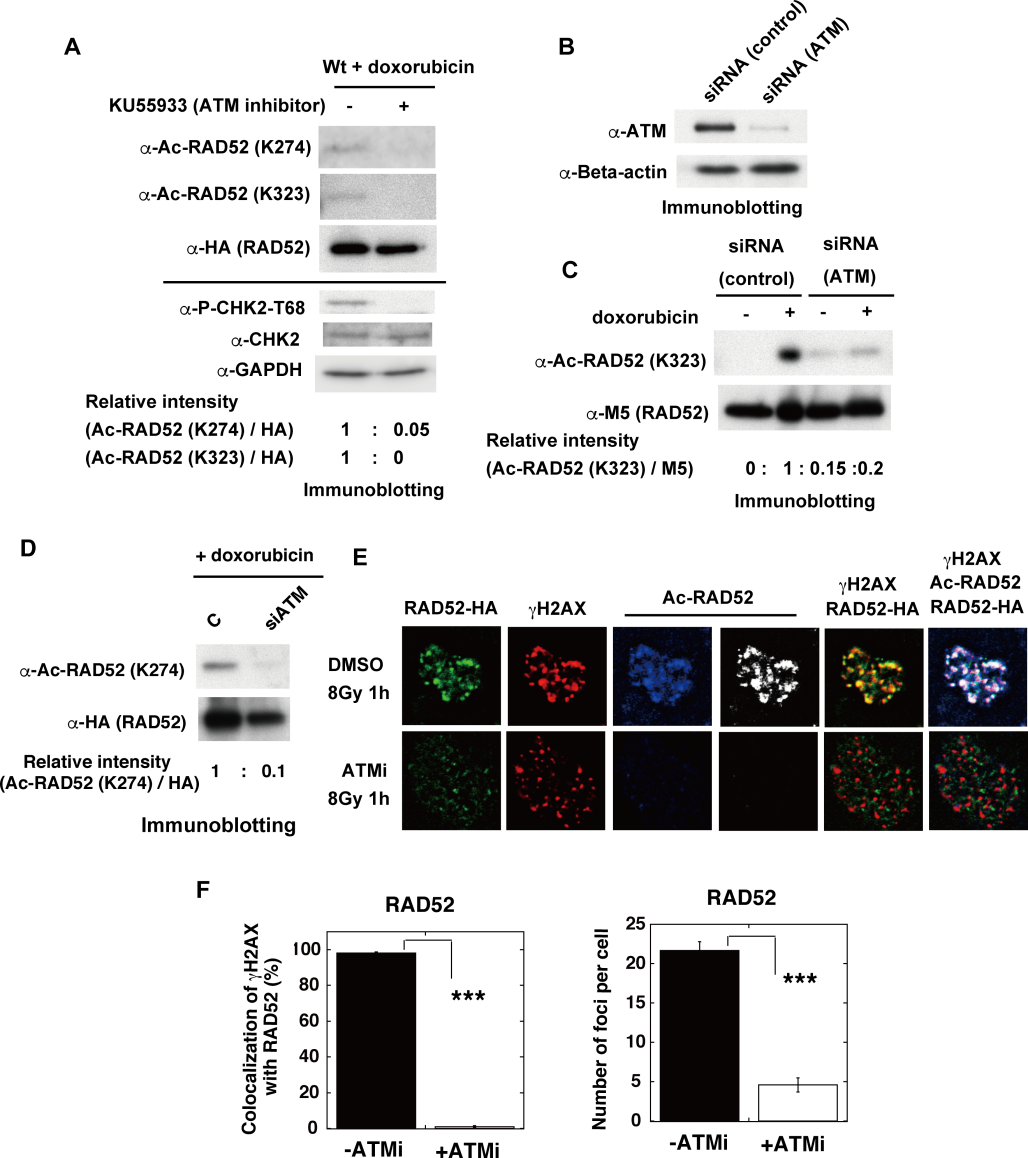
ATM inhibition impedes acetylation of human RAD52 and its recruitment at IR-induced DSB sites. (A) MSCs were transfected with the expression plasmid pT-Rex-DEST30, containing FLAG-RAD52 (Wt)-HA. Cells were treated with doxorubicin for 2 h at 24 h after transfection. Where indicated, KU55933 (ATMi) was added to the cells at 1 h before the addition of doxorubicin. Immunoprecipitated FLAG-RAD52-HA proteins from the cell extracts were subsequently subjected to immunoblotting analyses with anti-Ac-RAD52 (K274), anti-Ac-RAD52 (K323) and anti-HA antibodies. The cell extracts before the immunoprecipitation were also subjected to immunoblotting analyses with anti-phospho-CHK2-T68, anti-CHK2, and anti-GAPDH antibodies. The relative band intensities normalized to those of the HA bands are shown. (B, C, D) MRC5 V1 cells were co-transfected with pT-Rex-DEST30 containing FLAG-RAD52 (Wt)-HA and either control or ATM-specific siRNA, as described in the Supporting Materials and Methods. Cell extracts were prepared 24 h after transfection. (B) Cell extracts were subjected to immunoblotting analyses with the indicated antibodies. (C, D) Where indicated, the cells were treated with doxorubicin for 2 h before preparing the cell extracts. Immunoprecipitated FLAG-RAD52-HA proteins from the cell extracts were subjected to immunoblotting analysis with the indicated antibodies. The relative band intensities normalized to those of the M5 bands (C) or to the HA bands (D) are shown. (E, F) Recruitment of RAD52 to DSBs is dependent on ATM. (E) T-Rex-293 cells expressing FLAG-RAD52 (Wt)-HA were irradiated with γ-rays (8 Gy). The KU55933 solution or the same volume of DMSO was added to the cells 1 h before irradiation. At 1 h after irradiation, the cells were subjected to immunofluorescent staining. Immunofluorescent images with anti-HA (green), anti-γH2AX (red), and anti-acetylated RAD52 at K323 (blue or white) antibodies. (F) The percentages of γH2AX foci colocalized with RAD52 in Fig 11E were calculated, as described in the Supporting Materials and Methods, and are shown in the graph. The numbers of protein foci per cell are also shown in the graph. Error bars indicate the standard error of the mean. Asterisks indicate statistically significant differences between the indicated pairs of groups (***, p<0.001 by t-test).

ATM is a pivotal mediator of signal transduction in response to DSBs. As RAD52 acetylation was induced by DSBs, we examined whether its acetylation was triggered by ATM. KU55933 is a potent and specific ATM kinase inhibitor (ATMi). To verify the functionality of the inhibitor, we observed that the ATM-mediated phosphorylation of CHK2 on threonine 68 (T68) was inhibited in the KU55933-treated cells used in our present study. We found that the doxorubicin-induced acetylation of RAD52 was inhibited by the KU55933 (ATMi) treatment (Fig 11A). Consistent with this result, the knockdown of ATM by siRNA treatment decreased the doxorubicin-induced acetylation of RAD52 (Fig 11B, 11C and 11D).

**Fig 12:**
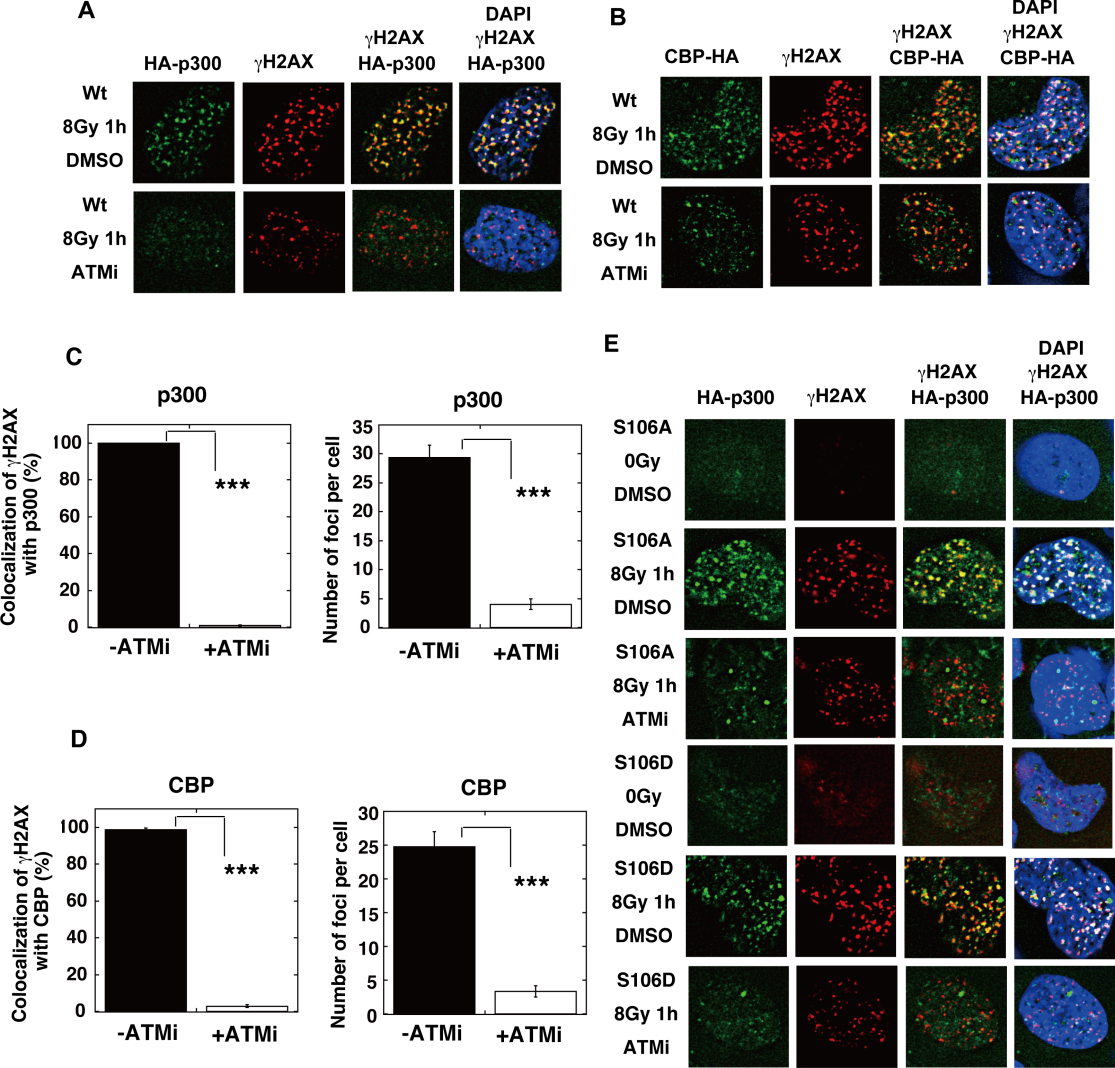
ATM inhibition impedes the recruitment of p300/CBP at IR-induced DSB sites. (A, B) MRC5V1 cells were transfected with the expression plasmid for wild-type (Wt) HA-p300 (A) or CBP-HA (B). (E) MRC5V1 cells were transfected with the expression plasmid for either HA-p300 S106A or HA-p300 S106D. (A, B, E) At 24 h after transfection, the KU55933 (ATMi) solution or the same volume of DMSO was added to cells 1 h before irradiation with γ-rays (8 Gy). At 1 h after irradiation, the cells were subjected to immunofluorescent staining. Immunofluorescent images with an anti-HA (green) antibody, an anti-γH2AX (red) antibody, and DAPI (blue). (C, D) The percentages of γH2AX foci colocalization with p300 (C) or CBP (D) in Fig 12A and 12B were calculated, as described in the Supporting Materials and Methods, and are shown in the graphs. The numbers of protein foci per cell are also shown in the graphs. Error bars indicate the standard error of the mean. Asterisks indicate statistically significant differences between the indicated pairs of groups (***, p<0.001 by t-test).

**Fig 13:**
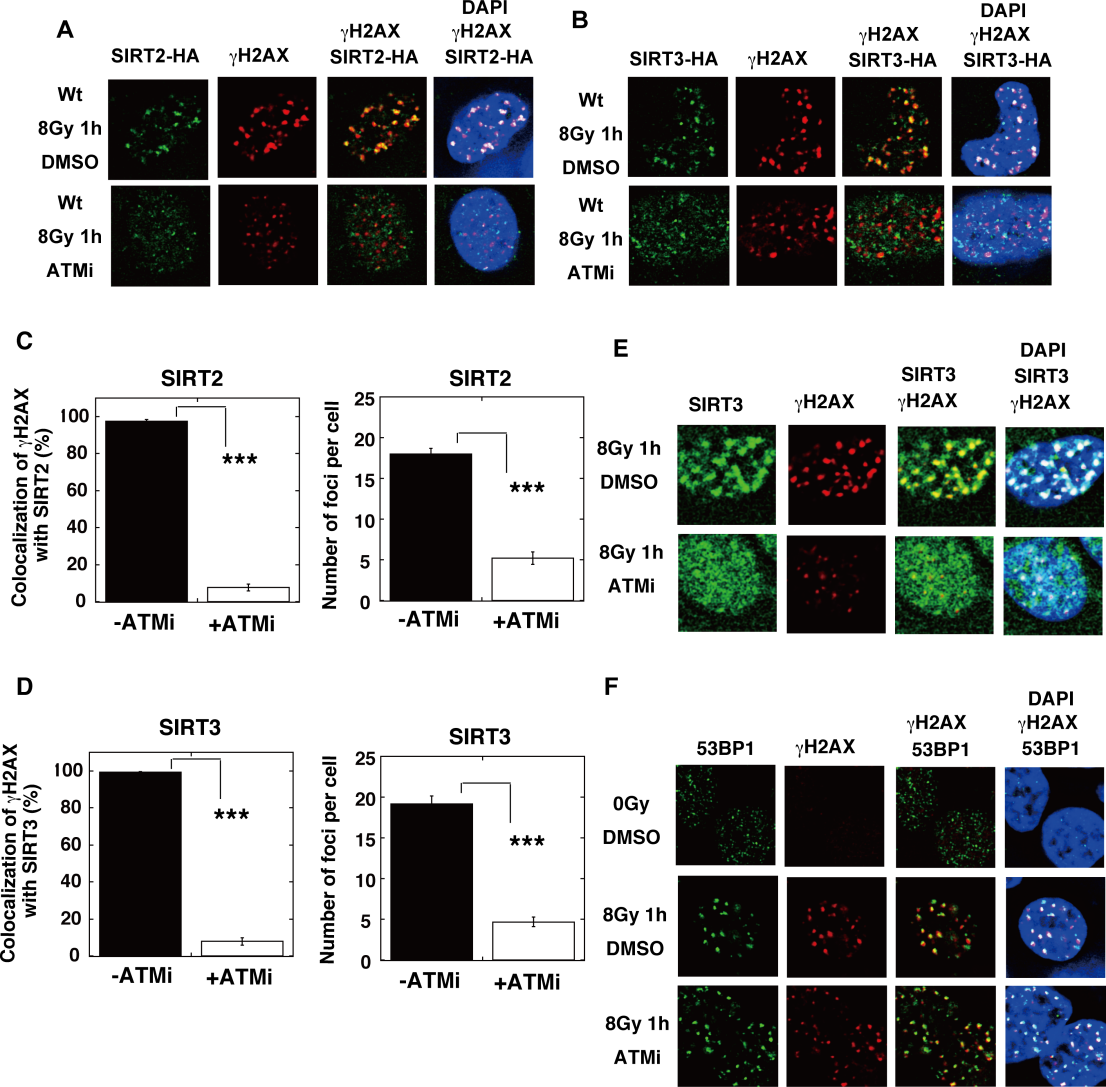
ATM inhibition impedes the recruitment of SIRT2/SIRT3, but not 53BP1, at IR-induced DSB sites. (A, B) T-Rex-293 cells expressing SIRT2-HA (A), or SIRT3-HA (B) were irradiated with γ-rays (8 Gy). The KU55933 (ATMi) solution or the same volume of DMSO was added to the cells 1 h before irradiation. At 1 h after irradiation, the cells were subjected to immunofluorescent staining. Immunofluorescent images with an anti-HA (green) antibody, an anti-γH2AX (red) antibody, and DAPI (blue). (C, D) The percentages of γH2AX foci colocalization with SIRT2 (C) or SIRT3 (D) in Fig 13A and 13B were calculated, as described in the Supporting Materials and Methods, and are shown in the graphs. The numbers of protein foci per cell are also shown in the graphs. Error bars indicate the standard error of the mean. Asterisks indicate statistically significant differences between the indicated pairs of groups (***, p<0.001 by t-test). (E, F) The KU55933 solution or the same volume of DMSO was added to T-Rex-293 cells, 1 h before irradiation with γ-rays (8 Gy). (E) Cells were subjected to immunofluorescent staining at 1 h after irradiation with an anti-SIRT3 (green) antibody, an anti-γH2AX (red) antibody, and DAPI (blue). (F) Cells were subjected to immunofluorescent staining at 1 h after irradiation with an anti-53BP1 (green) antibody, an anti-γH2AX (red) antibody, and DAPI (blue). As a control, unirradiated cells (0 Gy) were used.

The IR-induced acetylation of RAD52 was also inhibited by the treatment with KU55933 (ATMi) (Fig 11E). Notably, the ATM inhibition with KU55933 (ATMi) did not completely diminish the γH2AX foci formation, consistent with the previous findings that the IR-induced phosphorylation of H2AX can be redundantly affected by ATM or DNA-PK [40]. These γH2AX foci did not colocalize with the RAD52 foci, indicating that the treatment with KU55933 (ATMi) inhibited the accumulation of RAD52 at DSB sites (Fig 11E and 11F). The IR-induced accumulation of both p300 and CBP at DSB sites was inhibited by the treatment with KU55933 (ATMi) in MRC5 cells (Fig 12A–12D). ATM is involved in the DNA damage-induced phosphorylation of p300 at serine 106 (S106) [41]. We used p300 mutant proteins in which S106 was substituted with alanine (S106A) or aspartic acid (S106D), which mimics the phosphorylated state (Fig 12E). Both mutant proteins colocalized with γH2AX after IR, suggesting that the ATM-mediated phosphorylation of p300 at the S106 site is not involved in its colocalization at DSB sites. The colocalization of these two mutant proteins with γH2AX was inhibited by KU55933 (ATMi). Interestingly, the radiation-induced accumulation of SIRT2 and SIRT3 at DSB sites was also inhibited by the treatment with KU55933 (ATMi) (Fig 13A–13E). These ATM-associated events were not caused by non-specific effects, because the colocalization of 53BP1 with γH2AX was not inhibited by KU55933 (ATMi) (Fig 13F). The number of foci of RAD52, p300, CBP, SIRT2, and SIRT3 decreased in the presence of KU55933 (ATMi) (Figs 11F, 12C, 12D, 13C and 13D), whereas the cellular levels of these proteins did not significantly change (S8A–S8E Fig). Thus, ATM inhibition prevents the accumulation of RAD52, p300/CBP, and SIRT2/SIRT3 at DSB sites, thereby causing the intra-cellular diffusion of these proteins, even after IR treatment. Therefore, our results suggest that the interaction of RAD52 with p300 and CBP will be reduced by ATM inhibition, thereby decreasing the acetylation of RAD52. Taken together, our results indicate that the DSB-induced acetylation of RAD52 occurs in the vicinity of DSB sites in an ATM-associated manner.

### RAD52 acetylation is required for sustained colocalization of RAD51 at DSB sites

**Fig 14:**
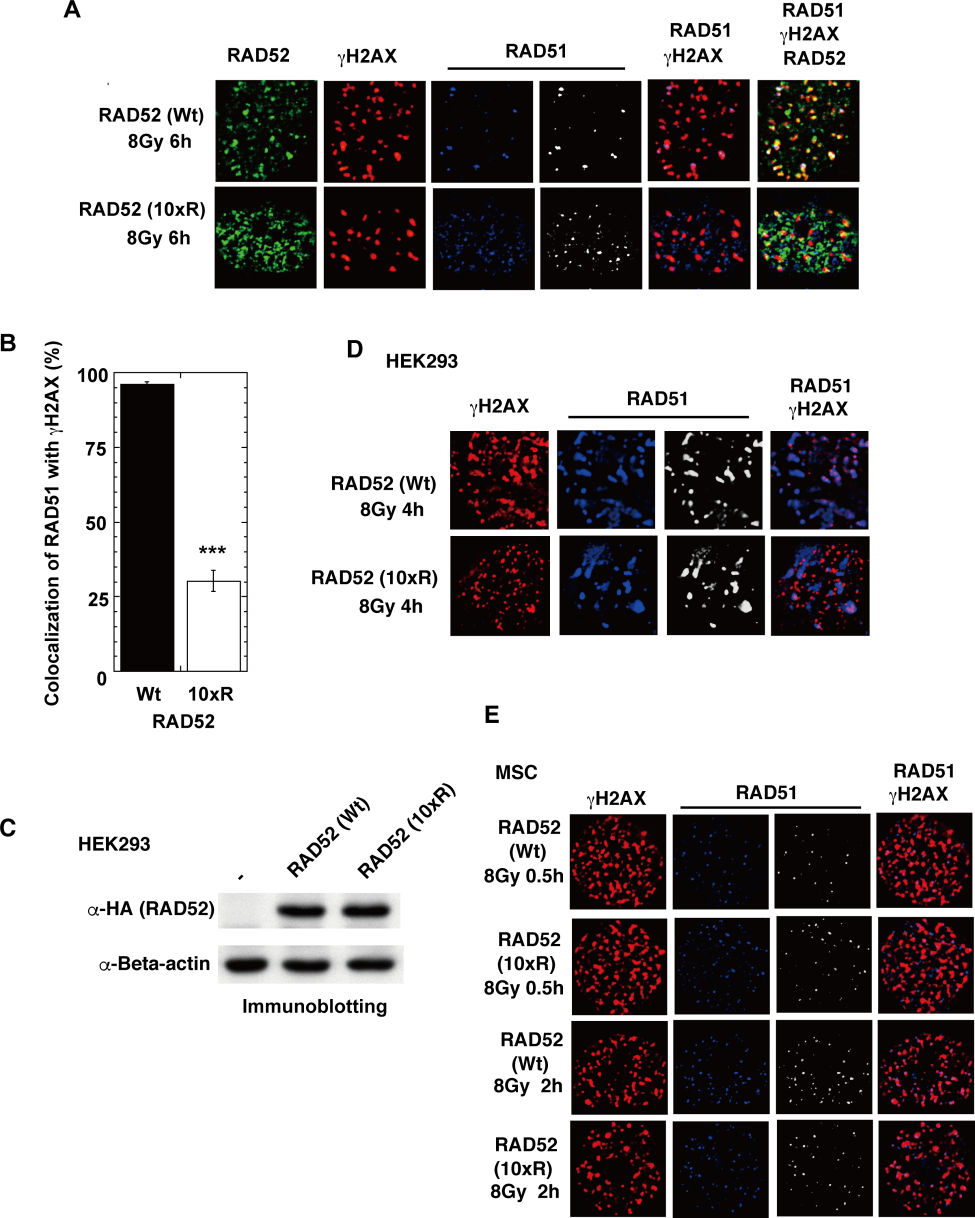
Colocalization of RAD51 foci at DSB sites is inhibited in cells expressing RAD52 (10xR) acetylation-deficient mutant protein. (A, B) MSCs stably expressing the indicated FLAG-RAD52-HA proteins were irradiated with γ-rays (8 Gy), and subjected to immunofluorescent staining 6 h after irradiation. (A) Immunofluorescent images with anti-HA (green), anti-γH2AX (red), and anti-RAD51 (blue) antibodies are shown. (B) The percentages of RAD51 foci colocalized with γH2AX were calculated, as described in the Supporting Materials and Methods. Error bars indicate the standard error of the mean. Asterisks indicate statistically significant differences between the FLAG-RAD52 (Wt)-HA expressing cells and the FLAG-RAD52 (10xR)-HA expressing cells (***, *p*<0.001 by t-test). (C) T-Rex-293 (HEK293) cells stably integrated with pT-Rex-DEST30 containing FLAG-RAD52 (Wt or 10xR)-HA were cultured in the absence of a tetracycline inducer. As a negative control (-), T-Rex-293 cells that did not contain the expression vector were used. Cell extracts were subjected to immunoblotting analyses with the indicated antibodies. (D) T-Rex-293 (HEK293) cells expressing FLAG-RAD52 (Wt or 10xR)-HA were irradiated with γ-rays (8 Gy), and subjected to immunofluorescent staining 4 h after irradiation. Immunofluorescent images with anti-γH2AX (red) and anti-RAD51 (blue or white) antibodies are shown. (E) MSCs stably expressing FLAG-RAD52 (Wt or 10xR)-HA were irradiated with γ-rays (8 Gy), and subjected to immunofluorescent staining 0.5 or 2 h after irradiation. Immunofluorescent images with anti-γH2AX (red) and anti-RAD51 (blue or white) antibodies are shown.

**Fig 15:**
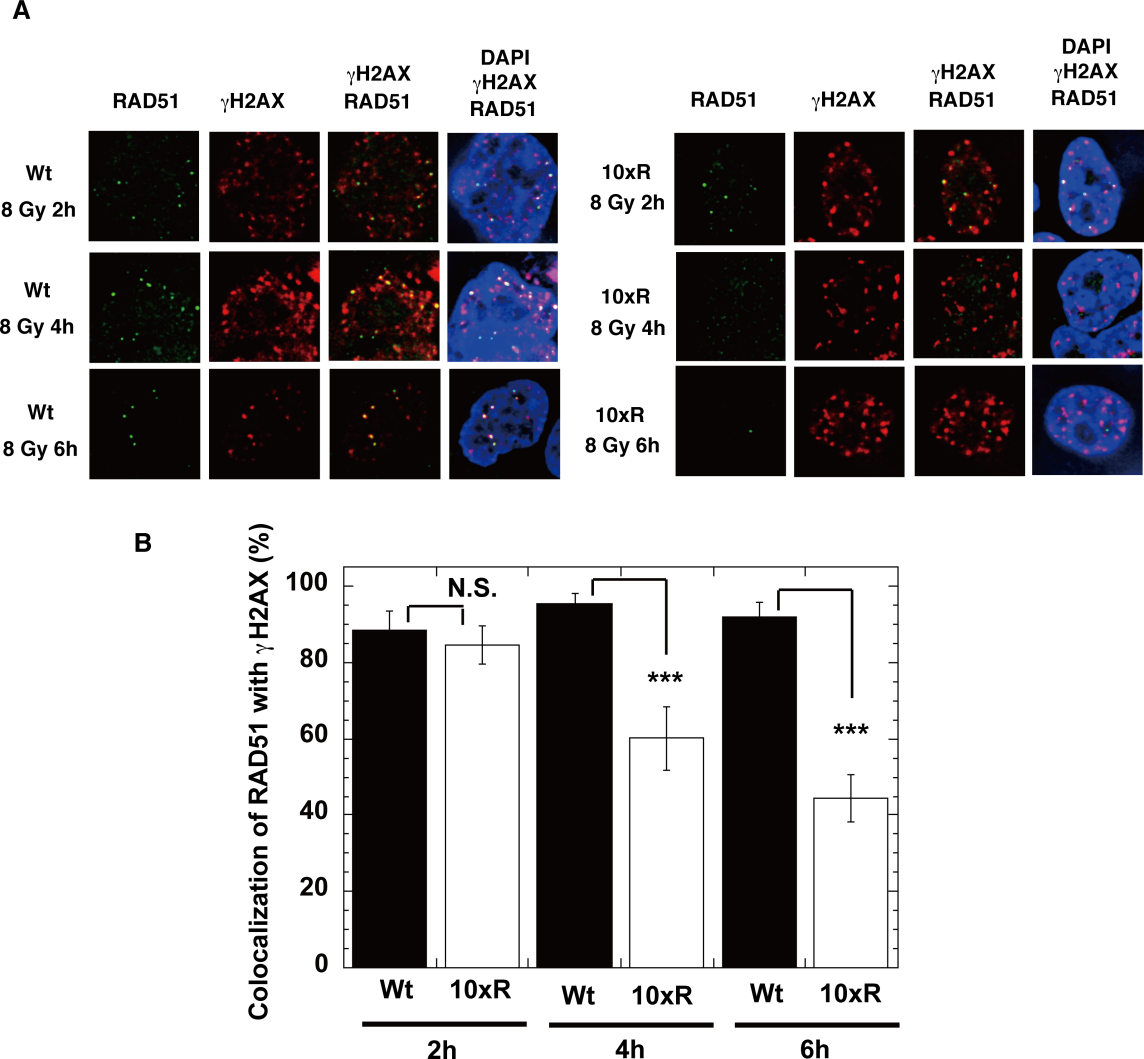
A time course analysis of RAD51 foci colocalization with γH2AX foci in cells expressing RAD52 Wt or 10xR mutant protein. (A, B) RAD52 (Wt or 10xR) knock-in HEK293 cells, as shown in S9 Fig, were used. The cells were treated with siRNA (siRAD52 (3'UTR #1)) 2 days before irradiation. (A) At the indicated time after irradiation with γ-rays (8 Gy), the cells were subjected to immunofluorescent staining with an anti-RAD51 (green) antibody, an anti-γH2AX (red) antibody, and DAPI (blue). (B) The percentages of RAD51 foci colocalized with γH2AX were calculated, as described in the Supporting Materials and Methods. Error bars indicate the standard error of the mean. Asterisks indicate statistically significant differences between the FLAG-RAD52 (Wt)-HA expressing cells and the FLAG-RAD52 (10xR)-HA expressing cells (N.S., not significant; ***, *p*<0.001 by t-test).

We next examined whether RAD52 acetylation influences the accumulation of the RAD52-associated proteins, RPA and RAD51, at DSB sites. Expression of the RAD52 (10xR) mutant protein disrupted the IR-induced colocalization of RAD51 with γH2AX at 6 h in MSCs (Fig 14A and 14B) and at 4 h in HEK293 cells (Fig 14C and 14D). Furthermore, RAD52 (10xR) mutant expression in MSCs did not affect RAD51 foci formation or colocalization with γH2AX from 0.5 to 2 h after IR (Fig 14E). A time course experiment revealed that RAD51 colocalized with γH2AX at 2 h after irradiation in both RAD52 (Wt) and RAD52 (10xR)-expressing HEK293 knock-in cells, with no significant difference (Fig 15A and 15B). Thereafter, at 4 and 6 h after irradiation, the colocalization decreased only in the RAD52 (10xR)-expressing cells (Fig 15A and 15B). These findings suggest that RAD52 acetylation is dispensable for the initial loading of RAD51 at DSB sites, but is required for the sustained retention of RAD51 at DSB sites.

**Fig 16:**
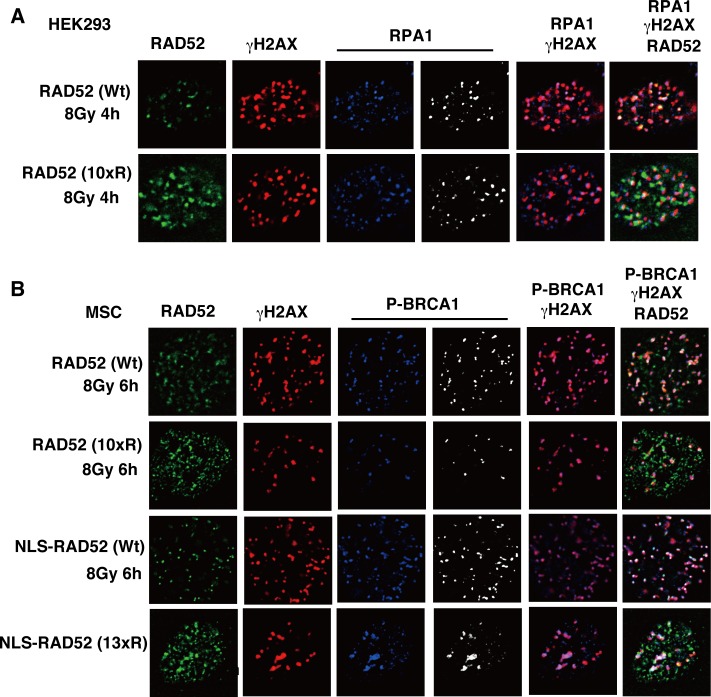
The RAD52 (10xR) mutant protein does not inhibit the formation of replication protein A (RPA) and BRCA1 foci. (A) T-Rex-293 (HEK293) cells expressing FLAG-RAD52 (Wt or 10xR)-HA were irradiated with γ-rays (8 Gy), and subjected to immunofluorescent staining 4 h after irradiation. Immunofluorescent images with anti-HA (green), anti-γH2AX (red), and anti-RPA1 (blue or white) antibodies are shown. (B) MSCs stably expressing the indicated FLAG-RAD52-HA proteins were irradiated with γ-rays (8 Gy), and subjected to immunofluorescent staining 6 h after irradiation. Immunofluorescent images with anti-HA (green), anti-γH2AX (red), and anti-phospho-BRCA1 at Ser1524 (blue or white) antibodies are shown.

If the DNA resection is affected by RAD52 acetylation, then RPA foci formation should also be affected. However, the expression of the RAD52 (10xR) mutant protein did not affect the colocalization of RPA with γH2AX (Fig 16A). Therefore, our result suggests that DNA resection is not affected by RAD52 acetylation. Previously, BRCA1 was demonstrated to function in the loading of RAD51 at DSB sites via the PALB2-mediated interaction with BRCA2 [42]. IR-induced phosphorylated BRCA1 foci were observed at DSB sites in RAD52 (10xR) and NLS-RAD52 (13xR)-expressing cells (Fig 16B). These findings suggest that the non-acetylated RAD52 protein disturbs the colocalization of RAD51 at DSB sites, but does not influence BRCA1 foci formation. These results are consistent with the aforementioned finding that the initial loading of RAD51 at DSB sites was not affected by RAD52 acetylation.

### Acetylation of RAD52 is required for HR repair

**Fig 17:**
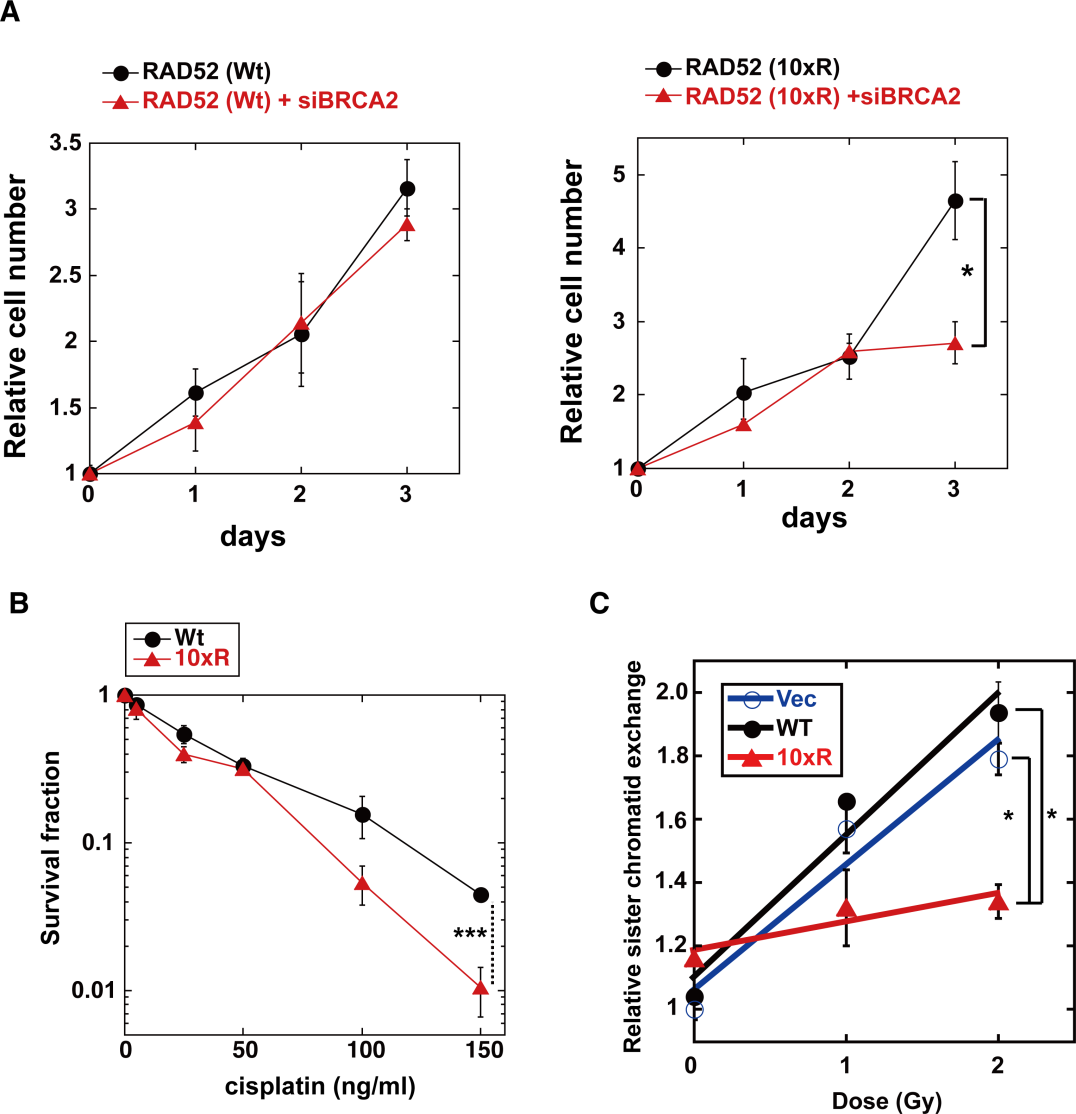
Effect of RAD52 (10xR) acetylation-deficient mutant protein on cell growth, cell survival and IR-induced sister chromatid exchange. (A, B) T-Rex-293 (HEK293) cells stably expressing the Wt or 10xR FLAG-RAD52-HA protein were cultured in the absence of the Tet inducer. (A) Endogenous RAD52 was depleted by siRNA treatment with siRAD52 (3'UTR#1). Where indicated, the cells were also subjected to an siRNA treatment with the mixture of siBRCA2 (#1, #2 and #3) at day 0. The cell growth was examined as described in the Supporting Materials and Methods section. The graph shows the mean values and the standard error of the mean from triplicate samples. Asterisks indicate statistically significant differences (*, *P*<0.05 by t-test). (B) Cells were treated with the indicated concentration of cisplatin. Cell survival was assayed as described in the Supporting Materials and Methods section. Means with standard errors of four experiments are shown. Asterisks indicate statistically significant differences (***, *p*<0.001 by t-test). (C) T-Rex-293 (HEK293) cells stably integrated with pT-Rex-DEST30 containing FLAG-RAD52 (Wt)-HA, FLAG-RAD52 (10xR)-HA or its empty vector were cultured in the absence of the Tet inducer. The cells were exposed to X-ray radiation. The sister chromatid exchange assay was performed, as described in the Materials and Methods section. In independent experiments, 50 cells were counted for each condition. The graph shows the mean values and the standard error of the mean from two independent experiments. Asterisks indicate statistically significant differences (*, *P*<0.05 by t-test).

RAD52 depletion is reportedly synthetically lethal with the BRCA2 deficiency, and inhibits cell growth in BRCA2-deficient cells [11]. Therefore, we examined whether BRCA2 depletion also inhibits cell growth in RAD52 (10xR)-expressing cells. BRCA2 depletion inhibited cell growth in RAD52 (10xR)-expressing cells, but not in RAD52 (Wt)-expressing cells (Fig 17A).

Expression of the RAD52 (10xR) mutant did not affect the γH2AX foci formation after irradiation (Fig 9B and 9E). This result might be due to the existence of a backup DSB repair system by the NHEJ pathway, and is consistent with the previous report showing that the inactivation of mouse RAD52 reduces HR, but does not affect the resistance to ionizing radiation [7]. However, the NHEJ pathway is dispensable for the repair of cross-linking DNA damage, but the HR pathway is required for its repair with the Fanconi anemia DNA repair pathway [44]. In the survival assay of cells treated with the DNA cross-linker cisplatin, the RAD52 (10xR)-expressing cells were more sensitive to cisplatin than the RAD52 (Wt)-expressing cells (Fig 17B). These results suggest that the acetylation of RAD52 is involved in HR repair. Therefore, we next examined whether the expression of the RAD52 (10xR) mutant affects the HR efficacy. We quantified IR-induced sister chromatid exchanges in HEK293 cells expressing an empty vector or a vector encoding RAD52 (Wt) or RAD52 (10xR). Expression of RAD52 (Wt) did not influence the IR-induced sister chromatid exchanges, whereas the RAD52 (10xR) mutant expression decreased the frequency of sister chromatid exchanges (Fig 17C).

**Fig 18:**
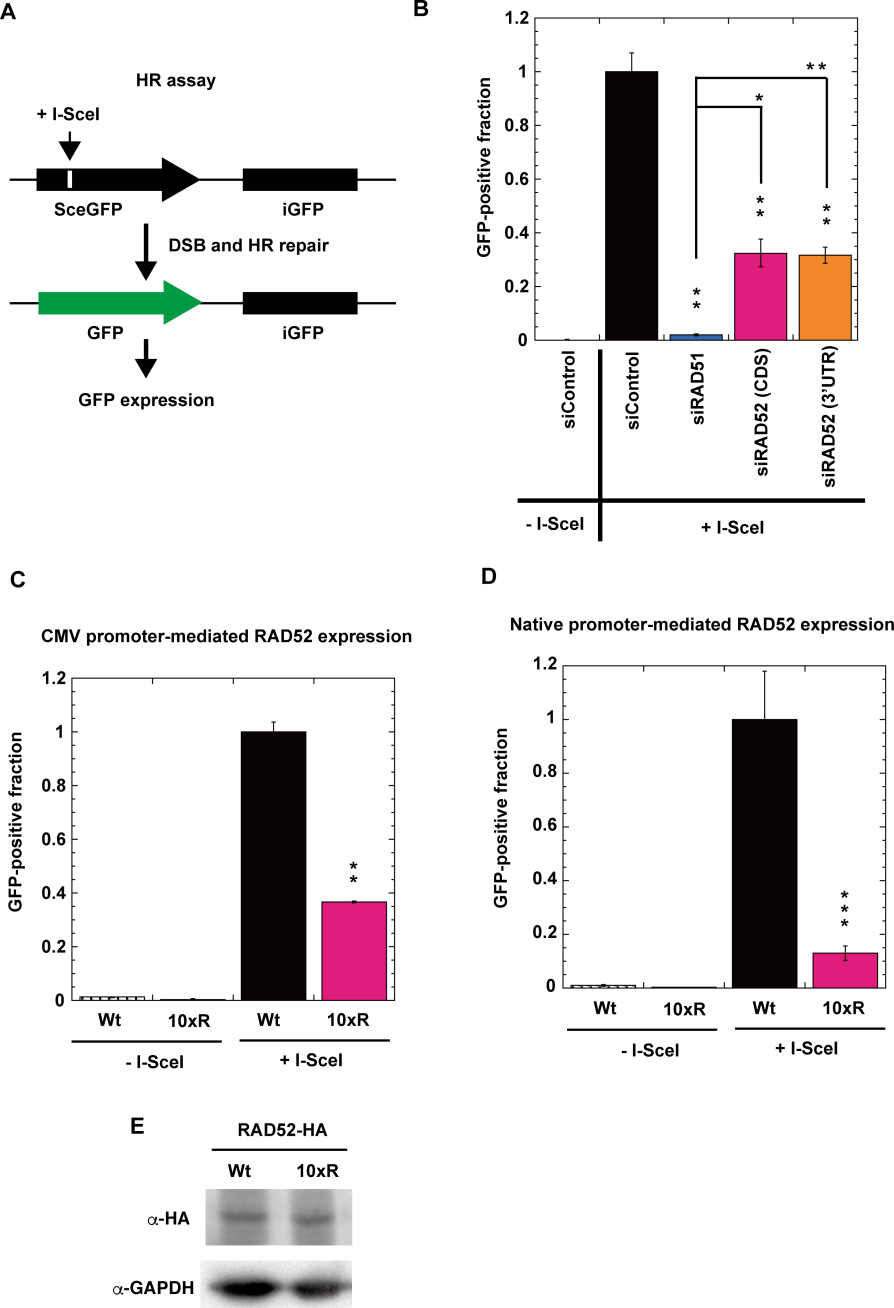
Acetylation of RAD52 is required for HR repair. (A) Schematic representation of the I-SceI-induced HR repair assay. GFP-positive cells are produced by HR repair at an I-SceI-induced specific DSB site. (B) HeLa pDR-GFP cells were treated with siRAD51 (#1), siRAD52 (CDS #1), siRAD52 (3'UTR #1) or negative siRNA control. (C) HeLa pDR-GFP cells stably integrated with pT-Rex-DEST30 containing FLAG-RAD52 (Wt)-HA or FLAG-RAD52 (10xR)-HA were used. (D) RAD52 (Wt or 10xR) knock-in HeLa pDR-GFP cells, as shown in S9 Fig, were used. (C, D) Cells were treated with siRAD52 (3'UTR #1) in order to deplete the non-tagged endogenous RAD52 protein. (B, C, D) After inducing I-SceI, GFP-positive cells were examined as described in the Supporting Materials and Methods. Error bars indicate the standard error of the mean from three samples (B, C) or twelve samples (D). (B) Asterisks indicate statistically significant differences of each protein-depleted sample, as compared with the control (+I-SceI) (*, *P*<0.05 and **, *P*<0.01 by t-test). All samples connected by lines were also compared. (C, D) Asterisks indicate statistically significant differences between Wt (+I-SceI) and 10xR (+I-SceI) (**, *P*<0.01 and ***, *p*<0.001 by t-test). (E) Immunoblotting analyses of RAD52-HA expression in the cells used in Panel C with anti-HA and anti-GAPDH antibodies.

In order to confirm the requirement of RAD52 acetylation for HR repair, we used a reporter assay with a cell line bearing a direct repeat green fluorescent protein (DR-GFP) reporter cassette [43,45,46,47]. The DR-GFP reporter cassette comprises two inactive GFP genes in a direct repeat orientation. One of the genes, SceGFP, contains an I-SceI cleavage site that is absent in the human genome. The other gene, iGFP, comprises the internal GFP fragment. HR repair between SceGFP and iGFP is induced when a specific DSB at the I-SceI site is introduced by the expression of I-SceI endonuclease. Since HR repair generates an intact GFP gene, the HR repair efficiency can be monitored as the frequency of GFP-positive cells (Fig 18A).

Using this HR assay system, we first examined the impact of the depletion of RAD52, RAD51, and BRCA2 proteins. These proteins were efficiently depleted by siRNA treatment (S7C–S7G Fig). The depletion of RAD51 almost completely inhibited I-SceI-induced HR repair, as expected (Fig 18B). The HR repair efficiency was reduced more by the BRCA2 depletion than by the RAD52 depletion (S11 Fig), which is consistent with the notion that BRCA2 and RAD52 function in different pathways of RAD51-dependent HR repair [11]. Then, we examined the impact of the RAD52 acetylation-deficient mutation on HR repair (Fig 18C and 18D). We used two types of HeLa pDR-GFP cells, expressing either the HA-tagged RAD52 (Wt) or RAD52 (10xR) protein. The RAD52 (Wt) and RAD52 (10xR) proteins were expressed by the native and CMV promoters, respectively, and the expression levels of the proteins were almost the same in both types of cells (Fig 18E and S9F Fig). The endogenous untagged RAD52 protein was depleted by a treatment with an siRNA targeted to the 3'UTR region of RAD52 (S7C Fig). In both cell lines, the expression of the RAD52 (10xR) protein inhibited HR repair (Fig 18C and 18D). Collectively, these findings demonstrate that the acetylation of RAD52 is required for HR repair.

### Acetylation mimic RAD52 (10xQ) mutation affects its protein-protein interactions in yeast two-hybrid analysis

**Fig 19:**
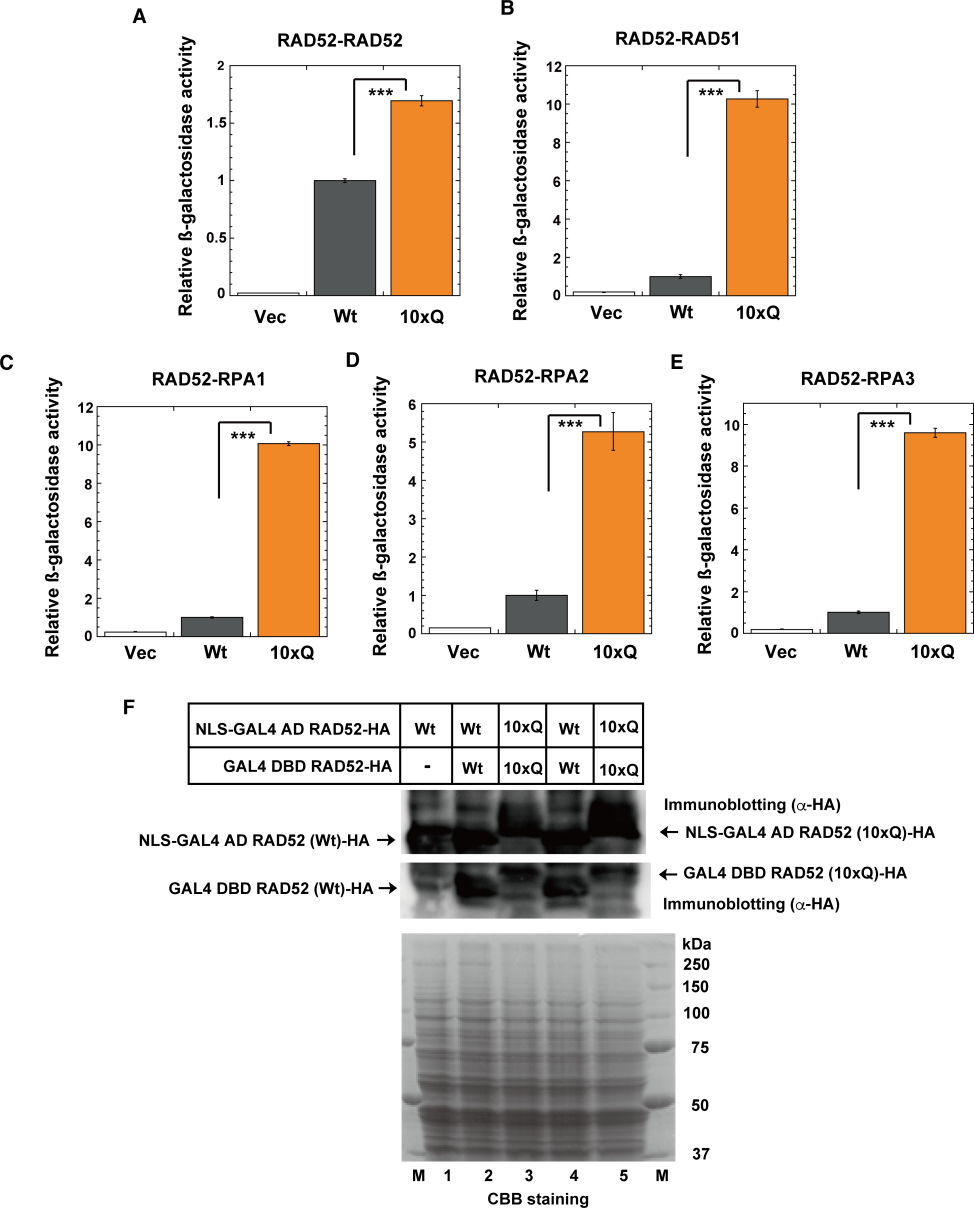
RAD52 10xQ mutation affects protein-protein interactions in yeast two-hybrid analysis. (A) The β-galactosidase activities in the yeast two-hybrid analysis were examined, as described in the Supporting Materials and Methods. (A) Interactions of GAL4 DBD-fused RAD52 with NLS-GAL4 AD-fused RAD52 (A), RAD51 (B), RPA1 (C), RPA2 (D) and RPA3 (E) were examined in yeast cells expressing the fused Wt or 10xQ RAD52 protein. As a negative control, the empty vector used for the expression of GAL4 DBD-fused RAD52, pDEST32, was used. The relative β-galactosidase activities compared to that of RAD52 (Wt) are shown. The graph shows the mean values and the standard error of the mean from at least three samples. Asterisks indicate statistically significant differences (***, *p*<0.001 by t-test). (F) Expression of the NLS-GAL4 AD or GAL4 DBD-fused RAD52-HA (Wt or 10xQ) proteins was analyzed by immunoblotting with an anti-HA antibody (upper and middle). In lane 1, the extract from yeast cells containing the empty vector for the expression of the GAL4-DBD-fused protein was used. The same amounts of the yeast cell extracts used for the immunoblotting analysis were also subjected to SDS-PAGE followed by CBB staining, for a loading control (lower). The immunoblotted bands of the 10xQ mutants were slightly upshifted, as compared to those of the Wt, by the 10xQ mutation.

The interaction of human RAD52 with human RAD51 was previously detected in a yeast two-hybrid analysis [15]. The interactions of yeast Rad52 with yeast Rpa1, Rpa2, Rpa3 and Rad51 have also been detected [14]. Therefore, we analyzed the interactions between the human acetylation mimic RAD52 (10xQ) and its target-proteins, using a yeast two-hybrid analysis. Glutamine (Q) is widely used to mimic acetylated lysine (K), because the effect of the lysine-to-glutamine substitution is similar to the effect of the acetylation of lysine [48]. The yeast cells lacked p300 and CBP. Therefore, we constructed the plasmids expressing either GAL4-DBD or the NLS-GA4-AD-fused RAD52 (10xQ) mutant for a yeast two-hybrid analysis (S3 Fig). When the NLS-GAL4-activation domain (AD)-fused protein interacts with the GAL4-DNA binding domain (DBD)-fused protein, the reporter gene, His3, is expressed, and the yeast cells show growth on an SC-Leu-Trp-His agar plate containing 25 mM 3-Amino-1,2,4-Triazole (3AT). Interactions of the GAL4-DBD-fused human RAD52 (Wt) with the NLS-GA4-AD-fused human RAD52 (Wt), RAD51, RPA1, RPA2 or RPA3 were observed in the yeast two-hybrid analysis (S12 Fig). Another reporter gene, lacZ, can also be used in this yeast two-hybrid analysis system. Therefore, we quantitatively examined the protein-protein interactions of RAD52, by using a liquid β-galactosidase assay (Fig 19A–19E). Both RAD52 (Wt) and RAD52 (10xQ) were expressed almost equally in yeast cells (Fig 19F). The self-interaction of RAD52 was increased 1.7-fold by the 10xQ mutation (Fig 19A). The interactions of RAD52 with RAD51, RPA1, RPA2 and RPA3 were remarkably increased by the 10xQ mutation (Fig 19B–19E). These results suggest that the interactions of RAD52 with these proteins are enhanced by its acetylation.

## Discussion

### Molecular Mechanisms Controlling HR through Human RAD52 Acetylation

**Fig 20:**
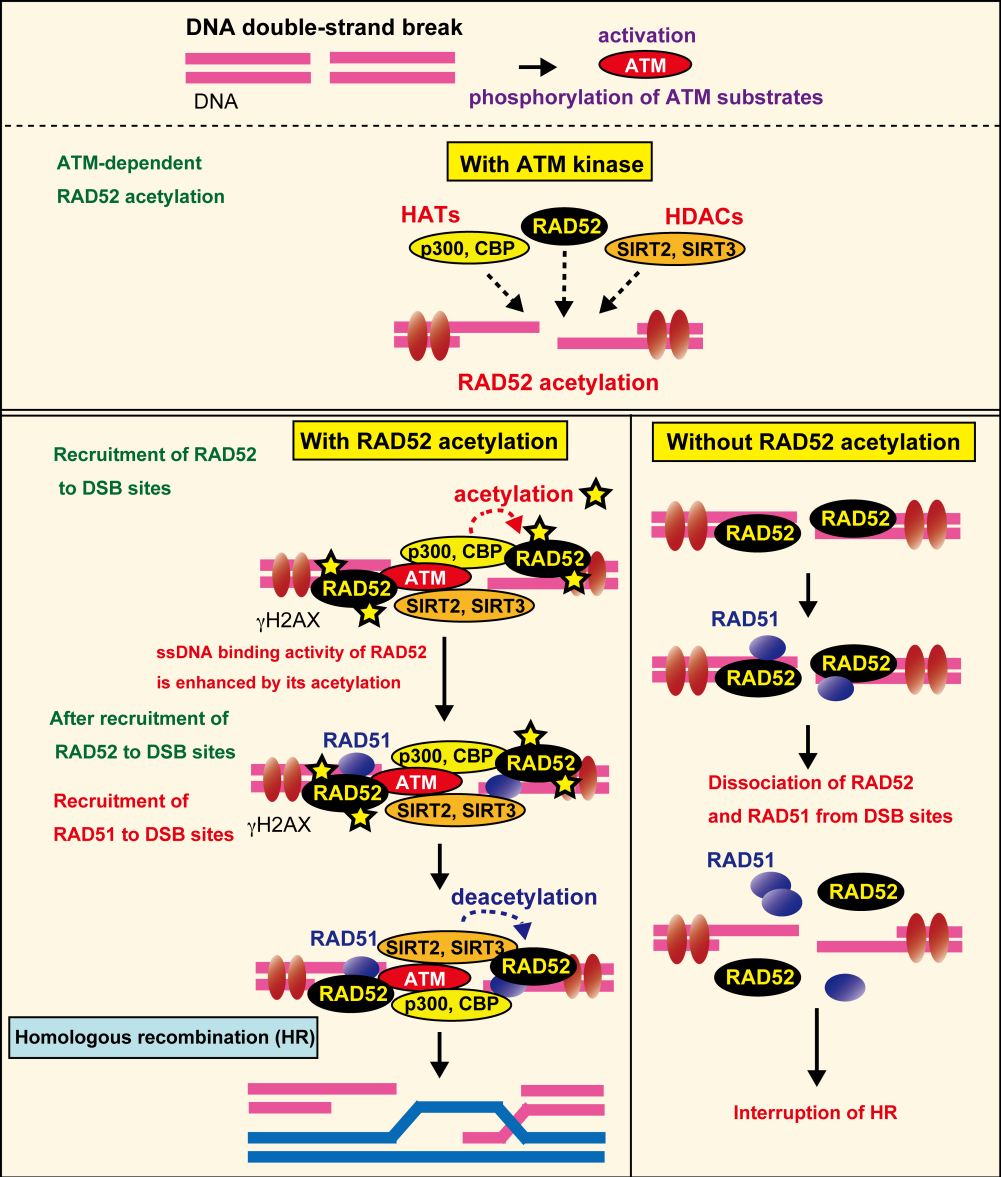
Acetylation of human RAD52 is required for DSB repair by homologous recombination. A summary of our results is shown in the model (see discussion in the main text).

Based on our findings, we propose the following working model (Fig 20). Following DSB formation, human RAD52 and p300/CBP are recruited to DSB sites, and interact with each other near the DSB sites, thereby inducing RAD52 acetylation. Although 13 lysine residues can be acetylated, the acetylation efficiency of each site is probably different. SIRT2 and SIRT3 are also recruited to DSB sites shortly after DSB induction, and deacetylate RAD52. During HR, RAD52 interacts with RPA or DNA. These interactions may prevent the ongoing acetylation of RAD52 by p300/CBP. As a result, the acetylation level of RAD52 is diminished by SIRT2 and SIRT3. The acetylation sites of RAD52 are located in the DNA binding region (K133), and also within regions involved in protein-protein interactions. Indeed, the acetylation-mimicking RAD52 showed increased interactions with RPA and RAD51 in yeast cells (Fig 19B–19E), and acetylated RAD52 binds ssDNA more robustly than non-acetylated RAD52 *in vitro* (S13 Fig). These findings suggested that acetylated RAD52 plays a critical role in the maintenance of RAD51 recruited to DSB sites. Non-acetylated RAD52 dissociates prematurely from the DSB sites, and thus impairs the retention of RAD51 at the DSB site and prevents the completion of HR. The change in the levels and sites of acetylation during HR might control several activities of the multifunctional RAD52 protein.

HR is a multistep process mediated by the concerted actions of many proteins. Regulation of each protein in both positive and negative manners is probably required to achieve the multistep and multi-enzyme processes. At present, the precise molecular mechanisms by which RAD52 exerts its functions in HR have not been elucidated. It is possible, however, that the strength of the RAD52 interactions with DNA and proteins could change during HR, which would enable RAD52 to change interaction partners and act as a multifunctional protein. Acetylation may provide important contributions to this regulation.

### Function of Human RAD52 for RAD51 localization at DSB sites

Feng et al. demonstrated that the depletion of human RAD52 has small effects on decreasing HR and IR-induced RAD51 foci formation, as compared to the depletion of human BRCA2 [11]. They also demonstrated, however, that the depletion of human RAD52 in a BRCA2-depleted background further impairs both HR and RAD51 foci formation [11]. Based on these observations, they proposed that two pathways lead to RAD51-dependent HR: One is a dominant pathway in which BRCA2 mediates the recruitment of RAD51 to DSB sites, and the other is a pathway in which RAD52 alternatively mediates RAD51 recruitment [11]. The latter pathway becomes evident when the dominant BRCA2-mediated pathway is disrupted. Importantly, in the present study, non-acetylatable RAD52 impaired the localization of RAD51 foci at DSB sites, even in the presence of the dominant BRCA2 pathway, suggesting that the non-acetylatable RAD52 may competitively interfere with the BRCA2-mediated pathway. With regard to the reduced co-localization of RAD51 with γH2AX, the acetylation-defective RAD52 10xR mutant may have a dominant-negative effect by binding to RAD51 and preventing the localization of RAD51 at DSB sites. In the presently determined kinetics of RAD51 foci, the RAD52 10xR mutant does not inhibit the initial step of RAD51 recruitment to DSB sites in the presence of BRCA2. We suppose that RAD51 transiently accumulates and interacts with RAD52 at DSB sites; however, RAD51 might subsequently dissociate from the DSB sites along with RAD52 in this mutant. Here we have described the novel post-translational acetylation of RAD52, and demonstrated that the failure to acetylate RAD52 critically impacts HR. Our results suggest that an inhibitor of RAD52 acetylation could be exploited for anticancer therapy.

### Crosstalk between acetylation and phosphorylation at DSB sites

Our results demonstrated that DSB-induced RAD52 acetylation requires ATM kinase (Fig 20). We also found that inhibiting the function of ATM caused a decrease in the accumulation of RAD52 and p300/CBP at DSB sites. From these results, we drew the following hypothesis: In the absence of DSBs, p300, CBP, and RAD52 are diffused in the nucleus. After DSB-induction, these proteins accumulate at DSB sites, which will promote the interaction between RAD52 and p300 or CBP, thereby inducing the acetylation of RAD52. Accordingly, in the absence of functional ATM, the interaction of RAD52 with p300 and CBP will not be promoted, leading to the loss of acetylated RAD52.

Our findings revealed that the accumulation of SIRT2 and SIRT3 at DSB sites is also triggered by ATM. Similar results were reported for the other HDAC, SIRT1, with ATM being required for its accumulation at DSB sites [49]. Human RAD52 is phosphorylated by c-ABL, and this phosphorylation is required for the IR-induced foci formation of RAD52 [29]. The activation of c-ABL is induced in response to various types of DNA damage, including DSB, and the activation depends on ATM. Therefore, ATM might be required for the IR-induced foci formation of RAD52 at DSB sites through the c-ABL-mediated phosphorylation of RAD52. ATM functions in the DNA damage-induced phosphorylation of p300 [41]. Therefore, we examined whether ATM-mediated phosphorylation is required for the accumulation of p300 at DSB sites, and concluded that p300 phosphorylation is dispensable for its accumulation at DSB sites. To our knowledge, there are no reports that these HATs and HDACs possess DNA binding activity. Therefore, it is unclear how they accumulate near DSB sites. One possibility is that they are recruited to DSB sites via interactions with a protein that exists at DSB sites. ATM phosphorylates several proteins at DSB sites. Taken together, our results suggest that such an ATM-phosphorylated protein at DSB sites interacts specifically with several HATs and HDACs, thereby recruiting them to DSB sites. In contrast to the requirement for ATM in the accumulation of several HATs and HDACs at DSB sites, SIRT1 is required for the accumulation of ATM at DSB sites [49]. Furthermore, ATM is also acetylated by another HAT, Tip60 [50]. ATM acetylation enhances its activation as a protein kinase in response to DNA damage. Thus, there is interplay between acetylation and phosphorylation in response to DNA damage.

## Conclusion

The roles of HATs and HDACs are important topics in many fields of biology and medicine. In this paper, we found the novel roles of HATs (p300/CBP) and HDACs (SIRT2 and SIRT3) in the HR process through the acetylation of human RAD52, indicating that human RAD52 is required for HR, depending on its acetylation status. We further demonstrated that ATM protein kinase is required for DSB-induced RAD52 acetylation, as well as for the accumulation of RAD52, p300/CBP, SIRT2, and SIRT3 at DSB sites. These findings indicate the presence of crosstalk between acetylation and phosphorylation. Therefore, the ATM kinase activation/RAD52 acetylation axis may be important for HR repair.

At DSB sites, several HATs and HDACs regulate histone acetylation, which is required for DNA repair. In addition to histone acetylation, we have demonstrated that HATs (p300/CBP) and HDACs (SIRT2 and SIRT3) directly regulate the acetylation of the non-histone protein, RAD52. We speculate that HATs and HDACs target more DSB repair proteins at DSB sites. These findings provide important information for future studies using *in vitro* reconstitution systems in the context of chromatin, to clarify the molecular mechanisms of DSB repair.

## Materials and methods

### Sister chromatid exchange assay

Confluent cells were exposed to X-rays at a dose rate of 1 Gy/min at room temperature. The cells were immediately subcultured in media with bromodeoxyuridine (3 μg/ml). After adding colcemid for 6 h, the cells were harvested and treated with a hypotonic KCl solution (75 mM) at 37°C for 20 min, followed by methanol:acetic acid (3:1) fixation. After three rounds of fixation, the cells were dropped onto slides. The slides were treated with Hoechst 33258 (10 μg/ml) for 20 min and exposed to a black light for 30 min at 55°C. Finally, the slides were treated with 2XSSC (saline sodium citrate) solution for 20 min at 65°C. The slides were stained using 5% filtered Giemsa solution mixed in Gurr. Images of metaphase cells were obtained using a Zeiss Axioplan microscope (Carl Zeiss) equipped with a QImaging Exi Aqua Cooled CCD camera (QImaging, Surrey, Canada). Sister chromatid exchanges were counted per chromosome.

## Supporting information

S1 Text Supporting Materials and Methods and Supporting References. (PDF)

S1 Fig Related to Fig 1. ***In vitro* acetylation of human RAD52 by p300/CBP.** (A) Physical interaction of human RAD52 with p300. The RAD52 or GST protein was incubated with or without FLAG-p300 in buffer P, and a pull-down assay was performed as described in the Supporting Materials and Methods. Input or immunoprecipitated (IP) proteins were detected by a mixture of anti-RAD52 and anti-GST antibodies (top) or an anti-FLAG (M5) antibody (bottom). (B, C, D, F) *In vitro* acetylation assays were performed as described in the Supporting Materials and Methods, using HAT buffer A containing sodium butyrate. Where indicated, [^14^C]Ac-CoA was added. The reactions were analyzed by Coomassie Brilliant Blue staining (left) or autoradiography (right). (B) RAD52 (3 μg), DNA polymerase β (3 μg), or BSA (3 μg) was incubated with FLAG-p300 (2 μg) where indicated. (C, D) RAD52 (1.5 μg), RAD51 (1.5 μg), or DNA polymerase κ (1.5 μg) was incubated with 1 μg of CBP-FLAG (C) or FLAG-p300 (D). (E) Silver staining of the RAD52, RAD51, DNA polymerase κ, FLAG-p300 and CBP-FLAG proteins used in S1C and S1D Fig. (F) RAD52 (FL, 2 μg), RAD52 (N, 2 μg), or RAD52 (C, 2 μg) was incubated with FLAG-p300 (1 μg), as indicated.
(PDF)

S2 Fig Amino acid sequence alignment of RAD52 proteins. Alignment of RAD52 proteins from *Mus musculus* (NCBI accession number AAA85794), *Rattus norvegicus* (NCBI accession number NP_001100087), *Cricetulus griseus* (NCBI accession number NP_001233693), *Homo sapiens* (NCBI accession number AAA85793), *Pan troglodytes* (NCBI accession number JAA24777), Rhesus monkey (NCBI accession number AFH33435), *Gallus gallus* (NCBI accession number NP_001161231), and *Xenopus laevis* (NCBI accession number NP_001089585), was performed using the Clustal 2.1 multiple sequence alignment program.
(PDF)

S3 Fig Related to Fig 2. **Schematic representation of RAD52 wild-type and acetylation-site mutants used in this study.** Mutations were introduced in functional domains, such as the highly conserved region (K133R, K133/K177R), the RPA binding region (K262R), and the RAD51 binding region (K323R), and also introduced outside the domains (190/192R). The 13xR and 11xR mutants contain multiple mutations including the NLS sequence, whereas the acetylation sites in the NLS sequence are normal in the 10xR and 8xR mutants. The NLS sequence is conjugated at the N-terminal in NLS-RAD52 (Wt) and NLS-RAD52 (13xR). The 10xQ mutant contains multiple glutamine (Q) substitutions at the same mutated sites as in the 10xR mutant.
(PDF)

S4 Fig Related to Fig 2. **ssDNA binding activity of the RAD52 11xR mutant.** (A) Electrophoretic mobility shift assay (EMSA) was performed using a 50-mer oligonucleotide (10 μM in nucleotides) with a Cy5 dye attached to the 5' end (oligo 1), and the indicated concentrations of RAD52 or the RAD52 11xR mutant. (B) Percentages of ssDNA bound by RAD52 (open circles, blue) and the RAD52 11xR mutant (open triangles, green) as a function of the protein concentration.
(PDF)

S5 Fig Related to Fig 3. **Human RAD52 is acetylated by p300/CBP *in vivo*.** (A) Acetylation of the FLAG-RAD52-HA protein purified from T-Rex-293 (HEK293) cells was detected as described in the Supporting Materials and Methods, using the indicated antibodies. pRc/RSV-CBP-HA was transfected into cells, and FLAG-RAD52-HA was purified from cell extracts 24 h after transfection. (B, C) MRC5V1 cells were transfected with the expression plasmid for HA-p300 (B) or CBP-HA (C), and were unirradiated or irradiated with γ-rays (8 Gy) at 24 h after the transfection. At the indicated time after irradiation, the cells were subjected to immunofluorescent staining with an anti-HA (green) antibody, an anti-γH2AX (red) antibody, and 4',6-diamidino-2-phenylindole (DAPI, blue). (D, E) T-Rex-293 cells expressing Myc-RAD52 were transfected with the expression plasmid for HA-p300 (D) or CBP-HA (E), and were irradiated with γ-rays (8 Gy) at 24 h after the transfection. At the indicated time after irradiation, the cells were subjected to immunofluorescent staining with anti-HA (green) and anti-Myc (red) antibodies. (F, G) DSP-mediated cross-linking experiments were performed in the presence or absence of doxorubicin, as described in the Supporting Materials and Methods. RAD52-HA or GST-HA was expressed, as indicated. Whole cell extracts (WCE; left panel) or immunoprecipitates (IP; right panel) with anti-p300 (F) or anti-CBP (G) antibodies. (H, I) T-Rex-293 cells expressing FLAG-RAD52-HA were transfected with either a negative control siRNA or mixture of p300 and CBP-specific siRNAs. Cell extracts were subjected to immunoblotting analyses with the indicated antibodies. (A, H, I) The relative band intensities normalized to those of the HA or GAPDH bands are shown below the immunoblots.
(PDF)

S6 Fig Related to Figs 3 and 7. **Immunostaining of empty expression vector containing cells with acetylated RAD52 antibody.** T-Rex-293 cells containing the empty pT-Rex-DEST30 vector were irradiated with γ-rays (8 Gy). At 1 hour after irradiation, the cells were subjected to immunofluorescent staining with an anti-γH2AX antibody (red), an anti-acetylated RAD52 at K323 antibody (blue or white), and DAPI (blue).
(PDF)

S7 Fig Related to Figs 7, 15, 17, and 18. **Confirmation of siRNA-mediated knockdown.** T-Rex-293 (A, B), HEK293 (C, E) or HeLa pDR-GFP (D, F, G) cells were transfected with the negative control (shown by “C”) or the indicated siRNA, as described in the Supporting Materials and Methods. The mixture of siRAD52 (CDS#1, CDS #2 and CDS #3), the mixture of siRAD51 (#1 and #2), and the mixture of siBRCA2 (#1, #2 and #3) were used in panels D, F, and G, respectively. Whole cell extracts (A, B, C, E and F) or total RNA from the cells (D and G) were prepared 72 h after siRNA transfection, and were subjected to immunoblotting (A, B, C, E and F) and RT-PCR (D and G) analyses, respectively.
(PDF)

S8 Fig Related to Figs 11, 12 and 13. **ATM inhibition does not affect cellular protein levels of RAD52, p300, CBP, SIRT2 and SIRT3**. (A-E) Cell extracts prepared under the same experimental conditions as in Figs 11E, 12A, 12B, 13A and 13B were subjected to immunoblotting analyses, using the indicated antibodies. The relative band intensities normalized to those of the GAPDH bands are shown in the graphs. The graphs show the mean values and standard errors of the mean from 3–7 independent experiments (N.S., not significant by t-test).
(PDF)

S9 Fig Related to Figs 15 and 18. **CRISPR-Cas9-mediated genome editing.** (A) The donor plasmid DNA used for the CRISPR-Cas9-mediated knock-in of FLAG and the HA-tagged RAD52 coding sequence (CDS) into the genomic region of the targeted RAD52 gene. Multisite Gateway technology was used for the construction of the donor plasmid DNA, as described in the Supporting Materials and Methods. (B, C, D, E) PCR analysis of knock-in HeLa pDR-GFP and HEK293 cells. (B, C) The DNA sequences of the forward (P1) and reverse (P2 and P3) primers used in the PCR analysis are described in the Supporting Materials and Methods. The primers P1, P2 and P3 anneal the genomic DNA region upstream from the start codon of the RAD52 gene, the SV40 polyA region from pT-Rex-DEST30, and the intron region of RAD52, respectively. (D, E) Agarose gel electrophoresis of the PCR products. The genomic DNAs purified from HeLa pDR-GFP (D) and HEK293 (E) knock-in cells were analyzed by PCR with the indicated primers. As controls, genomic DNAs from untargeted cells were used in lane C. (F, G) Whole cell extracts of the HeLa pDR-GFP (F) and HEK293 (G) knock-in cells were subjected to immunoblotting analyses with the indicated antibodies.
(PDF)

S10 Fig Related to Fig 9. **Effect of acetylation-deficient mutations on ionizing radiation-induced foci formation of RAD52 expressed by the native promoter.** (A) RAD52 (Wt or 10xR) knock-in HeLa pDR-GFP cells, as shown in S9 Fig, were used. The cells expressing HA-tagged RAD52 proteins by the native promoter were treated with siRAD52 (3'UTR #1) in order to deplete the untagged endogenous RAD52. Two days after the siRNA treatment, the cells were unirradiated or irradiated with γ-rays (8 Gy). At the indicated time after irradiation, the cells were subjected to immunofluorescent staining with an anti-HA (green) antibody, an anti-γH2AX (red) antibody, and DAPI (blue). (B) The percentages of RAD52 foci colocalized with γH2AX at 6h after irradiation were calculated, as described in the Supporting Materials and Methods. Error bars indicate the standard error of the mean. Asterisks indicate statistically significant difference between Wt and 10xR (***, *p*<0.001 by t-test).
(PDF)

S11 Fig Related to Fig 18. **Comparison of knockdown effects on HR repair between RAD52 and BRCA2.** HeLa pDR-GFP cells were transfected with the control or indicated siRNA, and were subjected to the DR-GFP assay as described in the Supporting Materials and Methods. In the column labeled siBRCA2 (mix), the mixture of siBRCA2 (#1, #2 and #3) was used. For RAD52 depletion, siRAD52 (CDS #1) was used. Error bars indicate the standard error of the mean from three samples. Asterisks indicate statistically significant differences of each protein-depleted sample, as compared with the control (+I-SceI) (**, *P*<0.01 by t-test). The samples connected by lines were also compared.
(PDF)

S12 Fig Related to Fig 19. **Interactions of human RAD52 with human RAD52, RAD51 and RPA subunits by a yeast two-hybrid analysis.** Yeast MAV203 cells were transformed with expression vectors encoding the indicated NLS-GAL4-activation domain (AD)- and GAL4-DNA binding domain (DBD)-fused proteins. As controls, the empty vector for the expression of the GAL4-DBD fusion protein was used (1–5). The negative control (C1) and positive controls (C2 and C3) were also included. Each transformant was examined for growth on an SC-Leu-Trp agar plate and an SC-Leu-Trp-His agar plate containing 25 mM 3-Amino-1,2,4-Triazole (3AT).
(PDF)

S13 Fig ssDNA binding activity of *in vitro* acetylated RAD52. (A) EMSA was performed using a 50-mer oligonucleotide (10 μM in nucleotides) with a Cy5 dye attached to the 5' end (oligo 1), and the indicated concentrations of RAD52 or acetylated RAD52. (B) Quantification of (A). Percentage of ssDNA bound by RAD52 (open circles, blue) and acetylated RAD52 (open squares, red) as a function of protein concentration. Error bars indicate standard deviation (n = 3).
(PDF)

S1 Table Mascot search results of tryptic-peptide fragment of acetylated RAD52 (FL). (PDF)

S2 Table Mascot search results of Asp-N peptide fragment of acetylated RAD52 (FL). (PDF)

S3 Table Mascot search results of peptide fragment of acetylated RAD52 (N). (PDF)

S4 Table Mascot search results of peptide fragment of acetylated RAD52 (C). (PDF)

## References

[1] Goodarzi AA, Jeggo PA. The repair and signaling responses to DNA double-strand breaks. Adv Genet. 2013;82:1–45. doi: 10.1016/B978-0-12-407676-1.00001-9 . 23721719 10.1016/B978-0-12-407676-1.00001-9

[2] San Filippo J, Sung P, Klein H. Mechanism of eukaryotic homologous recombination. Annu Rev Biochem. 2008;77:229–257. doi: 10.1146/annurev.biochem.77.061306.125255 PMID: WOS:000257596800011. 18275380 10.1146/annurev.biochem.77.061306.125255

[3] Nimonkar AV, Ozsoy AZ, Genschel J, Modrich P, Kowalczykowski SC. Human exonuclease 1 and BLM helicase interact to resect DNA and initiate DNA repair. Proc Natl Acad Sci USA. 2008;105(44):16906–16911. doi: 10.1073/pnas.0809380105 PMID: WOS:000260913800022. 18971343 10.1073/pnas.0809380105 PMC2579351

[4] Sartori AA, Lukas C, Coates J, Mistrik M, Fu S, Bartek J, et al Human CtIP promotes DNA end resection. Nature. 2007;450(7169):509–U6. doi: 10.1038/nature06337 PMID: WOS:000251158500038. 17965729 10.1038/nature06337 PMC2409435

[5] Symington LS, Gautier J. Double-strand break end resection and repair pathway choice. Annu Rev Genet. 2011;45:247–271. doi: 10.1146/annurev-genet-110410-132435 PMID: WOS:000299299600012. 21910633 10.1146/annurev-genet-110410-132435

[6] Shinohara A, Ogawa T. Stimulation by Rad52 of yeast Rad51-mediated recombination. Nature. 1998;391(6665):404–407. doi: 10.1038/34943 PMID: WOS:000071604200059. 9450759 10.1038/34943

[7] Rijkers T, Van Den Ouweland J, Morolli B, Rolink AG, Baarends WM, et al Targeted inactivation of mouse RAD52 reduces homologous recombination but not resistance to ionizing radiation. Mol Cell Biol. 1998;18(11):6423–6429. Epub 1998/10/17. ; PubMed Central PMCID: PMCPMC109228. 9774658 10.1128/mcb.18.11.6423 PMC109228

[8] Liu J, Heyer WD. Who's who in human recombination: BRCA2 and RAD52. Proc Natl Acad Sci U S A. 2011;108(2):441–442. Epub 2010/12/30. doi: 10.1073/pnas.1016614108 ; PubMed Central PMCID: PMCPMC3021045. 21189297 10.1073/pnas.1016614108 PMC3021045

[9] Jensen RB, Carreira A, Kowalczykowski SC. Purified human BRCA2 stimulates RAD51-mediated recombination. Nature. 2010;467(7316):678–683. doi: 10.1038/nature09399 ; PubMed Central PMCID: PMCPMC2952063. 20729832 10.1038/nature09399 PMC2952063

[10] Liu J, Doty T, Gibson B, Heyer WD. Human BRCA2 protein promotes RAD51 filament formation on RPA-covered single-stranded DNA. Nat Struct Mol Biol. 2010;17(10):1260–1262. doi: 10.1038/nsmb.1904 PMID: WOS:000282563600017. 20729859 10.1038/nsmb.1904 PMC2952495

[11] Feng ZH, Scott SP, Bussen W, Sharma GG, Guo GS, Pandita TK, et al Rad52 inactivation is synthetically lethal with BRCA2 deficiency. Proc Natl Acad Sci USA. 2011;108(2):686–91. doi: 10.1073/pnas.1010959107 PMID: WOS:000286097700049. 21148102 10.1073/pnas.1010959107 PMC3021033

[12] Mortensen UH, Bendixen C, Sunjevaric I, Rothstein R. DNA strand annealing is promoted by the yeast Rad52 protein. Proc Natl Acad Sci USA. 1996;93(20):10729–10734. doi: 10.1073/pnas.93.20.10729 PMID: WOS:A1996VL33300038. 8855248 10.1073/pnas.93.20.10729 PMC38223

[13] Brouwer I, Zhang H, Candelli A, Normanno D, Peterman EJG, et al Human RAD52 Captures and Holds DNA Strands, Increases DNA Flexibility, and Prevents Melting of Duplex DNA: Implications for DNA Recombination. Cell Rep. 2017;18(12):2845–2853. Epub 2017/03/23. doi: 10.1016/j.celrep.2017.02.068 ; PubMed Central PMCID: PMCPMC5379009. 28329678 10.1016/j.celrep.2017.02.068 PMC5379009

[14] Hays SL, Firmenich AA, Massey P, Banerjee R, Berg P. Studies of the interaction between Rad52 protein and the yeast single-stranded DNA binding protein RPA. Mol Cell Biol. 1998;18(7):4400–4406. Epub 1998/06/25. ; PubMed Central PMCID: PMCPMC109024. 9632824 10.1128/mcb.18.7.4400 PMC109024

[15] Shen Z, Cloud KG, Chen DJ, Park MS. Specific interactions between the human RAD51 and RAD52 proteins. J Biol Chem. 1996;271(1):148–152. Epub 1996/01/05. . 8550550 10.1074/jbc.271.1.148

[16] Reddy G, Golub EI, Radding CM. Human Rad52 protein promotes single-strand DNA annealing followed by branch migration. Mutat Res-Fund Mol M. 1997;377(1):53–59. doi: 10.1016/S0027-5107(97)00057-2 PMID: WOS:A1997XG85200007. 10.1016/s0027-5107(97)00057-2 9219578

[17] Lao JP, Oh SD, Shinohara M, Shinohara A, Hunter N. Rad52 promotes postinvasion steps of meiotic double-strand-break repair. Mol Cell. 2008;29(4):517–524. doi: 10.1016/j.molcel.2007.12.014 PMID: WOS:000253693600014. 18313389 10.1016/j.molcel.2007.12.014 PMC2351957

[18] Mcllwraith MJ, West SC. DNA repair synthesis facilitates RAD52-mediated second-end capture during DSB repair. Mol Cell. 2008;29(4):510–516. doi: 10.1016/j.molcel.2007.11.037 PMID: WOS:000253693600013. 18313388 10.1016/j.molcel.2007.11.037

[19] Kagawa W, Kurumizaka H, Ikawa S, Yokoyama S, Shibata T. Homologous pairing promoted by the human Rad52 protein. J Biol Chem. 2001;276(37):35201–35208. doi: 10.1074/jbc.M104938200 . 11454867 10.1074/jbc.M104938200

[20] Ranatunga W, Jackson D, Lloyd JA, Forget AL, Knight KL, Borgstahl GEO. Human RAD52 exhibits two modes of self-association. J Biol Chem. 2001;276(19):15876–80. doi: 10.1074/jbc.M011747200 PMID: WOS:000168623100042. 11278978 10.1074/jbc.M011747200

[21] Stasiak AZ, Larquet E, Stasiak A, Muller S, Engel A, Van Dyck E, et al The human Rad52 protein exists as a heptameric ring. Curr Biol. 2000;10(6):337–40. doi: 10.1016/S0960-9822(00)00385-7 PMID: WOS:000088977900021. 10744977 10.1016/s0960-9822(00)00385-7

[22] Kagawa W, Kurumizaka H, Ishitani R, Fukai S, Nureki O, Shibata T, et al Crystal structure of the homologous-pairing domain from the human Rad52 recombinase in the undecameric form. Mol Cell. 2002;10(2):359–71. . 12191481 10.1016/s1097-2765(02)00587-7

[23] Singleton MR, Wentzell LM, Liu YL, West SC, Wigley DB. Structure of the single-strand annealing domain of human RAD52 protein. Proc Natl Acad Sci USA. 2002;99(21):13492–13497. doi: 10.1073/pnas.212449899 PMID: WOS:000178635700029. 12370410 10.1073/pnas.212449899 PMC129701

[24] Kagawa W, Kagawa A, Saito K, Ikawa S, Shibata T, Kurumizaka H, et al Identification of a second DNA binding site in the human Rad52 protein. J Biol Chem. 2008;283(35):24264–73. doi: 10.1074/jbc.M802204200 ; PubMed Central PMCID: PMCPMC3259773. 18593704 10.1074/jbc.M802204200 PMC3259773

[25] Sacher M, Pfander B, Hoege C, Jentsch S. Control of Rad52 recombination activity by double-strand break-induced SUMO modification. Nat Cell Biol. 2006;8(11):1284–U1259. doi: 10.1038/ncb1488 PMID: WOS:000241732400018. 17013376 10.1038/ncb1488

[26] Saito K, Kagawa W, Suzuki T, Suzuki H, Yokoyama S, Saitoh H, et al The putative nuclear localization signal of the human RAD52 protein is a potential sumoylation site. J Biochem. 2010;147(6):833–42. doi: 10.1093/jb/mvq020 PMID: WOS:000278439900007. 20190268 10.1093/jb/mvq020

[27] Choi BH, Chen Y, Dai W. Chromatin PTEN is involved in DNA damage response partly through regulating Rad52 sumoylation. Cell Cycle. 2013;12(21):3442–3447. doi: 10.4161/cc.26465 PMID: WOS:000327381000015. 24047694 10.4161/cc.26465 PMC3895432

[28] Bassi C, Ho J, Srikumar T, Dowling RJ, Gorrini C, Miller SJ, et al Nuclear PTEN controls DNA repair and sensitivity to genotoxic stress. Science. 2013;341(6144):395–9. doi: 10.1126/science.1236188 ; PubMed Central PMCID: PMCPMC5087104. 23888040 10.1126/science.1236188 PMC5087104

[29] Kitao H, Yuan ZA. Regulation of ionizing radiation-induced Rad52 nuclear foci formation by c-Abl-mediated phosphorylation. J Biol Chem. 2002;277(50):48944–48948. doi: 10.1074/jbc.M208151200 PMID: WOS:000179789600126. 12379650 10.1074/jbc.M208151200

[30] Honda M, Okuno Y, Yoo J, Ha T, Spies M. Tyrosine phosphorylation enhances RAD52-mediated annealing by modulating its DNA binding. EMBO J. 2011;30(16):3368–3382. doi: 10.1038/emboj.2011.238 ; PubMed Central PMCID: PMCPMC3160658. 21804533 10.1038/emboj.2011.238 PMC3160658

[31] Mellor J. The dynamics of chromatin remodeling at promoters. Mol Cell. 2005;19(2):147–157. doi: 10.1016/j.molcel.2005.06.023 PMID: WOS:000230766700002. 16039585 10.1016/j.molcel.2005.06.023

[32] Vogelauer M, Rubbi L, Lucas I, Brewer BJ, Grunstein M. Histone acetylation regulates the time of replication origin firing. Mol Cell. 2002;10(5):1223–1233. doi: 10.1016/S1097-2765(02)00702-5 PMID: WOS:000179450600028. 12453428 10.1016/s1097-2765(02)00702-5

[33] Yamada T, Mizuno K, Hirota K, Kon N, Wahls WP, Hartsuiker E, et al Roles of histone acetylation and chromatin remodeling factor in a meiotic recombination hotspot. EMBO J. 2004;23(8):1792–803. doi: 10.1038/sj.emboj.7600138 PMID: WOS:000221499500012. 14988732 10.1038/sj.emboj.7600138 PMC394230

[34] Ogiwara H, Ui A, Otsuka A, Satoh H, Yokomi I, Nakajima S, et al Histone acetylation by CBP and p300 at double-strand break sites facilitates SWI/SNF chromatin remodeling and the recruitment of non-homologous end joining factors. Oncogene. 2011;30(18):2135–46. doi: 10.1038/onc.2010.592; PMID: WOS:000290249600005. 21217779 10.1038/onc.2010.592

[35] Hasan S, El-Andaloussi N, Hardeland U, Hassa PO, Burki C, Imhof R, et al Acetylation regulates the DNA end-trimming activity of DNA polymerase β. Mol Cell. 2002;10(5):1213–22. . 12453427 10.1016/s1097-2765(02)00745-1

[36] Tini M, Benecke A, Evans RM, Chambon P. Association of CBP/p300 acetylase and thymine DNA glycosylase links DNA repair and transcription. Mol Cell. 2002;9(2):265–277. doi: 10.1016/S1097-2765(02)00453-7 PMID: WOS:000173927000011. 11864601 10.1016/s1097-2765(02)00453-7

[37] Kaidi A, Weinert BT, Choudhary C, Jackson SP. Human SIRT6 Promotes DNA End Resection Through CtIP Deacetylation. Science. 2010;329(5997):1348–1353. doi: 10.1126/science.1192049 PMID: WOS:000281657300040. 20829486 10.1126/science.1192049 PMC3276839

[38] Ohashi E, Ogi T, Kusumoto R, Iwai S, Masutani C, Hanaoka F, et al Error-prone bypass of certain DNA lesions by the human DNA polymerase κ. Gene Dev. 2000;14(13):1589–94. PMID: WOS:000088146000004. 10887153 PMC316741

[39] Oliver L, Hue E, Sery Q, Lafargue A, Pecqueur C, Paris F, et al Differentiation-Related Response to DNA Breaks in Human Mesenchymal Stem Cells. Stem Cells. 2013;31(4):800–7. doi: 10.1002/stem.1336 PMID: WOS:000316624300017. 23341263 10.1002/stem.1336

[40] Stiff T, O'Driscoll M, Rief N, Iwabuchi K, Lobrich M, Jeggo PA. ATM and DNA-PK function redundantly to phosphorylate H2AX after exposure to ionizing radiation. Cancer Res. 2004;64(7):2390–6. doi: 10.1158/0008-5472.Can-03-3207 PMID: WOS:000220586500014. 15059890 10.1158/0008-5472.can-03-3207

[41] Jang ER, Choi JD, Jeong G, Lee JS. Phosphorylation of p300 by ATM controls the stability of NBS1. Biochem Biophys Res Commun. 2010;397(4):637–643. doi: 10.1016/j.bbrc.2010.05.060 . 20471956 10.1016/j.bbrc.2010.05.060

[42] Sy SMH, Huen MSY, Chen JJ. PALB2 is an integral component of the BRCA complex required for homologous recombination repair. Proc Natl Acad Sci USA. 2009;106(17):7155–7160. doi: 10.1073/pnas.0811159106 PMID: WOS:000265584500052. 19369211 10.1073/pnas.0811159106 PMC2678481

[43] Pierce AJ, Johnson RD, Thompson LH, Jasin M. XRCC3 promotes homology-directed repair of DNA damage in mammalian cells. Gene Dev. 1999;13(20):2633–2638. doi: 10.1101/gad.13.20.2633 PMID: WOS:000083420900003. 10541549 10.1101/gad.13.20.2633 PMC317094

[44] Nishimura K, Ishiai M, Horikawa K, Fukagawa T, Takata M, et al Mcm8 and Mcm9 form a complex that functions in homologous recombination repair induced by DNA interstrand crosslinks. Mol Cell. 2012;47(4):511–522. Epub 2012/07/10. doi: 10.1016/j.molcel.2012.05.047 . 22771115 10.1016/j.molcel.2012.05.047

[45] Lan L, Ui A, Nakajima S, Hatakeyama K, Hoshi M, Watanabe R, et al The ACF1 complex is required for DNA double-strand break repair in human cells. Mol Cell. 2010;40(6):976–87. doi: 10.1016/j.molcel.2010.12.003 PMID: WOS:000285823900015. 21172662 10.1016/j.molcel.2010.12.003

[46] Stark JM, Pierce AJ, Oh J, Pastink A, Jasin M. Genetic steps of mammalian homologous repair with distinct mutagenic consequences. Mol Cell Biol. 2004;24(21):9305–9316. doi: 10.1128/MCB.24.21.9305-9316.2004 PMID: WOS:000224943300005. 15485900 10.1128/MCB.24.21.9305-9316.2004 PMC522275

[47] Gunn A, Stark JM. I-SceI-based assays to examine distinct repair outcomes of mammalian chromosomal double strand breaks. Methods Mol Biol. 2012;920:379–391. doi: 10.1007/978-1-61779-998-3_27 . 22941618 10.1007/978-1-61779-998-3_27

[48] Wang X, Hayes JJ. Acetylation mimics within individual core histone tail domains indicate distinct roles in regulating the stability of higher-order chromatin structure. Mol Cell Biol. 2008;28(1):227–236. Epub 2007/10/17. doi: 10.1128/MCB.01245-07 ; PubMed Central PMCID: PMCPMC2223275. 17938198 10.1128/MCB.01245-07 PMC2223275

[49] Dobbin MM, Madabhushi R, Pan L, Chen Y, Kim D, Gao J, et al SIRT1 collaborates with ATM and HDAC1 to maintain genomic stability in neurons. Nat Neurosci. 2013;16(8):1008–U54. doi: 10.1038/nn.3460 PMID: WOS:000322323000010. 23852118 10.1038/nn.3460 PMC4758134

[50] Sun YL, Jiang XF, Chen SJ, Fernandes N, Price BD. A role for the Tip60 histone acetyltransferase in the acetylation and activation of ATM. Proc Natl Acad Sci USA. 2005;102(37):13182–13187. doi: 10.1073/pnas.0504211102 PMID: WOS:000231916300033. 16141325 10.1073/pnas.0504211102 PMC1197271

