# Supplementary material for: Novel function of HATs and HDACs in homologous recombination through acetylation of human RAD52 at double-strand break sites
Source: PLoS Genet. 2018 Mar 28;14(3):e1007277. doi: 10.1371/journal.pgen.1007277 (PMC5891081; doi:10.1371/journal.pgen.1007277)
Supplement: S1 Text — (PDF) [file pgen.1007277.s001.pdf]

Supporting Materials and Methods

Plasmids

The sequence encoding N-terminally FLAG and C-terminally hemagglutinin (HA)-tagged human RAD52 was cloned into the pT-Rex-DEST30 (Life Technologies, Carlsbad, CA, USA), plenti6/V5 DEST, pDEST22 or pDEST32 (Life Technologies) vector, using Gateway cloning technology (Life Technologies). The DNA encoding N-terminally Myc and C-terminally V5-tagged human RAD52 was cloned into pT-Rex-DEST30. The empty plasmid of pT-Rex-DEST30 was prepared by removing the site-specific recombination region between the *Eco*RV and *Hpa*I sites. Site-specific mutations were introduced using a Quick-change Site-Directed Mutagenesis Kit (Stratagene, La Jolla, CA, USA). The sequences encoding human RAD52 (full-length) and RAD52 (1-212) were cloned into pET15b (Merck Millipore, Billerica, MA, USA) for purification of the recombinant proteins, as previously described [19,24,26]. The DNA encoding N-terminally His6-tagged RAD52 (209-418) was cloned into the *Nde*I-*Bam*HI sites of pET15b. The DNA encoding C-terminally His6-tagged human RAD52 was cloned into the *Nde*I-*Hin*dIII sites of pET21a (Merck Millipore), as previously described [21]. This vector was used as the template to prepare the expression vector for the RAD52 11xR mutant. The sequence encoding N-terminally FLAG and C-terminally HA-tagged Glutathione S-transferase (GST) was cloned into pT-Rex-DEST30.

Expression plasmids, pCMV-HA-p300 [51] and pRc/RSV-CBP-HA [52,53], were obtained from Dr. D. Livingston (Dana-Farber Cancer Institute and Harvard Medical School, Boston, MA, USA) and Dr. R. Goodman (Oregon Health Sciences University, Portland, OR, USA), respectively, via Dr. K. Yokoyama (RIKEN, Tsukuba, Japan). The *Not*I-*Hin*dIII fragment of pCMV-p300NHA was inserted into the *Not*I-*Hin*dIII sites of pFastBac1, and the resulting plasmid was named pTY169. The pTY169 plasmid was

digested with *Not*I and *Afl*II to remove the HA-tagged N-terminal fragment of human p300. The N-terminal fragment of human p300, containing the *Not*I-site and a FLAG tag at the N-terminal, was amplified by polymerase chain reaction (PCR) with the following primers (forward, 5'-ATAAGAATGCGGCCGCATGGACTACAAGGATGACGATGACAAGGCCGAGAATGTGGTGGAACCG-3'; reverse, 5'-GTCTGAATCTGGAGACCAAGGCCACTG-3'), and digested with *Not*I and *Afl*II. The resulting *Not*I-*Afl*II fragment of FLAG-tagged p300 was inserted into the *Not*I-*Afl*II sites of pTY169, and the resulting plasmid was named pTY179. The pTY179 plasmid was digested with *Not*I (blunt-ended) and *Hin*dIII to isolate the FLAG-tagged p300 fragment. The resulting FLAG-tagged p300 fragment was inserted into the *Bam*HI (blunt-ended)-*Hin*dIII sites of pFastBac1, and the resulting plasmid was named pFastBac1-FLAG-p300. The *Bam*HI fragment containing the full-length mouse cDNA linked to an HA tag at the C-terminus from pRc/RSV-CBP-HA was inserted into the *Bam*HI site of pFastBac1, and the resulting plasmid was named pTY168. The pTY168 plasmid was digested with *Sma*I and *Not*I to remove the HA-tagged C-terminal fragment of CBP. The C-terminal fragment of mouse CBP, containing a *Not*I-site and a FLAG tag at the C-terminal, was amplified by PCR with the following primers (forward, 5'-CCTCAGCACCACCCGGGACTCCTACACAGC-3'; reverse, 5'-ATAGTTTAGCGGCCGCCTACTTGTCATCGTCATCCTTGTAGTCCAAACCCTCCACAAACTTTTC-3'), and digested with *Sma*I and *Not*I. The FLAG-tagged CBP fragment was inserted into the *Sma*I-*Not*I sites of pTY168, and the resulting plasmid was named pFastBac1-CBP-FLAG. The expression plasmid for HA-tagged p300 (Wt), pCMVβ-p300 (Wt), was purchased from Merck Millipore. The expression plasmid for I-SceI, pCMV-NLS-I-SceI [43,45], was obtained from Dr. M. Jasin (Memorial Sloan-Kettering Cancer Center, NYC, USA).

The C-terminally HA-tagged human HDAC3, SIRT2, and SIRT3 were amplified by PCR using a mixture of the cDNA library from Hela Cells (Life Technologies), and were cloned into pT-Rex-DEST30 using Gateway cloning technology via pDONR221 (Life Technologies) as an entry vector. The following PCR primers were used for HDAC3 (forward, 5'-GGGGACAAGTTTGTACAAAAAAGCAGGCTTCACCATGGCCAAGACCGTGGCCTATTTCTACGACCCCGACGT-3'; reverse, 5'-GGGGACCACTTTGTACAAGAAAGCTGGGTCCTAAGCGTAATCTGGAACATCGTATGGGTAAATCTCCACATCGCTTTCCTTGTCATTGTCATGGT-3'), for SIRT2 (forward, 5'-GGGGACAAGTTTGTACAAAAAAGCAGGCTTCACCATGGACTTCCTGCGGAACTTATTCTCCCAGACGCTCAG-3'; reverse, 5'-GGGGACCACTTTGTACAAGAAAGCTGGGTCCTAAGCGTAATCTGGAACATCGTATGGGTACTGGGGTTTCTCCCTCTCTGTTGTCCTGGCCTCGT-3'), and for SIRT3 (forward, 5'-GGGGACAAGTTTGTACAAAAAAGCAGGCTTCACCATGGCGTTCTGGGGTTGGCGCGCCGCGGCAGCCCTCCG-3'; reverse, 5'-GGGGACCACTTTGTACAAGAAAGCTGGGTCCTAAGCGTAATCTGGAACATCGTATGGGTATTTGTCTGGTCCATCAAGCTTCCCAGTTTCCCGCT-3').

The RAD52 genomic DNA region upstream from the start codon was amplified by PCR with the following primers (forward, 5'-GGGGACAAGTTTGTACAAAAAAGCAGGCTGTGGTCATAGCTCACTGCAGCCTCAAACTCCTGAG-3'; reverse, 5'-GGGGACAACTTTTGTATACAAAGTTGTCTTGATTCTGGTTGACCTCTATATAAATAAAAAGC-3'), and cloned into pDONR221 P1-P5r. The RAD52 genomic DNA region downstream from the stop codon was amplified by PCR with the following primers (forward, 5'-

GGGGACAACTTTGTATAATAAAGTTGCTGAGGCTCAGGCCACATAATTGGACTCTGTCACA-3'; reverse, 5'-GGGGACCACTTTGTACAAGAAAGCTGGGTAGCATATACACGAAACACAGGTGAATTCCACGTTCA-3'), and cloned into pDONR221 P3-P2. The genomic DNAs purified from HeLa pDR-GFP or HEK293 cells were used as the templates for PCR amplification of the genomic DNA regions. A Blood & Cell Culture DNA Mini Kit (Qiagen, Hilden, Germany) was used for purification of the genomic DNAs. The DNA region encoding the RAD52 coding sequence and the SV40 polyA signal from pT-Rex-DEST30 containing FLAG-RAD52-HA was amplified by PCR with the following primers (forward, 5'-GGGGACAACTTTGTATACAAAAGTTGACCATGGACTACAAAGACGATGACGACAAGTCTGG-3'; reverse, 5'-GGGGACAACTTTGTATAGAAAAGTTGGGTGACTGTTGGGAAGGGCGATCGGTGCGGGCCTCTTCG-3'), and cloned into pDONR221 P5-P4. The DNA region containing the SV40 promoter, neomycin resistance gene and polyA signal from pT-Rex-DEST30 was amplified by PCR with the following primers (forward, 5'-GGGGACAACTTTTCTATACAAAGTTGGGTGTGGAAAGTCCCCAGGCTCCCCAGCAGGCAGA-3'; reverse, 5'-GGGGACAACTTTATTATACAAAGTTGTGCGGGTGTCGGGGCTGGCTTAACTATGCGGCATCA-3'), and cloned into pDONR221 P4r-P3r. By using these four constructs, the DNA donor plasmid for CRISPR-Cas9 mediated genome editing was generated by MultiSite Gateway Technology (Life Technologies). A GeneArt CRISPR Nuclease Vector with CD4 Enrichment kit (Life Technologies) was used for CRISPR/Cas9-mediated genome editing. The CRISPR Nuclease Vectors for specific targeting around the start codon of the RAD52 genomic DNA were constructed with the following primers (forward strand, 5'- TGCGTGGCGCAGTTGCATGAGTTTT-3'; reverse strand, 5'- TCATGCAACTGCGCCACGCACGGTG-3'), or (forward strand, 5'-

CAACCAGAATCAAGATGTCTGTTTT-3'; reverse strand, 5'-AGACATCTTGATTCTGGTTGCGGTG-3'), respectively, according to the protocol from Life Technologies. The CRISPR Nuclease Vectors for specific targeting around the stop codon of the RAD52 genomic DNA were constructed with the following primers (forward strand, 5'- GCAGGTCTGTCATGAGCACGGTTTT-3'; reverse strand, 5'- CGTGCTCATGACAGACCTGCCGGTG-3'), (forward strand, 5'-GGCCTGAGCCTCAGTTAAGAGTTTT-3'; reverse strand, 5'-TCTTAACTGAGGCTCAGGCCCGGTG-3'), or (forward strand, 5'-AATATGATCCATCTTAACTGGTTTT-3'; reverse strand, 5'-CAGTTAAGATGGATCATATTCGGTG-3'), respectively.

The human RAD51, RPA1, RPA2 and RPA3 genes were cloned into pDEST22 (Life Technologies), using Gateway cloning technology (Life Technologies). The following primers, (forward strand, 5'-GGGGACAAGTTTGTACAAAAAAGCAGGCTTCACCATGGCAATGCAGATGCAGCTTGAAGCAAATGCAGATAC-3'; reverse strand, 5'-GGGGACCACTTTGTACAAGAAAGCTGGGTCCTAGTCTTTGGCATCTCCCACTCCATCTGCATTAATGG-3') for RAD51, (forward strand, 5'-GGGGACAAGTTTGTACAAAAAAGCAGGCTTCACCATGGTCGGCCAACTGAGCGAGGGGGCCATTGCGGCCAT-3'; reverse strand, 5'-GGGGACCACTTTGTACAAGAAAGCTGGGTCCTACATCAATGCACTTCTCCTGATGCTCATGACCAGCC-3') for RPA1, (forward strand, 5'-GGGGACAAGTTTGTACAAAAAAGCAGGCTTCACCATGTGGAACAGTGGATTCGAAAGCTATGGCAGCTC -3'; reverse strand, 5'-GGGGACCACTTTGTACAAGAAAGCTGGGTCCTATTCTGCATCTGTGGATTTAAAATGGTCATCAT-3') for RPA2, (forward strand, 5'-GGGGACAAGTTTGTACAAAAAAGCAGGCTTCACCATGGTGGACATGATGGACTTGCCCAGGTCGCGCAT -3'; reverse strand, 5'-

GGGGACCACTTTGTACAAGAAAGCTGGGTCCTAATCATGTTGCACAATCCCTAAAGGATAAAACT-3') for RPA3, were used, respectively.

Cell culture and transfection assays

Insect cells, Sf9 and High Five cells (Life Technologies) were maintained as described in the product manual (Growth and Maintenance of Insect Cell Lines) from Life Technologies. T-Rex-293 cells (HEK293 derivative; Life Technologies), which express the tetracycline (Tet) repressor protein from pcDNA6/TR (Life Technologies), were maintained in Dulbecco's modified Eagle's medium (DMEM)-high glucose (Life Technologies), containing 5% fetal bovine serum (FBS; Life Technologies), 2 mM L-glutamine, 1% penicillin-streptomycin (PS), and 5 μg/ml blasticidin (Life Technologies). MRC5V1 cells [54] were maintained in DMEM-high glucose supplemented with 10% FBS, and 1% PS. MRC5V1 cells stably expressing the Tet repressor protein (MRC5V1-TR) were generated through transfection of pcDNA6/TR, and maintained in the culture medium for MRC5V1 containing 5 μg/ml blasticidin. T-Rex-293 cell lines or MRC5V1-TR cell lines stably expressing Wt or mutant FLAG-RAD52-HA proteins were generated through transfection of the pT-Rex-DEST30 vector containing FLAG-RAD52-HA, linearized at the *Kpn*I site, and maintained in the culture medium for T-Rex-293 or MRC5V1-TR containing 1.4 mg/ml or 0.5 mg/ml of Geneticin (Life Technologies), respectively. For immunofluorescence microscopy, the sister chromatid exchange assay, and the cell growth and cell survival assay, T-Rex-293 cell lines or MRC5V1-TR cell lines stably expressing FLAG-RAD52-HA proteins were grown without the Tet inducer. The FLAG-RAD52-HA proteins were weakly expressed without induction. T-Rex-293 cell lines stably expressing the SIRT2-HA, SIRT3-HA or HDAC3-HA proteins were generated by transfection with the pT-Rex-DEST30 vector containing SIRT2-HA, SIRT3-HA or HDAC3-HA, respectively, and maintained in the culture medium for T-Rex-293 containing 1.4 mg/ml of Geneticin (Life Technologies).

The SIRT2-HA, SIRT3-HA and HDAC3-HA proteins were weakly expressed without induction. For immunofluorescence microscopy, the cells were grown without the Tet inducer. T-Rex-293 cell lines stably containing the empty vector were generated through transfection of the pT-Rex-DEST30 digested at the *Eco*RV and *Hpa*I sites. FLAG-RAD52-HA was stably expressed in MRC5V1 cells or UE6E7T-11 bone marrow-derived human MSCs (Health Science Research Resources Bank, National Institute of Biomedical Innovation, Osaka, Japan), using the lentiviral vectors plenti6/V5 DEST or plenti6/V5 TOPO, according to the instructions provided with the ViraPower Lentiviral Expression System (Life Technologies). The stably transfected MRC5V1 cells were maintained in DMEM-high glucose, supplemented with 10% FBS, 1% PS, and 5 μg/ml blasticidin. The stably transfected MSCs were maintained in MEM Alpha (Life Technologies), supplemented with 15% FBS, 1% PS, 50 ng/ml basic fibroblast growth factor (ReproCELL, Kanagawa, Japan), and 5 μg/ml blasticidin. The Amaxa Nucleofector System (Lonza, Basel, Switzerland) and the Human MSC Nucleofector kit (Lonza) were used to transfect pT-Rex-DEST30-based plasmids into MSCs, according to the manufacturer's instructions. HeLa pDR-GFP cells [45,46], obtained from Dr. M. Jasin, were maintained in minimum essential medium with Eagle's salts (Life Technologies), supplemented with 10% FBS, 2 mM L-glutamine, and 1% PS. HeLa pDR-GFP cells stably expressing the Wt or 10xR FLAG-RAD52-HA proteins were generated through transfection of the linearized pT-Rex-DEST30 vector containing FLAG-RAD52-HA, and maintained in the culture medium for Hela pDR-GFP containing 0.5 mg/ml of Geneticin. All human cells were grown at 37°C under 5% CO_2.

CRISPR/Cas9-mediated genome editing

A GeneArt CRISPR Nuclease Vector with CD4 Enrichment kit (Life Technologies) was used for the substitution of the genomic RAD52 gene with FLAG-RAD52-HA

from the DNA donor plasmid, constructed by MultiSite Gateway Technology (Life Technologies). Among the constructed CRISPR Nuclease Vector plasmids, the two plasmids for targeting around the start codon or stop codon of the RAD52 genomic DNA were co-transfected into HeLa pDR-GFP or HEK293 cells, to introduce cleavage around both the start and stop codons of the genomic RAD52 gene. The DNA donor plasmid was also co-transfected with the CRISPR Nuclease Vector plasmids. The transfected cells were cultured in medium containing 0.5 mg/ml of Geneticin. Most of the cells died in the presence of Geneticin. The Geneticin-resistant clones were isolated, and the genomic DNA from each clone was purified with a QIAamp DNA Blood Mini Kit (Qiagen). The knock-in of the DNA donor in the targeted endogenous RAD52 gene was examined by a PCR analysis, and was finally verified by DNA sequence analysis. For the PCR analysis in S7 Fig, the following primers were used (P1, 5'-TATATAGAGGTCAACCAGAATCAAG-3'; P2, 5'-GGCCAGTGCCTAGCTTATAATACGACTCACTATAG-3'; P3, 5'-GCTCACACATGTACTATGTAAACGT-3').

Purification of recombinant proteins

Recombinant baculoviruses for the expression of FLAG-p300 and CBP-FLAG were generated using pFastBac1-FLAG-p300 and pFastBac1-CBP-FLAG, respectively, as described in the user manual of the Bac-to-Bac Baculovirus Expression System from Life Technologies. High Five cells (typically more than twenty 150-mm dishes) were infected with the recombinant baculoviruses, and the cells were cultured at 27°C for 3 d. The infected cells were then collected by centrifugation. The cell pellet collected from one 150-mm dish was resuspended in 2 ml of ice-cold NP lysis buffer A [20 mM Tris-HCl, 1 mM EDTA, 300 mM NaCl, 10% glycerol, 1% Nonidet P-40 (NP-40), 1 mM phenylmethylsulfonyl fluoride (PMSF), 1 mM benzamine, 1 mM dithiothreitol (DTT), pH 7.5] containing Complete EDTA-free Protease Inhibitor Cocktail (one tablet/50 ml;

Roche Applied Science, Basel, Switzerland) for 30 min. The cell suspension was centrifuged at 14,000 x *g* for 30 min and at 77,000 x *g* for 60 min at 4°C. A 1/20 volume of anti-FLAG M2 agarose affinity gel (Sigma-Aldrich) was added to the cell extract, and incubated for 2 h at 4°C with rotation. The beads were extensively washed with buffer W (50 mM Tris-HCl, 1 mM EDTA, 300 mM NaCl, 10% glycerol, 1 mM PMSF, 1 mM benzamine, pH 7.5) containing Complete EDTA-free Protease Inhibitor Cocktail, and the bound proteins were eluted with buffer A (same volume as that of the anti-FLAG M2 agarose affinity gel) containing 500 ng/ml FLAG peptide (Sigma-Aldrich). The eluted solution was concentrated with an Amicon Ultra-15 10,000 molecular weight cut-off filter (Merck Millipore), and loaded onto a Sephacryl S-300 16/60 column (GE Healthcare, Buckinghamshire, UK) equilibrated with buffer S (50 mM Tris-HCl, 1 mM EDTA, 300 mM NaCl, 1 mM DTT, pH 7.5) containing 10% glycerol. The fractions containing FLAG-p300 or CBP-FLAG were dialyzed against buffer S containing 50% glycerol, and stored at -80°C.

Recombinant human RAD52 (full length; FL), RAD51, and RAD52 (1-212; N) were overexpressed in *E. coli* and purified, as previously described [19,22]. RAD52 (209-418; C) was purified in a three-step procedure involving nickel-nitrilotriacetic acid (Ni-NTA) agarose purification, removal of the His6-tag, and Affigel Blue (Bio-Rad, Hercules, CA, USA) column chromatography. RAD52 (209-418) was expressed in the *E. coli* strain JM109 (DE3), containing both pET15b-RAD52 (209-418) and an expression vector for *E. coli* $tRNA^{Arg3}$ and $tRNA^{Arg4}$. Bacteria were grown at 30°C in lysogeny broth containing ampicillin (100 μg/ml) and chloramphenicol (35 μg/ml) to an optical density of 0.7 at 600 nm, and protein production was induced at 18°C with 0.5 mM isopropyl-β-D-thiogalactopyranoside overnight. The collected cells were resuspended in lysis buffer [50 mM Tris-HCl, 1.0 mM KCl, 5 mM imidazole, 10% glycerol, 5 mM 2-mercaptoethanol (2-ME), pH 7.8] containing Complete EDTA-free Protease Inhibitor Cocktail on ice. After cell disruption by ultrasonication, the cell

suspension was centrifuged at 27,700 x *g* for 30 min at 4°C. The supernatant was mixed by a batch method with 2.5 ml Ni-NTA agarose beads (Qiagen) for 1 h at 4°C. The Ni-NTA agarose beads were then packed into an Econo-column (Bio-Rad) and washed with washing buffer (50 mM Tris-HCl, 1.0 mM KCl, 50 mM imidazole, 10% glycerol, 5 mM 2-ME, pH 7.8). The retained proteins were eluted with a 10 to 300 mM imidazole gradient. To remove the His6-tag from the RAD52 (209-418), 3 units of thrombin protease (GE Healthcare) per milligram of protein were added to the peak fractions containing RAD52 (209-418), and the collected sample was dialyzed against dialysis buffer (20 mM HEPES-KOH, 200 mM KCl, 0.5 mM EDTA, 10% glycerol, 2 mM 2-ME, pH 7.5). The sample was then loaded onto the Affigel Blue column (3 ml) equilibrated with dialysis buffer. After washing with dialysis buffer, the retained proteins were eluted with a 0.2 to 1.5 M KCl gradient in dialysis buffer. The eluted fractions containing RAD52 (209-418) were pooled and concentrated with an Amicon Ultra-15 10,000 molecular weight cut-off filter (Merck Millipore). Full-length human RAD52 purchased from Bio Academia (Osaka, Japan) was also used (Fig 1B and 1C).

RAD52 11xR, a full-length RAD52 mutant in which 11 of the 13 identified acetylation sites were substituted with arginine, was expressed in the *E. coli* JM109(DE3) strain as a C-terminally hexahistidine-tagged protein. RAD52 11xR was purified as previously described [22], with the exception of using SP Sepharose instead of Heparin Sepharose.

The human RPA protein complex was purified, as described [55]. The GST protein was expressed with pGEX-6P-1 (GE Healthcare) and was purified with GSTrap FF (GE Healthcare), according to the manufacturer's instructions. DNA polymerase β and DNA polymerase κ were purchased from Bio Academia.

siRNA treatments

Stealth Select siRNAs and Stealth siRNAs were purchased from Life Technologies. The Stealth Select siRNA, HSS117928 (siSIRT2 (#1); 5'-

AAUAAGUUCCGCAGGAAGUCCAUGU-3'), HSS177042 (siSIRT2 (#2); 5'-AAUAGCUGAUCUCAAAGAUGGCCUC-3'), or HSS177043 (siSIRT2 (#3); 5'-GAGAGCCUCCCAGCGCGUUUCUUCU-3') (S7 Fig), or a mixture of the Stealth Select siRNAs (Fig 7) was used for the siRNA treatment against SIRT2. The Stealth Select siRNA, HSS118726 (siSIRT3 #1; 5'-AAUCAGCUCAGCUACAUCCUGCAGG-3'), HSS118728 (siSIRT3 #2; 5'-GCUGGUUGAAGCUCAUGGAACCUUU -3'), or HSS177402 (siSIRT3 #3; 5'-ACCACAUGCAGCAAGAACCUCUGGG -3') (S7 Fig), or a mixture of the Stealth Select siRNAs (Fig 7) was used for the siRNA treatment against SIRT3. A mixture of Stealth Select siRNAs, HSS181472 (5'-AGUCUAGUACUUAAUGAUCUGCUUA-3') and HSS181473 (5'-CCUGAUUCGAGAUCCUGAAACAAUU-3'), was used for siRNA treatment against ATM. Stealth Select siRNAs, HSS109012 (siRAD51 (#1); 5'-UCAAGUGGAUGGAGCAGCGAUGUUU-3') or HSS109013 (siRAD51 (#2); 5'-GAAGCUGAAGCUAUGUUCGCCAUUA-3'), or a mixture of the Stealth Select siRNAs (S7F Fig) was used for the siRNA treatment against RAD51. Stealth Select siRNAs, HSS109021 (siRAD52 (CDS #1); 5'-GGCCAAUGAGAUGUUUGGUUACAAU-3'), HSS109023 (siRAD52 (CDS #2); 5'-GAUACAACAGCUGCCGACCGAACAU-3') or HSS184195 (siRAD52 (CDS #3); 5'-CAGCACUCCUGUAACUGUCUCAGAA-3'), or a mixture of the Stealth Select siRNAs (S7D Fig) was used for the siRNA treatment against the coding sequence (CDS) of RAD52. The Stealth siRNA, U27516_stealth_2532 (siRAD52 (3'UTR #1); 5'-CAGGUGUGAGAUGUAACCCACCUUG -3') or U27516_stealth_2539 (siRAD52 (3'UTR #2); 5'- GAGAUGUAACCCACCUUGACCAUAA -3') was used for the siRNA treatment against the 3'UTR region of RAD52. Stealth Select siRNAs, HSS101097 (siBRCA2 (#1); 5'-GAGCGCAAAUAUAUCUGAAACUUCU-3'), HSS101095 (siBRCA2 (#2); 5'-GGAACCAAAUGAUACUGAUCCAUUA-3') or HSS186121 (siBRCA2 (#3); 5'-CAUAUUGCAGAAGAGUACAUUUGAA-3'), or a

mixture of the Stealth Select siRNAs (S7G Fig; S10 Fig) was used for the siRNA treatment against BRCA2. A mixture of Stealth Select siRNAs, HSS103258 (5'-CCUGCCCGGUGAACUCUCCUAUAAU-3'), HSS103259 (5'-GGAUUCGUCUGUGAUGGCUGUUUAA-3') and HSS176564 (5'-CAGGUAUGAUGAACAGUCCAGUAAA-3'), was used for siRNA treatment against p300. A mixture of Stealth Select siRNAs, HSS102269 (5'-CCAGUGCCAAGGAACUGCCCUAUUU-3'), HSS102270 (5'-GGCGCAAGUCAUGAAUGGAUCUCUU-3') and HSS175174 (5'-CAGACCCACCCAGGCCUCCUCAAUA-3'), was used for siRNA treatment against CBP. Stealth RNAi negative control siRNA oligonucleotides (Life Technologies) were used for the negative controls. For siRNA treatments in T-Rex-293 and Hela pDR-GFP cells, Lipofectamine 2000 and Lipofectamine RNAiMAX were used for the transfection of Stealth Select siRNAs into cells, respectively, according to the transfection protocol from Life Technologies. In the case of the co-transfection of Stealth Select siRNAs and plasmid DNA into MRC5 V1, 20 pmol siRNAs and 2 μg plasmid DNA were mixed with $1x10^6$ cells in 500 μl Amaxa solutionV (Lonza). Program T-030 of the Amaxa Nucleofector System (Lonza) was used for the transfection.

Antibodies

The following antibodies were used: anti-γH2AX clone JBW301 (Merck Millipore), anti-HA clone 3F10 (Roche Applied Science), anti-FLAG clone M5 (Sigma-Aldrich), anti-FLAG clone M2 (Sigma-Aldrich), anti-GST (GE Healthcare), anti-Myc (Cell Signaling, Danvers, MA, USA), anti-acetyl lysine (Cell Signaling), anti-RAD51 (Bio Academia), anti-RAD52 (Cell Signaling), anti-RAD52 (Santa Cruz Biotechnology, Dallas, Texas, USA; in the case of S7C Fig), DNA polymerase β (Bio Academia), anti-RPA70 (Cell Signaling), anti-MRE11 (Cell Signaling), anti-53BP1 (Cell Signaling),

anti-SIRT2 (Abcam, Cambridge, UK; in the case of Fig 7), anti-SIRT2 (Cell Signaling), anti-SIRT3 (Cell Signaling), anti-HDAC3 (Abnova, Taipei, Taiwan), anti-CHK2 (Cell Signaling), anti-phospho-CHK2 (Thr68) (Cell Signaling), anti-phospho-BRCA1 (Ser1524; Cell Signaling), anti-β-actin (Cell Signaling), anti-GAPDH (Cell Signaling), anti-ATM (Cell Signaling), anti-p300 (Santa Cruz Biotechnology), anti-CBP (Cell Signaling), and anti-γ-tubulin (Sigma-Aldrich). Polyclonal antibodies, anti-Ac-RAD52 (K274) and anti-Ac-RAD52 (K323), were produced by immunizing rabbits with synthetic acetylated lysine-containing peptides, C+RERME(AcK)QQVR and C+TQELI(AcK)TLEDN, where "C+" indicates a cysteine residue required for coupling to the carrier protein, keyhole limpet hemocyanin, and "AcK" indicates an acetylated lysine residue. Antibodies recognizing the acetylated lysine-containing peptide were affinity-purified with the synthetic peptides coupled to resin. Antibodies recognizing the non-acetylated peptide were removed by the non-acetylated peptide coupled to resin. The anti-acetyl RAD52 antibodies were prepared by Scrum Inc. (Tokyo, Japan). Alexa Fluor-labeled secondary antibodies were purchased from Life Technologies. Alkaline phosphatase conjugated secondary antibodies were purchased from Sigma-Aldrich.

Pull-down assay

The human RAD52 (1.2 pmol) or GST (1.2 pmol) protein was mixed with FLAG-p300 (3.7 pmol) or CBP-FLAG (3.7 pmol) in 100 μl of buffer P [20 mM Tris pH 7.5, 0.1 mM EDTA, 0.1 M NaCl, 0.5% Triton-X100, 100 μg/ml BSA], and incubated on ice for 30 min. Subsequently, 10 μl of anti-FLAG M2 agarose (Sigma-Aldrich) was added to each sample mixture, and the mixtures were further incubated at 4°C with rotation. The beads were washed extensively with buffer P, and the bound proteins were eluted using 20 μl buffer P containing 500 ng/ml FLAG peptide (Sigma-Aldrich). The purified protein samples were subjected to SDS-PAGE followed by immunoblotting.

Immunoblotting

For immunoblot analyses, proteins separated by SDS-PAGE were transferred onto Immobilon-P membranes (Merck Millipore) and detected using the Western-Light Plus™ Chemiluminescent Detection system (Tropix, Bedford, MA, USA) with appropriate secondary antibodies. Quantification was performed using an LAS-4000 mini luminoimaging analyzer (Fujifilm, Tokyo, Japan).

***In vitro* acetylation assays**

In vitro acetylation assays were performed by incubating the indicated recombinant proteins in 20 μl of HAT buffer A (50 mM Tris-HCl, 1 mM EDTA, 10% glycerol, 1 mM DTT, pH 8.0) with or without 10 mM sodium butyrate or HAT buffer B (50 mM Tris-HCl, 1 mM $MgCl_2$, 5% glycerol, 1 mM DTT, pH 8.0) at 30°C for 60 min. To detect acetylation by autoradiography, 0.1 μCi [^{14}C] acetyl coenzyme A (Ac-CoA) was added to the reaction mixture where indicated. Linear ssDNA (68 mer of polydT), circular dsDNA (pET44b; Novagene), and linear dsDNA (pET44b cut at the *Sma*I site) were used as indicated. The reactions were subjected to SDS-PAGE analysis. The gels were stained with Coomassie Brilliant Blue, destained, and then incubated in NAMP 100 amplification solution (GE Healthcare) for 30 min. When dry, the gels were photographed using an EOS Kiss digital camera (Cannon, Tokyo, Japan) equipped with an EF 50 mm F1.8 II lens (Cannon), and exposed to Kodak BioMax XAR X-ray film (Sigma-Aldrich). An immunoblotting analysis was also used for the detection of acetylation. In this case, unlabeled Ac-CoA (Sigma-Aldrich) was added to the reaction mixture.

***In vitro* HDAC assays**

In vitro acetylated RAD52 was used as the substrate for *in vitro* HDAC assays. Full-length human RAD52 protein (4 μg) was incubated with CBP (2 μg) and Ac-CoA (4

μg) in HAT buffer A without sodium butyrate, at 30˚C for 30 min. Subsequently, 54.4 nmol (in nucleotides) of poly dT (68 mer; synthesized by Operon Biotechnologies, Japan) was added to the reaction mixture, which was further incubated at 30˚C for 10 min to inhibit the acetylation of Rad52. Subsequently, 1 μl aliquot of the reaction mixture was incubated with each recombinant HDAC protein (0.1 μg) in HDAC buffer [25 mM Tris-HCl, 137 mM NaCl, 2.7 mM KCl, 1 mM $MgCl_2$, 0.1 mg/ml BSA, pH 8.0], at 30˚C for 30 min. In the case of the *in vitro* HDAC assay using the NAD-dependent class III HDAC proteins (SIRT1-7), 500 μM NAD^+ (Sigma-Aldrich) was also added to the reaction mixture. The reaction mixtures were subjected to SDS-PAGE, followed by immunoblotting with an anti-acetylated lysine antibody and an anti-Rad52 antibody. The following recombinant human HDAC proteins were used: HDAC1 (BPS Bioscience, #50001, Lot 81110), HDAC2 (BPS Bioscience, #50002, Lot 81010), HDAC3-NCOR2 (BPS Bioscience, #50003, Lot 80917), HDAC8 (BPS Bioscience, #50008, Lot 81020), HDAC4 (BPS Bioscience, #50004, Lot 2000), HDAC5 (BPS Bioscience, #50045, Lot 80613-2), HDAC6 (BPS Bioscience, #50006, Lot 80926), HDAC7 (BPS Bioscience, #50007, Lot 2002), HDAC9 (BPS Bioscience, #50009, Lot 2000), HDAC10 (BPS Bioscience, #50010, Lot 80902), SIRT1 (BPS Bioscience, #50012, Lot 2001), SIRT2 (BPS Bioscience, #50013, Lot 2000), SIRT3 (BPS Bioscience, #50014, Lot 2003), SIRT4 (BPS Bioscience, #50015, Lot 2007), SIRT5 (BPS Bioscience, #50016, Lot 80903), SIRT6 (BPS Bioscience, #50017, Lot 80902), and SIRT7 (Abnova, # H00051547-P01, Lot 0980305).

Detection of *in vivo* acetylation by immunoblotting

To detect RAD52 acetylation in T-Rex-293 cells, stably containing the pT-Rex-DEST30-FLAG-RAD52-HA vector, with an anti-acetyl lysine antibody, cells in 5x150-mm dishes were cultured in the presence of 1 μg/ml Tet for 24 h to induce the overexpression of the FLAG-RAD52-HA protein. After two washes with phosphate-

buffered saline (PBS), the cells were lysed with 4 ml ice-cold NP lysis buffer B [50 mM Tris-HCl, 1 mM EDTA, 500 mM NaCl, 10% glycerol, 1% NP-40, 0.25 mM PMSF, 10 mM sodium butyrate, and Complete EDTA-free Protease Inhibitor Cocktail (one tablet/50 ml), pH 8.0]. The cell lysates were scraped into centrifuge tubes, and the cell extracts were obtained by centrifugation. A 1/20 volume of anti-FLAG M2 agarose affinity gel was added to the cell extract, and incubated for 2 h at 4°C with rotation. The beads were immediately washed extensively with ice-cold NP lysis buffer B without NP-40. The bound proteins were eluted with buffer E [50 mM Tris-HCl, 1 mM EDTA, 300 mM NaCl, 10% glycerol, 10 mM sodium butyrate, pH 8.0 (same volume as that of the anti-FLAG M2 agarose affinity gel)], containing 500 ng/ml FLAG peptide, and then subjected to 2,2,2-trichloroacetic acid precipitation. The precipitated proteins from the cell extracts in 5x150-mm dishes were resuspended in 50 μl of 8 M urea. Aliquots were subjected to immunoblotting analyses.

To detect acetylated FLAG-RAD52-HA expressed in MSCs using the Amaxa Nucleofector System (Lonza), the cell lysate from one 60-mm dish was used, and acetylation was detected with anti-acetylated RAD52 antibodies. The FLAG-RAD52-HA purification method was essentially the same as that mentioned above, except the trichloroacetic acid precipitation and resuspension in 8 M urea steps were omitted.

Identification of acetylation sites in RAD52

To map the RAD52 acetylation sites, acetylated RAD52 proteins were prepared by an *in vitro* acetylation procedure. Recombinant RAD52 (full length), RAD52 (1-212), and RAD52 (209-418) proteins were used. Each RAD52 protein (40 μg) was incubated with CBP (10 μg) and Ac-CoA (16 μg) in 200 μl of HAT buffer A, containing 10 mM sodium butyrate. The samples were subjected to SDS-PAGE. The protein bands were excised from the gel, subjected to digestion with endoproteinase Asp-N or trypsin, and analyzed by LC-MS using an LCQ Deca XP Plus spectrometer (Finnigan, San Jose, CA,

USA). The acetylated sites were identified with the Mascot software (Matrix Science, Boston, MA, USA).

Immunofluorescence microscopy

Cells plated on glass-bottom dishes were washed with PBS and fixed for 5 min with 1% paraformaldehyde in PBS. After washing with PBS, the cells were permeabilized for 10 min with 0.5% Triton X-100 in PBS. The cells were washed with PBS, blocked with blocking buffer [0.5% skim milk/Tris buffered saline with Tween-20 (TBST)], and incubated with the primary antibody diluted in blocking buffer for 30 min at room temperature. The cells were washed three times with TBST, incubated with Alexa Fluor secondary antibody conjugates (Life Technologies) diluted in blocking buffer for 30 min at room temperature in the dark, and washed three times with TBST. The samples were mounted using Vectashield mounting medium with 4',6-diamidino-2-phenylindole (Vector Laboratories, Burlingame, CA, USA), and visualized using an IX70 fluorescence microscope (Olympus, Tokyo, Japan) equipped with an ORCA-R2 cooled CCD camera (Hamamatsu Photonics, Hamamatsu, Japan) or an Axio Observer microscope (Carl Zeiss, Jena, Germany; in the case of Fig 8C). Deconvolved z-stack images were obtained using the MetaMorph software (Molecular Devices, Sunnyvale, CA, USA) or the Axio Vision software (Carl Zeiss). For the quantitative colocalization analysis of RAD52 or RAD51 foci with γH2AX, the total number of foci (RAD52 or RAD51) and the number of colocalized foci (RAD52 or RAD51) with γH2AX were counted in each cell. For the quantitative colocalization analysis of γH2AX with RAD52, p300, CBP, SIRT2 or SIRT3 foci, the total number of γH2AX foci and the number of the colocalized γH2AX foci were counted in each cell. The percentage of colocalized foci was calculated by dividing the number of the colocalized foci by the total number of foci. For the quantitative analysis of foci formation, the total number of foci in each cell was counted. At least 20 cells were counted for each condition.

DR-GFP assay

Hela pDR-GFP ($5x10^5$ cells/well in a 12-well plate) cells was transfected with siRNA. At 24 h after the siRNA-transfection, the cells were transfected with 1 μg of the I-SceI expression plasmid, pCMV-NLS-I-SceI [38,39,40], and were cultured for 48 h. Subsequently, the cells were harvested by trypsinization and were analyzed with a FACSCaliber (Becton Dickinson, San Jose, CA, USA) or FACSVerse (Becton Dickinson) flow cytometer. GFP-positive cells were counted by using the FlowJo software (Tomy Digital Biology, Tokyo, Japan).

Cross-linking experiments

HEK293 cells cultured in 10 cm dishes were cotransfected with expression plasmids for FLAG-RAD52-HA or FLAG-GST-HA, and expression plasmids for HA-p300 or CBP-HA, and were cultured for 24 h. Subsequently, the cells were harvested and suspended in ice-cold PBS. The cells were then treated with DSP (final concentration of 1.2 mg/ml) as described [49], for 2 h at 4°C and for 15 min at room temperature with rotation. The cross-linking reactions were then stopped by the addition of Tris-HCl (pH 7.5) to a final concentration of 20 mM and incubated for 15 min. The cells were collected by centrifugation, and lysed with 1 ml of ice-cold NP lysis buffer B without sodium butyrate. The whole cell extracts were immunoprecipitated with an anti-p300 or anti-CBP antibody. The samples were treated with DTT to cleave the cross-linker [56], and were subjected to an immunoblotting analysis.

RNA extraction and reverse transcription (RT)-PCR

Total RNA extractions were performed with the TRIzol Reagent (Life Technologies), according to the manufacturer's instructions. The RNA samples were treated with DNase I (Takara Bio Inc. Otsu, Japan) to remove the trace amounts of remaining

genomic DNA. First-strand cDNAs were synthesized from the purified total RNA with the SuperScript III first-strand synthesis system (Life Technologies), according to the manufacturer's instructions. The cDNAs were amplified by PCR, and the PCR products were analyzed by agarose gel electrophoresis. The following PCR primers were used for RAD52 (forward, 5'-GTGTTAGTGAGGGCCTCAAGTCCAA-3'; reverse, 5'-TGGAAGGGGAGGTCACCTGCTGCAG-3'), for BRCA2 (forward, 5'-GCCCAACAAAAGAGACTAGAAGCCT-3'; reverse 5'-TTCAGCAGATTCCATGGCCTTCCTA-3'), and for GAPDH (forward, 5'-ACTGCCAACGTGTCAGTGGTGGACC-3'; reverse, 5'-TTACTCCTTGGAGGCCATGTGGGCC-3').

Yeast two-hybrid analysis

The yeast two-hybrid analysis was performed with a ProQuest Two-Hybrid System (Life Technologies). The plasmid vectors, pDEST22 and pDEST32, were used for the expression of the nuclear localization signal (NLS)- and GAL4 DNA activation domain (AD)-fused proteins, and for the expression of the GAL4 DNA binding domain (DBD)-fused proteins, respectively. As the selection marker, pDEST22 contains the TRP1 gene, and pDEST32 contains the Leu2 gene. Both plasmid constructs with pDEST22 and pDEST32 were co-transformed into competent MAV203 yeast cells, and the transformants were selected on SC-Leu-Trp agar plates, as described in the user manual. Protein-protein interactions were examined on SC-Leu-Trp-His agar plates containing 25 mM 3-Amino-1,2,4-Triazole (3AT). For the quantitative examination of the protein-protein interactions, a liquid β-galactosidase assay with ONPG was performed, as described in the user manual, except that three freeze-thaw cycles with liquid nitrogen were used for the disruption of yeast cells. As positive and negative controls for the protein-protein interaction, the pEXP32/Krev1 and pEXP22/RalGDS (wt, m1 or m2)

plasmids provided in the kit (Life Technologies) were used. At least three samples were counted for each condition.

Cell survival assay

Logarithmically growing cells were plated with various concentrations of cisplatin (Nippon Kayaku, Japan). After 14 days of incubation, the cells were washed with a 0.9% NaCl solution, fixed by 100% ethanol, and stained with 0.1% crystal violet. Colonies containing more than 50 cells were scored as survivors.

Cell growth assay

Cells were seeded in 12-well dishes (50,000 cells/well). The next day (day 0), the cells were treated with the siRNA. Cell growth was examined by counting the numbers of adherent cells with a Z1 Coulter Counter (Beckman Coulter, Brea, CA, USA).

Other DNA damaging treatments

Cells were irradiated at room temperature with γ rays at a dose rate of 8 Gy/min or 1 Gy/min (in the case of Fig 8C), using a ^{137}cesium source. Doxorubicin (2 μg/ml; Wako Pure Chem. Ind., Osaka, Japan) was added to the cell culture to induce DSB production. For the inhibition of ATM, 10 μM of the chemical inhibitor KU55933 (Tocris Bioscience) was added to the cell culture, 1 h before the DNA damaging treatments.

DNA substrates for biochemical assays

For the ssDNA binding assay, a 50-mer ssDNA oligonucleotide (ΦX174-50-1, 5'- TCG CCA TCA ACT AAC GAT TCT GTC AAA AAC TGA CGC GTT GGA TGA GGA GA-3') with a Cy5 fluorophore attached to the 5' end was used. The DNA substrate was

purchased from Fasmac Co. Ltd. (Kanagawa, Japan) as a high pressure liquid chromatography-purified oligonucleotide. The DNA concentration is expressed in moles of nucleotides.

ssDNA binding assay

The reaction mixture (16 μL), containing 1 μL of 50 μM Cy5-labeled ΦX174-50-1 oligonucleotide, 4 μL of 5X reaction buffer (0.1 M HEPES-NaOH, pH 7.5, 10 mM 2-mercaptoethanol), and 1 μL of 10 mg/mL bovine serum albumin (New England Biolabs), was preincubated at 37˚C for 5 min. A 4 μL aliquot of RAD52 was added to the reaction mixture, which was incubated for 5 min. The complexes were fixed with 1 μL of 1% glutaraldehyde (final concentration of 0.05%) at 37˚C for 15 min. The products were fractionated through a 1% Seakem GTG-agarose (Lonza) gel in 1X TAE buffer for 2 hr at 3.3 V/cm. The gels were visualized by monitoring the Cy5 fluorophore, using a Typhoon FLA 7000 imaging analyzer (GE Healthcare).

Supporting References

51. Eckner R, Ewen ME, Newsome D, Gerdes M, DeCaprio JA, Lawrence JB, et al. Molecular cloning and functional analysis of the adenovirus E1A-associated 300-kD protein (p300) reveals a protein with properties of a transcriptional adaptor. Genes Dev. 1994;8(8):869-84. PMID: 7523245.

52. Chrivia JC, Kwok RPS, Lamb N, Hagiwara M, Montminy MR, Goodman RH. Phosphorylated CREB binds specifically to the nuclear protein CBP. Nature. 1993;365(6449):855-9. doi: DOI 10.1038/365855a0. PMID: WOS:A1993MD95100056.

53. Lundblad JR, Kwok RPS, Laurance ME, Harter ML, Goodman RH. Adenoviral E1A-associated protein p300 as a functional homologue of the transcriptional co-activator CBP. Nature. 1995;374(6517):85-88. doi: DOI 10.1038/374085a0. PMID: WOS:A1995QK07900059.

54. Ogi T, Limsirichaikul S, Overmeer RM, Volker M, Takenaka K, Cloney R, et al. Three DNA polymerases, recruited by different mechanisms, carry out NER repair synthesis in human cells. Mol Cell. 2010;37(5):714-27. doi: 10.1016/j.molcel.2010.02.009. PMID: WOS:000275702500015.

55. Henricksen LA, Umbricht CB, Wold MS. Recombinant replication protein A: expression, complex formation, and functional characterization. J Biol Chem. 1994;269(15):11121-11132. PMID: 8157639.

56. Yasuda T, Morimatsu K, Kato R, Usukura J, Takahashi M, Ohmori H. Physical interactions between DinI and RecA nucleoprotein filament for the regulation of SOS mutagenesis. EMBO J. 2001;20(5):1192-202. doi: DOI 10.1093/emboj/20.5.1192. PMID: WOS:000167393900026.
