# Supplementary figures and images for: Novel function of HATs and HDACs in homologous recombination through acetylation of human RAD52 at double-strand break sites
Source: PLoS Genet. 2018 Mar 28;14(3):e1007277. doi: 10.1371/journal.pgen.1007277 (PMC5891081; doi:10.1371/journal.pgen.1007277)

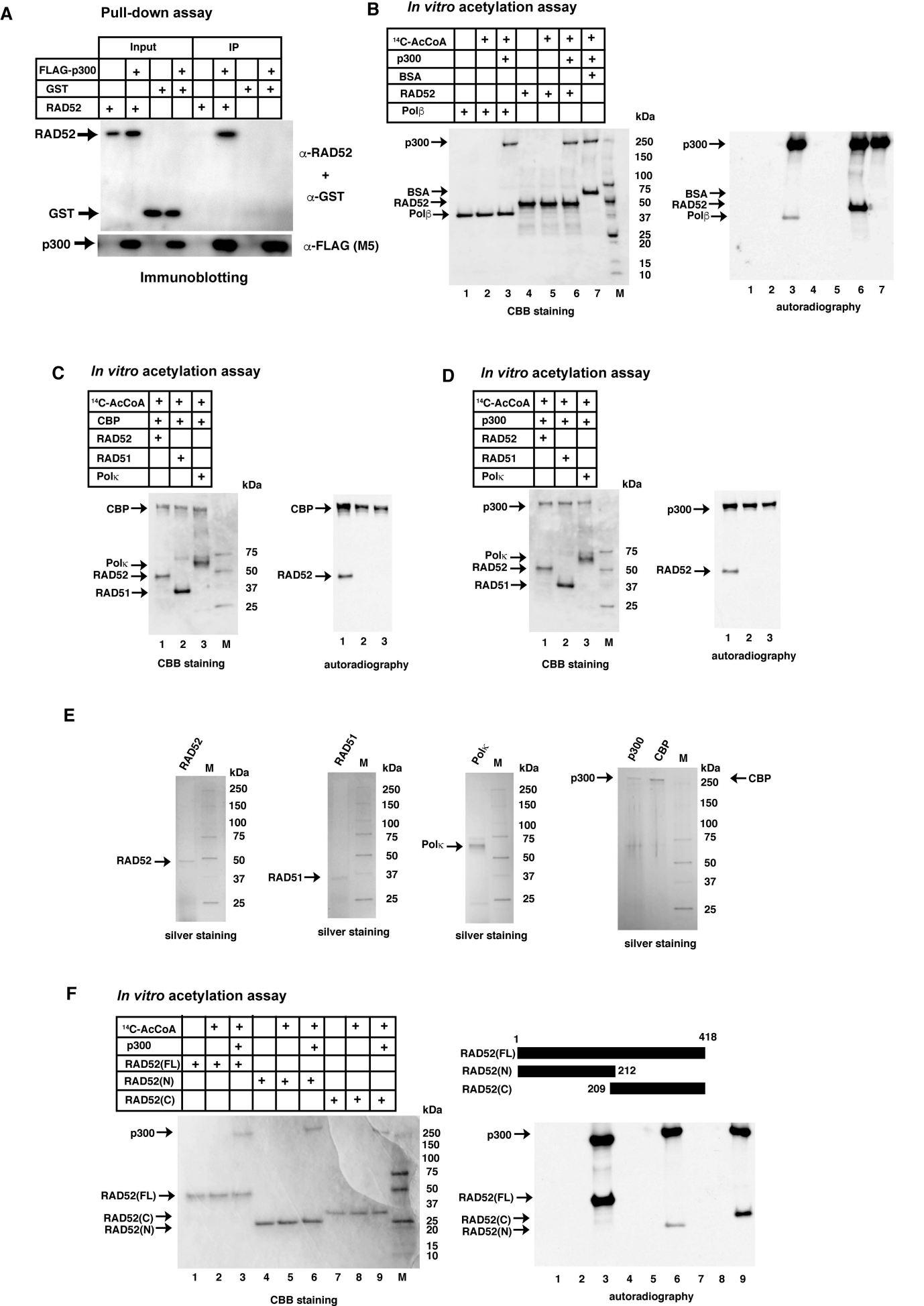

Supplement: S1 Fig — In vitro acetylation of human RAD52 by p300/CBP. (A) Physical interaction of human RAD52 with p300. The RAD52 or GST protein was incubated with or without FLAG-p300 in buffer P, and a pull-down assay was performed as described in the Supporting Materials and Methods. Input or immunoprecipitated (IP) proteins were detected by a mixture of anti-RAD52 and anti-GST antibodies (top) or an anti-FLAG (M5) antibody (bottom). (B, C, D, F) In vitro acetylation assays were performed as described in the Supporting Materials and Methods, using HAT buffer A containing sodium butyrate. Where indicated, [14C]Ac-CoA was added. The reactions were analyzed by Coomassie Brilliant Blue staining (left) or autoradiography (right). (B) RAD52 (3 μg), DNA polymerase β (3 μg), or BSA (3 μg) was incubated with FLAG-p300 (2 μg) where indicated. (C, D) RAD52 (1.5 μg), RAD51 (1.5 μg), or DNA polymerase κ (1.5 μg) was incubated with 1 μg of CBP-FLAG (C) or FLAG-p300 (D). (E) Silver staining of the RAD52, RAD51, DNA polymerase κ, FLAG-p300 and CBP-FLAG proteins used in S1C and S1D Fig. (F) RAD52 (FL, 2 μg), RAD52 (N, 2 μg), or RAD52 (C, 2 μg) was incubated with FLAG-p300 (1 μg), as indicated. (PDF) [file pgen.1007277.s002.pdf]

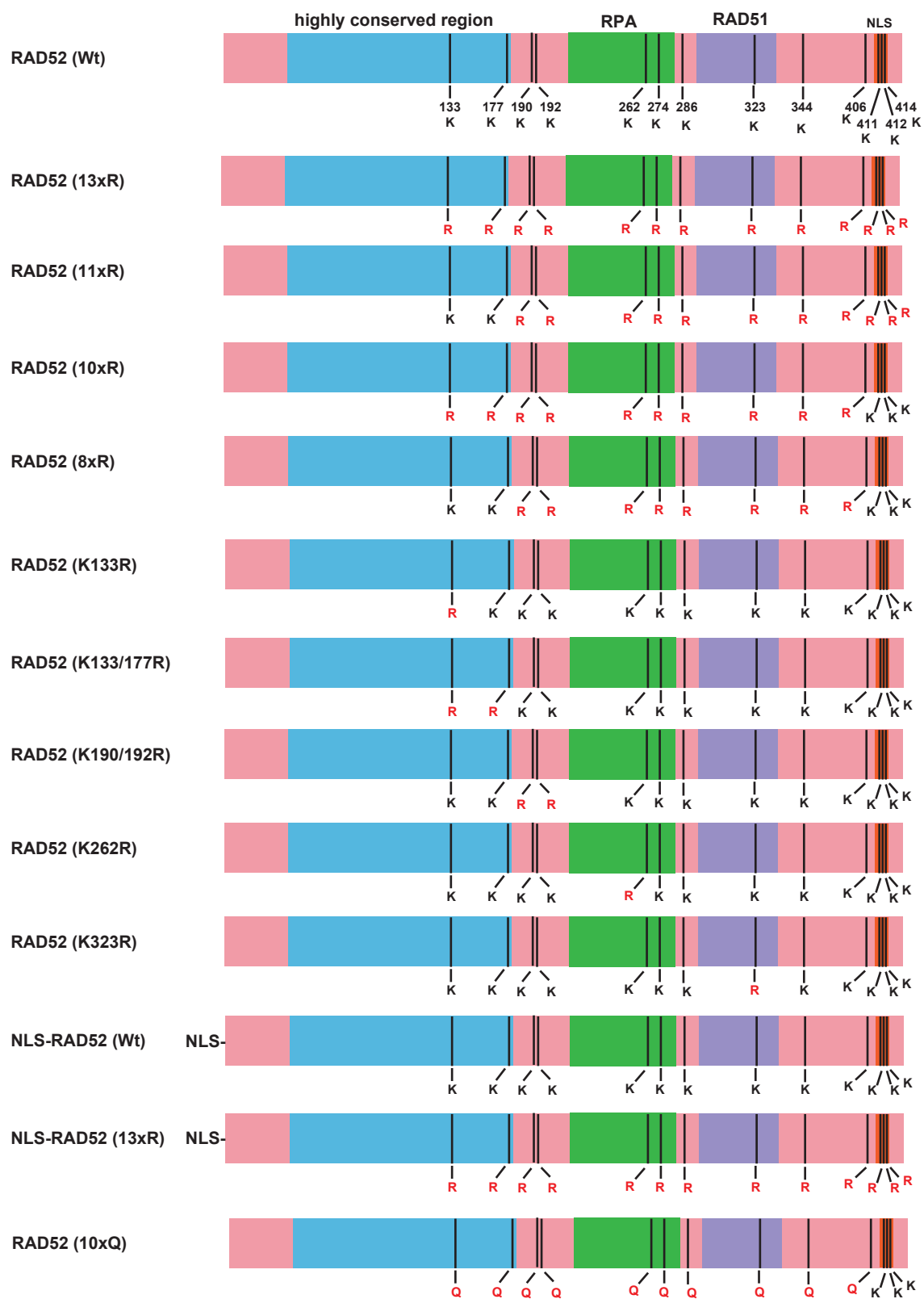

S3 Fig

Supplement: S3 Fig — Schematic representation of RAD52 wild-type and acetylation-site mutants used in this study. Mutations were introduced in functional domains, such as the highly conserved region (K133R, K133/K177R), the RPA binding region (K262R), and the RAD51 binding region (K323R), and also introduced outside the domains (190/192R). The 13xR and 11xR mutants contain multiple mutations including the NLS sequence, whereas the acetylation sites in the NLS sequence are normal in the 10xR and 8xR mutants. The NLS sequence is conjugated at the N-terminal in NLS-RAD52 (Wt) and NLS-RAD52 (13xR). The 10xQ mutant contains multiple glutamine (Q) substitutions at the same mutated sites as in the 10xR mutant. (PDF) [file pgen.1007277.s004.pdf]

**A**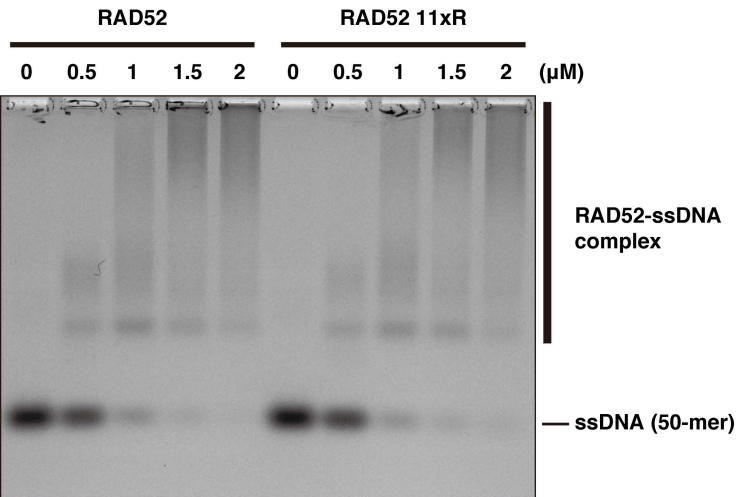**B**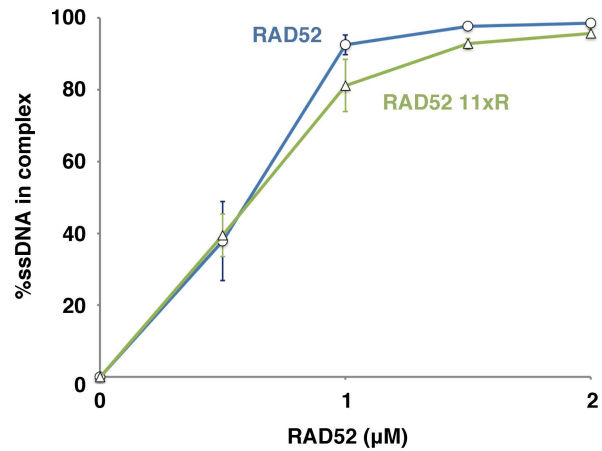**S4 Fig**

Supplement: S4 Fig — ssDNA binding activity of the RAD52 11xR mutant. (A) Electrophoretic mobility shift assay (EMSA) was performed using a 50-mer oligonucleotide (10 μM in nucleotides) with a Cy5 dye attached to the 5' end (oligo 1), and the indicated concentrations of RAD52 or the RAD52 11xR mutant. (B) Percentages of ssDNA bound by RAD52 (open circles, blue) and the RAD52 11xR mutant (open triangles, green) as a function of the protein concentration. (PDF) [file pgen.1007277.s005.pdf]

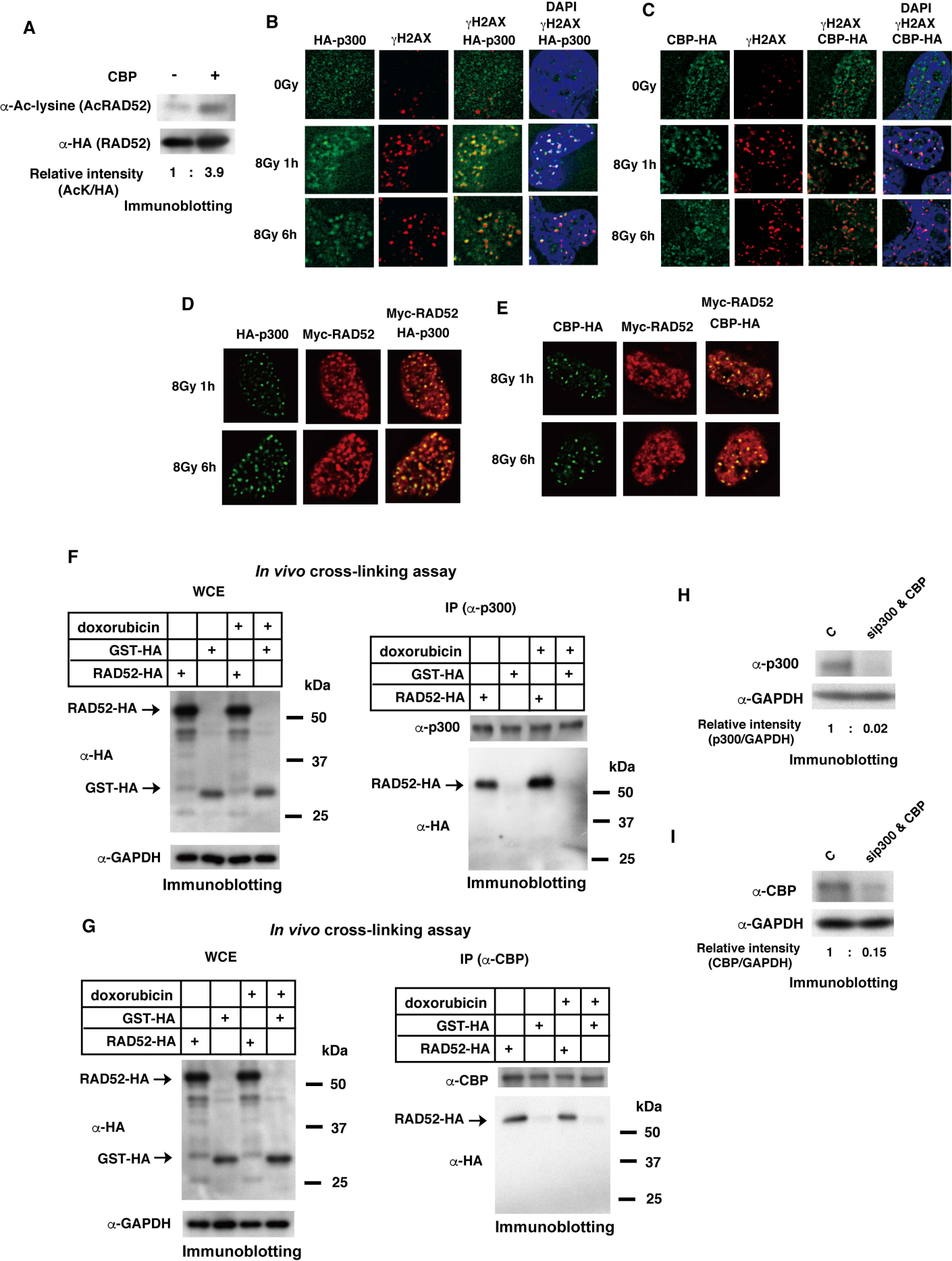

Supplement: S5 Fig — Human RAD52 is acetylated by p300/CBP in vivo. (A) Acetylation of the FLAG-RAD52-HA protein purified from T-Rex-293 (HEK293) cells was detected as described in the Supporting Materials and Methods, using the indicated antibodies. pRc/RSV-CBP-HA was transfected into cells, and FLAG-RAD52-HA was purified from cell extracts 24 h after transfection. (B, C) MRC5V1 cells were transfected with the expression plasmid for HA-p300 (B) or CBP-HA (C), and were unirradiated or irradiated with γ-rays (8 Gy) at 24 h after the transfection. At the indicated time after irradiation, the cells were subjected to immunofluorescent staining with an anti-HA (green) antibody, an anti-γH2AX (red) antibody, and 4',6-diamidino-2-phenylindole (DAPI, blue). (D, E) T-Rex-293 cells expressing Myc-RAD52 were transfected with the expression plasmid for HA-p300 (D) or CBP-HA (E), and were irradiated with γ-rays (8 Gy) at 24 h after the transfection. At the indicated time after irradiation, the cells were subjected to immunofluorescent staining with anti-HA (green) and anti-Myc (red) antibodies. (F, G) DSP-mediated cross-linking experiments were performed in the presence or absence of doxorubicin, as described in the Supporting Materials and Methods. RAD52-HA or GST-HA was expressed, as indicated. Whole cell extracts (WCE; left panel) or immunoprecipitates (IP; right panel) with anti-p300 (F) or anti-CBP (G) antibodies. (H, I) T-Rex-293 cells expressing FLAG-RAD52-HA were transfected with either a negative control siRNA or mixture of p300 and CBP-specific siRNAs. Cell extracts were subjected to immunoblotting analyses with the indicated antibodies. (A, H, I) The relative band intensities normalized to those of the HA or GAPDH bands are shown below the immunoblots. (PDF) [file pgen.1007277.s006.pdf]

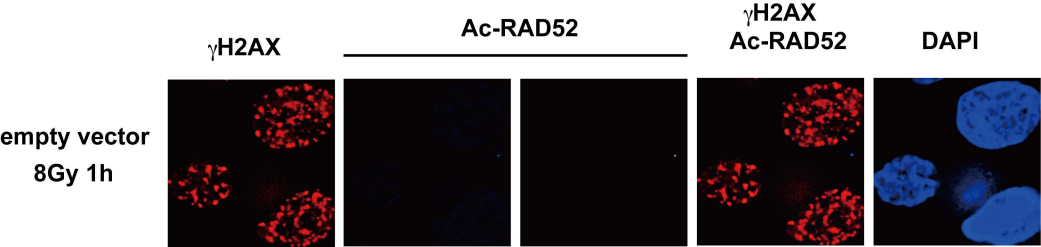

**S6 Fig**

Supplement: S6 Fig — Immunostaining of empty expression vector containing cells with acetylated RAD52 antibody. T-Rex-293 cells containing the empty pT-Rex-DEST30 vector were irradiated with γ-rays (8 Gy). At 1 hour after irradiation, the cells were subjected to immunofluorescent staining with an anti-γH2AX antibody (red), an anti-acetylated RAD52 at K323 antibody (blue or white), and DAPI (blue). (PDF) [file pgen.1007277.s007.pdf]

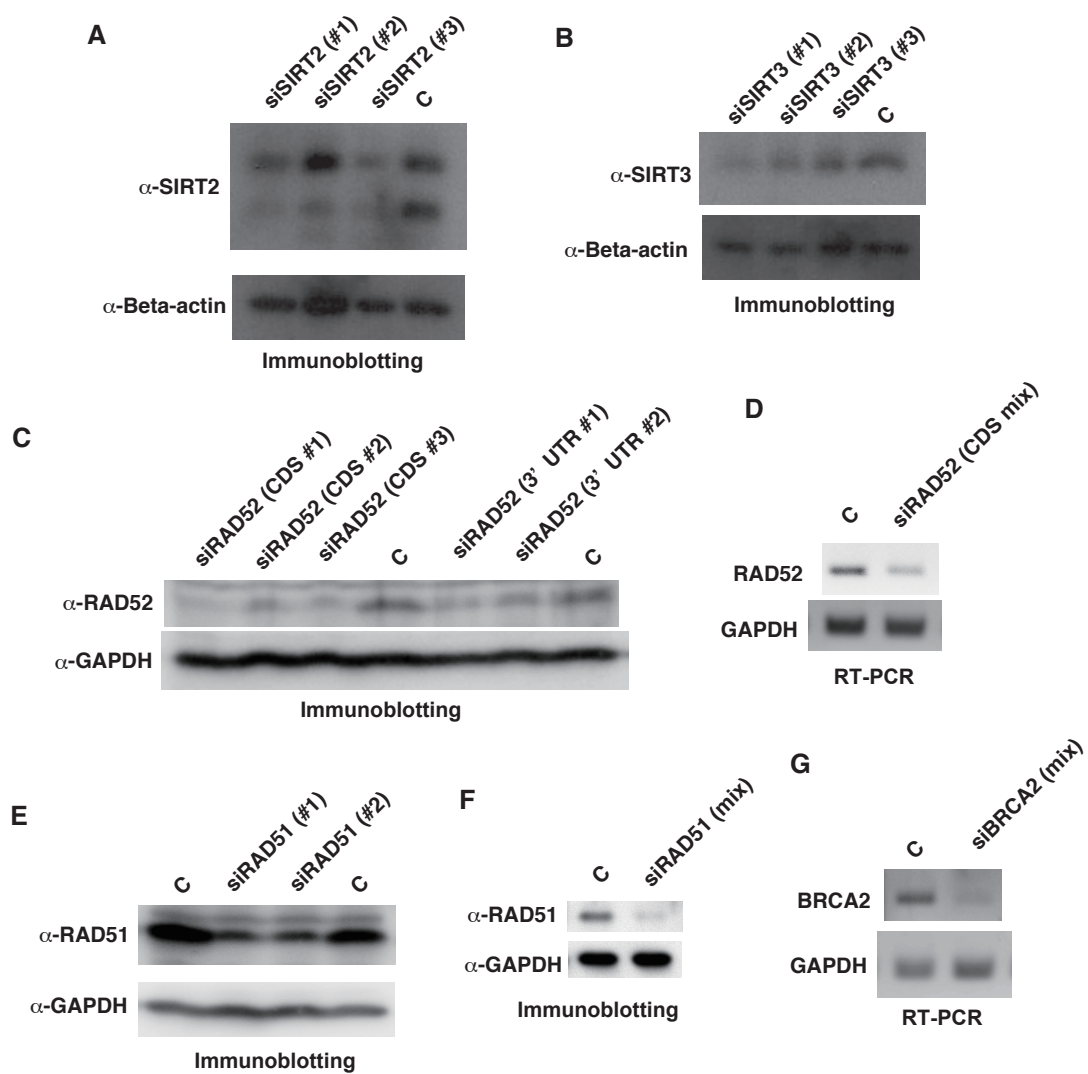

**S7 Fig**

Supplement: S7 Fig — Confirmation of siRNA-mediated knockdown. T-Rex-293 (A, B), HEK293 (C, E) or HeLa pDR-GFP (D, F, G) cells were transfected with the negative control (shown by “C”) or the indicated siRNA, as described in the Supporting Materials and Methods. The mixture of siRAD52 (CDS#1, CDS #2 and CDS #3), the mixture of siRAD51 (#1 and #2), and the mixture of siBRCA2 (#1, #2 and #3) were used in panels D, F, and G, respectively. Whole cell extracts (A, B, C, E and F) or total RNA from the cells (D and G) were prepared 72 h after siRNA transfection, and were subjected to immunoblotting (A, B, C, E and F) and RT-PCR (D and G) analyses, respectively. (PDF) [file pgen.1007277.s008.pdf]

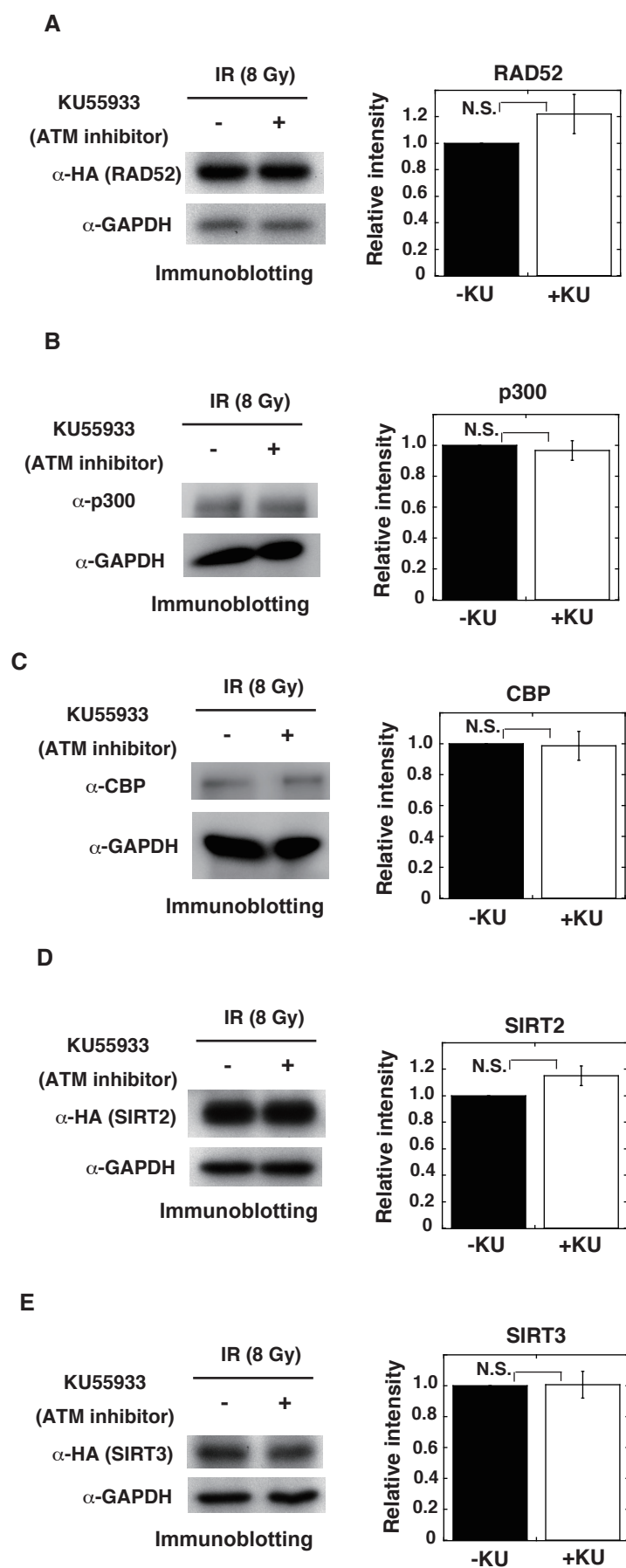

Supplement: S8 Fig — ATM inhibition does not affect cellular protein levels of RAD52, p300, CBP, SIRT2 and SIRT3. (A-E) Cell extracts prepared under the same experimental conditions as in Figs 11E, 12A, 12B, 13A and 13B were subjected to immunoblotting analyses, using the indicated antibodies. The relative band intensities normalized to those of the GAPDH bands are shown in the graphs. The graphs show the mean values and standard errors of the mean from 3–7 independent experiments (N.S., not significant by t-test). (PDF) [file pgen.1007277.s009.pdf]

**A**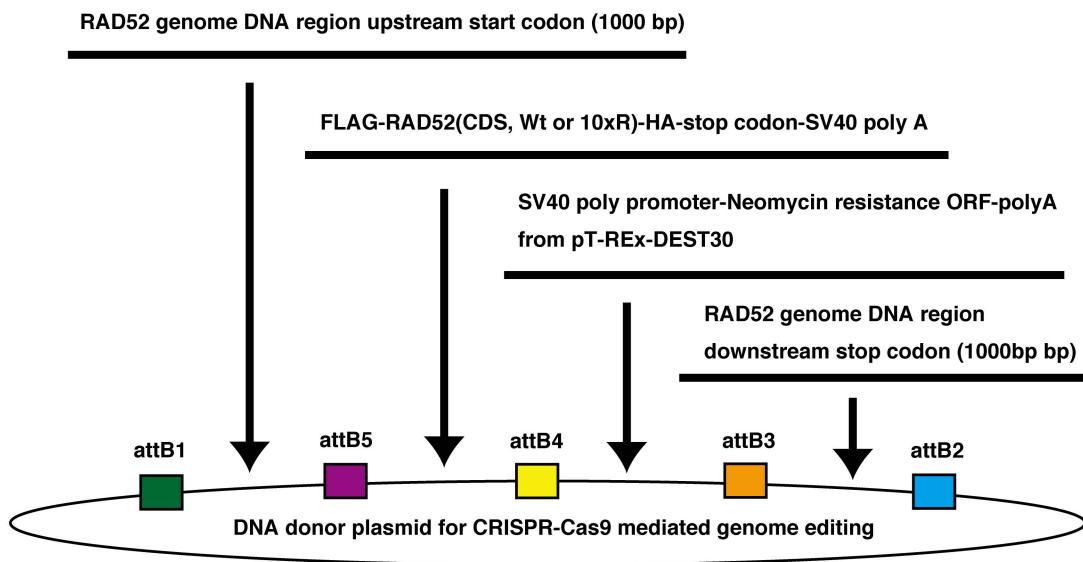**B**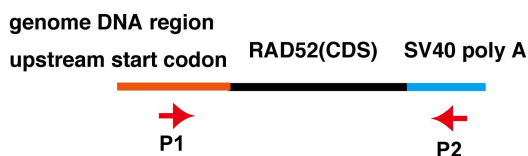**C**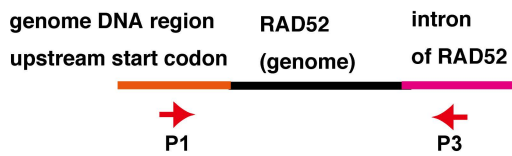**D**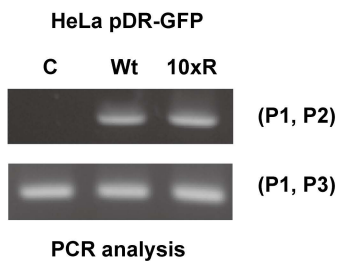**E**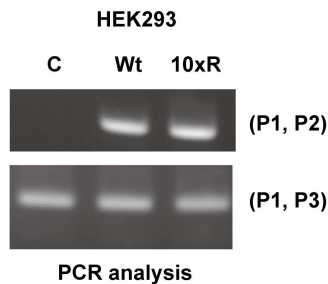**F**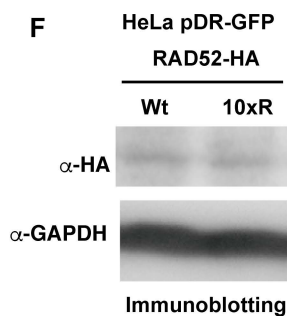**G**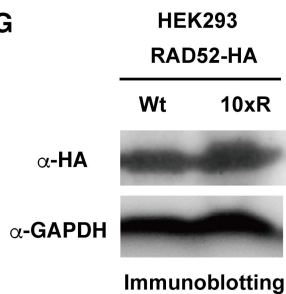

Supplement: S9 Fig — CRISPR-Cas9-mediated genome editing. (A) The donor plasmid DNA used for the CRISPR-Cas9-mediated knock-in of FLAG and the HA-tagged RAD52 coding sequence (CDS) into the genomic region of the targeted RAD52 gene. Multisite Gateway technology was used for the construction of the donor plasmid DNA, as described in the Supporting Materials and Methods. (B, C, D, E) PCR analysis of knock-in HeLa pDR-GFP and HEK293 cells. (B, C) The DNA sequences of the forward (P1) and reverse (P2 and P3) primers used in the PCR analysis are described in the Supporting Materials and Methods. The primers P1, P2 and P3 anneal the genomic DNA region upstream from the start codon of the RAD52 gene, the SV40 polyA region from pT-Rex-DEST30, and the intron region of RAD52, respectively. (D, E) Agarose gel electrophoresis of the PCR products. The genomic DNAs purified from HeLa pDR-GFP (D) and HEK293 (E) knock-in cells were analyzed by PCR with the indicated primers. As controls, genomic DNAs from untargeted cells were used in lane C. (F, G) Whole cell extracts of the HeLa pDR-GFP (F) and HEK293 (G) knock-in cells were subjected to immunoblotting analyses with the indicated antibodies. (PDF) [file pgen.1007277.s010.pdf]

**A**

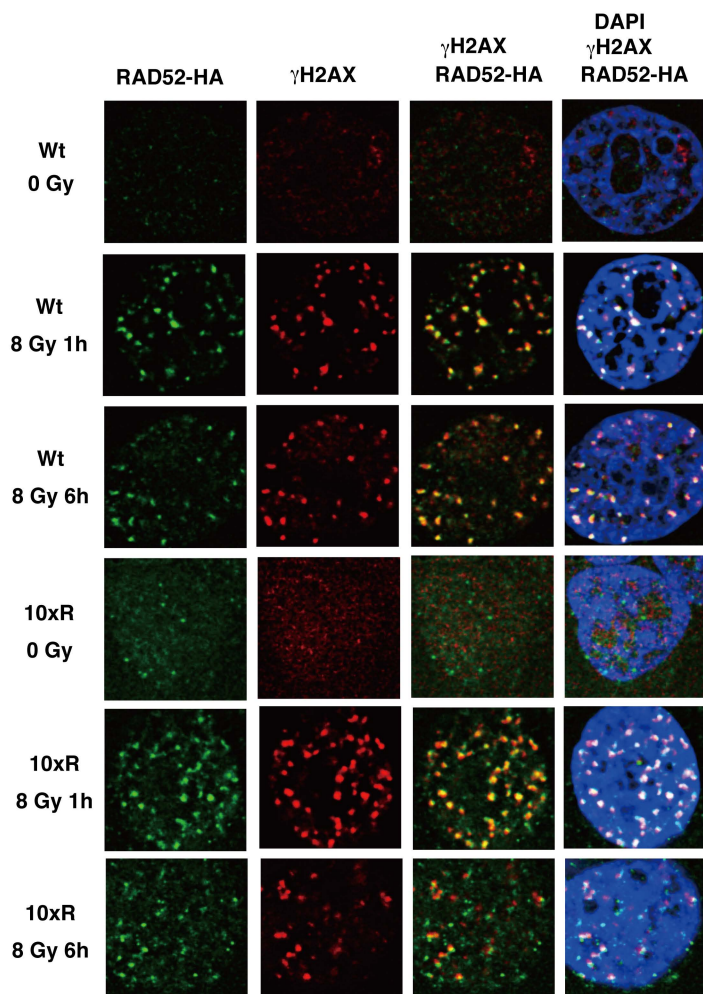

**B**

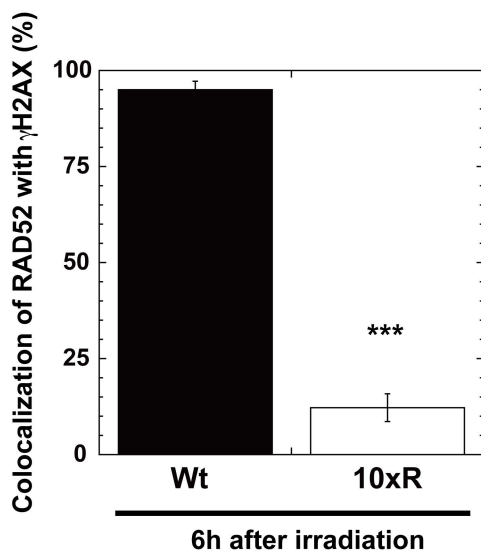

Supplement: S10 Fig — Effect of acetylation-deficient mutations on ionizing radiation-induced foci formation of RAD52 expressed by the native promoter. (A) RAD52 (Wt or 10xR) knock-in HeLa pDR-GFP cells, as shown in S9 Fig, were used. The cells expressing HA-tagged RAD52 proteins by the native promoter were treated with siRAD52 (3'UTR #1) in order to deplete the untagged endogenous RAD52. Two days after the siRNA treatment, the cells were unirradiated or irradiated with γ-rays (8 Gy). At the indicated time after irradiation, the cells were subjected to immunofluorescent staining with an anti-HA (green) antibody, an anti-γH2AX (red) antibody, and DAPI (blue). (B) The percentages of RAD52 foci colocalized with γH2AX at 6h after irradiation were calculated, as described in the Supporting Materials and Methods. Error bars indicate the standard error of the mean. Asterisks indicate statistically significant difference between Wt and 10xR (***, p<0.001 by t-test). (PDF) [file pgen.1007277.s011.pdf]

GFP-positive fraction

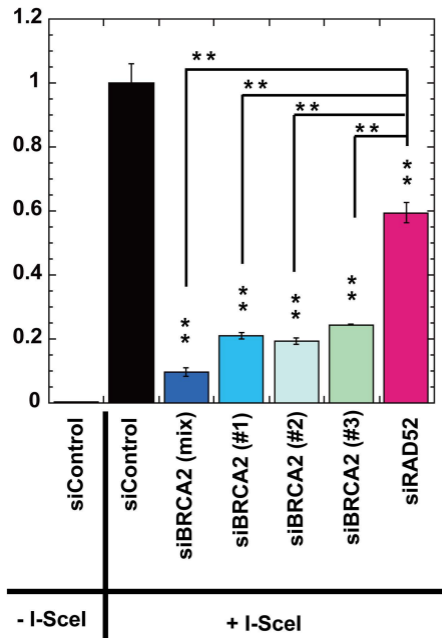

Supplement: S11 Fig — Comparison of knockdown effects on HR repair between RAD52 and BRCA2. HeLa pDR-GFP cells were transfected with the control or indicated siRNA, and were subjected to the DR-GFP assay as described in the Supporting Materials and Methods. In the column labeled siBRCA2 (mix), the mixture of siBRCA2 (#1, #2 and #3) was used. For RAD52 depletion, siRAD52 (CDS #1) was used. Error bars indicate the standard error of the mean from three samples. Asterisks indicate statistically significant differences of each protein-depleted sample, as compared with the control (+I-SceI) (**, P<0.01 by t-test). The samples connected by lines were also compared. (PDF) [file pgen.1007277.s012.pdf]

**A**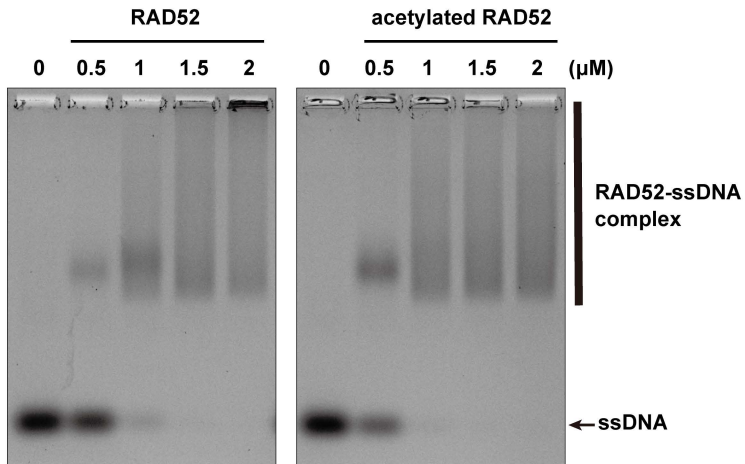**B**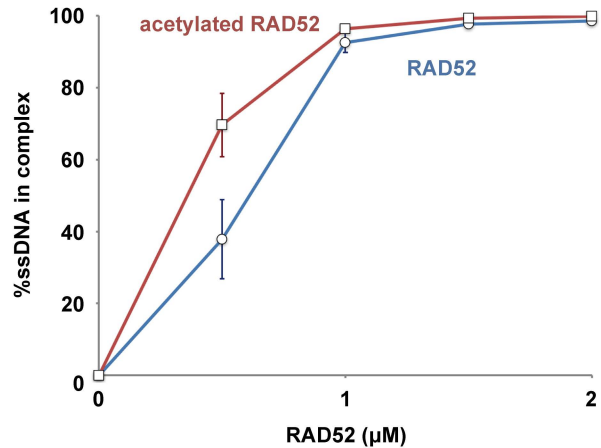**S13 Fig**

Supplement: S13 Fig — (A) EMSA was performed using a 50-mer oligonucleotide (10 μM in nucleotides) with a Cy5 dye attached to the 5' end (oligo 1), and the indicated concentrations of RAD52 or acetylated RAD52. (B) Quantification of (A). Percentage of ssDNA bound by RAD52 (open circles, blue) and acetylated RAD52 (open squares, red) as a function of protein concentration. Error bars indicate standard deviation (n = 3). (PDF) [file pgen.1007277.s014.pdf]
