# Supplementary material for: Novel function of HATs and HDACs in homologous recombination through acetylation of human RAD52 at double-strand break sites
Source: PLoS Genet. 2018 Mar 28;14(3):e1007277. doi: 10.1371/journal.pgen.1007277 (PMC5891081; doi:10.1371/journal.pgen.1007277)
Supplement: S2 Fig — Alignment of RAD52 proteins from Mus musculus (NCBI accession number AAA85794), Rattus norvegicus (NCBI accession number NP_001100087), Cricetulus griseus (NCBI accession number NP_001233693), Homo sapiens (NCBI accession number AAA85793), Pan troglodytes (NCBI accession number JAA24777), Rhesus monkey (NCBI accession number AFH33435), Gallus gallus (NCBI accession number NP_001161231), and Xenopus laevis (NCBI accession number NP_001089585), was performed using the Clustal 2.1 multiple sequence alignment program. (PDF) [file pgen.1007277.s003.pdf]

```
M. musculs      121 YHEDVGYGVSEGLRSKALSLEKARKEAVTDGLKRALRSFGNALGNCILDKDYLRSLNKLPRQLPLDVDLTKTKRKDFEPSVEQARYNSCR 210
R. norvegicus   121 YHEDVGYGVSEGLRSKALSLEKARKEAVTDGLKRALRSFGNALGNCILDKDYLKSLNRLPRQLPLEVDLTTTKREDFEPSVEQARYNSCR 210
C. griseus      121 YHEDVGYGVSEGLRSKALSLEKARKEAVTDGLKRALRSFGNALGNCILDKDYLRSLNKLPRQLPLEVDLTKAKREDFEPSVEQARYNSCL 210
H. sapiens      120 YHEDVGYGVSEGLKSKALSLEKARKEAVTDGLKRALRSFGNALGNCILDKDYLRSLNKLPRQLPLEVDLTKAKRQDLEPSVEEARYNSCR 209
P. troglodytes  120 YHEDVGYGVSEGLKSKALSLEKARKEAVTDGLKRALRSFGNALGNCILDKDYLRSLNKLPCQLPLEVDLTKAKRQDFEPSVEEARYNSCR 209
R. monkey       120 YHEDVGYGVSEGLKSKALSLEKARKEAVTDGLKRALRSFGNALGNCVLDKDYLRSLNKLPRQLPLEVDLTEAKRQDFEPSVEEARYNSCR 209
G. gallus       121 YHEDVGYGVSEGLKSKALSLEKARKEAVTDGLKRALKCFGNALGNCILDKDYLQAVNKLPRQMPPELDLVKTKRQDYEPEIEKARYDGCL 210
X. laevis       117 YHEDVGYGVSEGLKSKALSLEKARKEAVTDGLKRALKCFGNSLGNCILDKDYLRAVNKLPKQVPVELDLAQTKRQDFDASVERARYSSFQ 206

M. musculs      211 QNEALGLPKPQE--------VTSPCRSSPPHDSNIKLQGAKDISSS-CSLAATLESDATHQRKLRKLRQKQLQQQFR--EQMETRRQSHA 289
R. norvegicus   211 QDKASGLPKPQE--------AASPCRPSHPHDSNIKLQGAKDSSSSSCSLATTSESDATHQRKLRKLRQKQLQQQFRGTDGGETLSQTNT 292
C. griseus      211 QNEAPGPPKPQE--------AASPCRPSHPHDSNIRLQGAKDCSSS-CSLAAPMESDAIHQRKLRKLRQKQLQQQFR--QQMEAHLQGHT 289
H. sapiens      210 PNMALGHPQLQQ--------VTSPSRPSH-----AVIPADQDCSSR-SLSSSAVESEATHQRKLR---QKQLQQQFR--ERMEK--QQVR 278
P. troglodytes  210 PNMALGHPQLQE--------ATSPSRPSH-----AVIQADKDCSSR-SLTSSAVESEATHQRKLR---QKQLQQQFR--ERMEK--QHVR 278
R. monkey       210 PNMALGHPQLQE--------VTSPSRPSR-----VVIQGDKDCSSR-GLTSSTVESEATYQRKLR---QKQLQQQFR--ERMEK--QHVH 278
G. gallus       211 ERQNPGWRQQCE---------TAPTCKPTHTEASGVTEDQKQPSSSGNTDSPAVECDATYQRKLR---QKQLQQQFW--EQMEKRRQVKE 286
X. laevis       207 QAQKQAERPQPEPVVQLPLRSSDPSLSEHNHEGSTRISPEDNCTSTGSAMSYAPDMDATYQRKLR---QKQLQQQFR--EQMERKQQTRE 291

M. musculs      290 PA---EEVAAKH-AVLPAPP-KHSTPVTAASELLQEKVVFP-------DNLEENLEMWDLTPDLED-----IIKPLCRAEPAQTSATRTF 362
R. norvegicus   293 SQ---RHPPPPHSAALPAPP-KHSTPVPAASACLREKAVLP-------DNLEENLEMWDLTPDLED-----IIKPLCRTEPPQTSVTRTL 366
C. griseus      290 PA---VKVKAEREAVLPDLPPKHSTPVTAASELLREKAIFP-------DNPEDNLEMWDLTPDLED-----IIKPLSRPEPPQTSATRVQ 364
H. sapiens      279 VS---TPSAEKSEAAPPAPPVTHSTPVT-VSEPLLEKDFLAGVTQELIKTLEDNSEKWAVTPDAGDG----VVKPSSRADPAQTSDTLAL 360
P. troglodytes  279 VS---TPSAEKSE-APPAPPLTHSTPVT-VSEPLPEKDFLAGVTQELIKTLEDNSEKWAVAPDAGDG----VVRPSSTADPAQASDTLAL 359
R. monkey       279 VS---RPSAEKSEAARAAPPLTHSTPVTAVSEPLPEKDFLAGVTQELIKTLEDNSEKWAVTPDAGDG----VAKPSSRADPAQAADTLAL 361
G. gallus       287 ---VTPSSKQATAN----PPVKHSTPAAVQQ-ELAIEEEFF----------ADDLELWDISLETTDLNKLMCHKAAGSPAAQQPPETPHR 358
X. laevis       292 PETLPDTSREGTAYPARVPPLGHSTPAAAAQPQVAPEEEFL----------ADDPELWDIPLDAVEMDPFSNGRIQPVPSAVSTPVAPHG 371

M. musculs      363 NN----QDSVPHIHCHQKPQ---EKPGPGHLQTCNTNQHVLGSREDSEPHRKSQ--DLKKRKLDPS 419
R. norvegicus   367 NNQAVIQDSVPHIHCHQKVQ---EKPGPGHLQTCSTNQHVLGSRENSESHRKSQ--DLKKRKLDPS 427
C. griseus      365 VI----QDGVLHGLCHQMPP---EKHEAGHLQAHSTHQHVLG---NSDSHRKSQ--DLKKRKGDPS 418
H. sapiens      361 NNQMVTQNRTPHSVCHQKPQ---AKSGSWDLQTYSADQRTTG---NWESHRKSQ--DMKKRKYDPS 418
P. troglodytes  360 NNQMVTQNRTPHSLCHQKPQ---AKSGSWDLQTYSADQRTTG---NWESHRKSQ--DMKKRKYDPS 417
R. monkey       362 NHQMVTQDRTPHSLCHQKPQ---AKSGSWDLQTYSTDQRVTG---NWESHRKSQ--DMKKRKYDPS 419
G. gallus       359 RHQMTTRNRTPQRMHYHKPPVRFAQLQPSAALTSNSHGANQRTPAEHSPYRRSQ--SWKKRRLEPT 422
X. laevis       372 QHQMLTRSKTPQRQNHQR--------QPLRPTSWNQPNGNPAPRLDRSPYQQHQGLLMKKRRLEPS 429
```

S2 Fig
