# Supplementary material for: Novel function of HATs and HDACs in homologous recombination through acetylation of human RAD52 at double-strand break sites
Source: PLoS Genet. 2018 Mar 28;14(3):e1007277. doi: 10.1371/journal.pgen.1007277 (PMC5891081; doi:10.1371/journal.pgen.1007277)
Supplement: S12 Fig — Interactions of human RAD52 with human RAD52, RAD51 and RPA subunits by a yeast two-hybrid analysis. Yeast MAV203 cells were transformed with expression vectors encoding the indicated NLS-GAL4-activation domain (AD)- and GAL4-DNA binding domain (DBD)-fused proteins. As controls, the empty vector for the expression of the GAL4-DBD fusion protein was used (1–5). The negative control (C1) and positive controls (C2 and C3) were also included. Each transformant was examined for growth on an SC-Leu-Trp agar plate and an SC-Leu-Trp-His agar plate containing 25 mM 3-Amino-1,2,4-Triazole (3AT). (PDF) [file pgen.1007277.s013.pdf]

Yeast two-hybrid analysis

SC-Leu-Trp

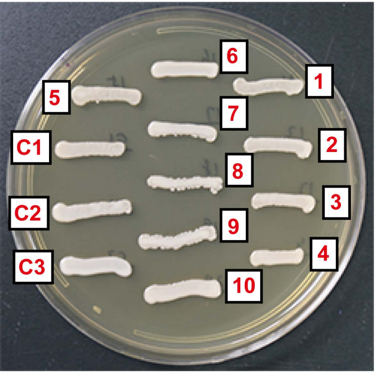

SC-Leu-Trp-His + 25 mM 3AT

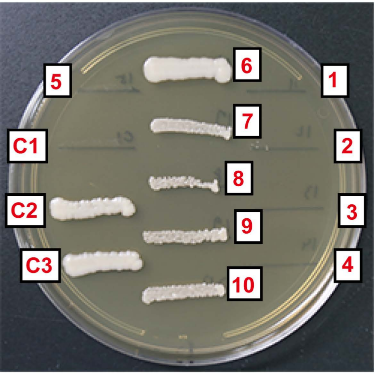

	NLS-GAL4 AD	GAL4 DBD	
1	RAD52 (Wt)	-	
2	RAD51	-	
3	RPA1	-	
4	RPA2	-	
5	RPA3	-	
6	RAD52 (Wt)	RAD52 (Wt)	
7	RAD51	RAD52 (Wt)	
8	RPA1	RAD52 (Wt)	
9	RPA2	RAD52 (Wt)	
10	RPA3	RAD52 (Wt)	
C1	RalGDS-m2	Krev1	negative control
C2	RalGDS-m1	Krev1	positive control
C3	RalGDS-wt	Krev1	positive control

S12 Fig
