# Supplementary material for: Novel function of HATs and HDACs in homologous recombination through acetylation of human RAD52 at double-strand break sites
Source: PLoS Genet. 2018 Mar 28;14(3):e1007277. doi: 10.1371/journal.pgen.1007277 (PMC5891081; doi:10.1371/journal.pgen.1007277)
Supplement: S1 Table — (PDF) [file pgen.1007277.s015.pdf]

S1 Table. Mascot search results of tryptic-peptide fragment of acetylated RAD52 (FL)

Matched peptides shown in red

```
  1 MSGTEEAILG GRDSHPAAGG GSVLCFGQCQ YTAEEYQAIQ KALRQRLGPE
 51 YISSRMAGGG QKVCYIEGHR VINLANEMFG YNGWAHSITQ QNVDFVDLNN
101 GKFYVGVCAF VRVQLKDGSY HEDVGYGVSE GLKSKALSLE KARKEAVTDG
151 LKRALRSFGN ALGNCILDKD YLRSLNKLPR QLPLEVDLTK AKRQDLEPSV
201 EEARYNSCRP NMALGHPQLQ QVTSPSRPSH AVIPADQDCS SRSLSSSAVE
251 SEATHQRKLR QKQLQQFRE RMEKQQVRVS TPSAEKSEAA PPAPPVTHST
301 PVTVSEPLLE KDFLAGVTQE LIKTLEDNSE KWAVTPDAGD GVVKPSSRAD
351 PAQTSDTLAL NNQMVTQNRT PHSVCHQKPQ AKSGSWDLQT YSADQRTTGN
401 WESHRKSQDM KKRKYDPS
```

Cleavage by Trypsin: cuts C-terminal side of KR unless next residue is P

Sequence Coverage: 73%

a: Residue number of first and last amino acid of peptide fragment

b: Observed m/z value of precursor

c: Experimental molecular weight of precursor

d: Theoretical molecular weight of precursor

e: Difference between Mr (exp) and Mr (calc)

f: Number of missed cleavage

g: Cleavage site (.) in peptide sequence

Acetyl site	Start-End[a]	Observed[b]	Mr (exp)[c]	Mr (calc)[d]	Delta[e]	Miss[f]	Ion score	Sequence (variable modifications)[g]
K190	181 - 192	698.6200	1395.2254	1395.7973	-0.5719	1	26	R.QLPLEVDLTKAK.R (Acetyl (K))
K190	181 - 192	698.6200	1397.5054	1395.7973	1.7081	1	50	R.QLPLEVDLTKAK.R (Acetyl (K))
K190, 192	181 - 193	798.6600	1595.3054	1593.9090	1.3965	2	47	R.QLPLEVDLTKAKR.Q (2Acetyl (K))
K192	191 - 204	835.6500	1669.2854	1668.8431	0.4424	2	62	K.AKRQDLEPSVEEAR.Y (Acetyl (K))
K262	261 - 269	624.2600	1246.5054	1244.6626	1.8429	1	47	R.QKQLQQQFR.E (Acetyl (K))
K284	279 - 311	1133.1900	3396.5482	3394.7508	1.7974	1	62	R.VSTPSAEKSEAAPPAPPVTHSTPVTVSEPLLEK.D (Acetyl (K))
K344	332 - 348	892.6400	1783.2654	1782.8901	0.3754	0	54	K.WAVTPDAGDGVVKPSSR.A (Acetyl (K))
K406, 411, 412	406 - 413	574.1900	1146.3654	1145.5862	0.7792	3	60	R.KSQDMKKR.K (3Acetyl (K))
K406, 411, 412	406 - 413	582.2700	1162.5254	1161.5812	0.9443	3	39	R.KSQDMKKR.K (Oxidation (M); 3Acetyl (K))

Results of the Identified acetylated peptide fragments are shown.
