# Supplementary material for: Novel function of HATs and HDACs in homologous recombination through acetylation of human RAD52 at double-strand break sites
Source: PLoS Genet. 2018 Mar 28;14(3):e1007277. doi: 10.1371/journal.pgen.1007277 (PMC5891081; doi:10.1371/journal.pgen.1007277)
Supplement: S2 Table — (PDF) [file pgen.1007277.s016.pdf]

S2 Table. Mascot search results of Asp-N peptide fragment of acetylated RAD52 (FL)

Matched peptides shown in red

```
  1 MSGTEEAILG GRDSHPAAGG GSVLCFGQCQ YTAEEYQAIQ KALRQRLGPE
 51 YISSRMAGGG QKVCYIEGHR VINLANEMFG YNGWAHSITQ QNVDFVDLNN
101 GKFYVGVCAF VRVQLKDGSY HEDVGYGVSE GLKSKALSLE KARKEAVTDG
151 LKRALRSFGN ALGNCILDKD YLRSLNKLPR QLPLEVDLTK AKRQDLEPSV
201 EEARYNSCRP NMALGHPQLQ QVTSPSRPSH AVIPADQDCS SRSLSSSAVE
251 SEATHQRKLR QKQLQQFRE RMEKQQVRVS TPSAEKSEAA PPAPPVTHST
301 PVTVSEPLLE KDFLAGVTQE LIKTLEDNSE KWAVTPDAGD GVVKPSSRAD
351 PAQTSDTLAL NNQMVTQNRT PHSVCHQKPQ AKSGSWDLQT YSADQRTTGN
401 WESHRKSQDM KKRKYDPS
```

Cleavage by Asp-N: cuts N-terminal side of DE

Sequence Coverage: 40%

Acetyl site	Start-End	Observed	Mr (exp)	Mr (calc)	Delta	Miss	Ion score	Sequence (variable modifications)
K190, 192	187 - 194	522.7700	1043.5254	1042.5771	0.9484	0	43	V.DLTKAKRQ.D (2Acetyl (K))
K274	273 - 284	1371.7100	1370.7027	1370.7154	-0.0127	0	25	M.EKQQVRVSTPSA.E (Acetyl (K))
K274	273 - 284	686.7900	1371.5654	1370.7154	0.8501	0	37	M.EKQQVRVSTPSA.E (Acetyl (K))
K286	273 - 287	858.7800	1715.5454	1714.8849	0.6605	1	32	M.EKQQVRVSTPSAEKS.E (Acetyl (K))
K274, 286	273 - 287	879.8200	1757.6254	1756.8955	0.7299	1	46	M.EKQQVRVSTPSAEKS.E (2Acetyl (K))
K286	285 - 305	1073.1500	2144.2854	2143.0797	1.2057	1	34	A.EKSEAAPPAPPVTHSTPVTVS.E (Acetyl (K))
K323	320 - 326	887.6200	886.6127	886.5011	0.1116	1	24	Q.ELIKTLE.D (Acetyl (K))
K344	340 - 349	529.7300	1057.4454	1056.5564	0.8891	0	25	G.DGVVKPSSRA.D (Acetyl (K))
K411, 412, 414	409 - 415	556.2400	1110.4654	1109.5539	0.9116	0	34	Q.DMKKRKY.D (3Acetyl (K); Oxidation (M))

Results of the Identified acetylated peptide fragments are shown.
