# Supplementary material for: Novel function of HATs and HDACs in homologous recombination through acetylation of human RAD52 at double-strand break sites
Source: PLoS Genet. 2018 Mar 28;14(3):e1007277. doi: 10.1371/journal.pgen.1007277 (PMC5891081; doi:10.1371/journal.pgen.1007277)
Supplement: S3 Table — (PDF) [file pgen.1007277.s017.pdf]

S3 Table. Mascot search results of peptide fragment of acetylated RAD52 (N)

Cleavage by Trypsin: cuts C-terminal side of KR unless next residue is P

Acetyl site	Start-End	Observed	Mr (exp)	Mr (calc)	Delta	Miss	Ion score	Sequence
K190	181 - 192	1379.9700	1378.9627	1378.7708	0.1920	1	45	R.QLPLEVDLTKAK.R (Gln->pyro-Glu (N-term Q); Acetyl (K))
K190	181 - 192	690.9700	1379.9254	1378.7708	1.1547	1	56	R.QLPLEVDLTKAK.R (Gln->pyro-Glu (N-term Q); Acetyl (K))
K190	181 - 192	698.5300	1395.0454	1395.7973	-0.7519	1	32	R.QLPLEVDLTKAK.R (Acetyl (K))
K190	181 - 192	699.2600	1396.5054	1395.7973	0.7081	1	45	R.QLPLEVDLTKAK.R (Acetyl (K))
K190 or K192	181 - 193	769.0500	1536.0854	1534.8719	1.2136	2	45	R.QLPLEVDLTKAKR.Q (Gln->pyro-Glu (N-term Q); Acetyl (K))
K190, 192	181 - 193	1577.7500	1576.7427	1576.8824	-0.1397	2	35	R.QLPLEVDLTKAKR.Q (Gln->pyro-Glu (N-term Q); Acetyl (K))
K190, 192	181 - 193	789.9800	1577.9454	1576.8824	1.0630	2	64	R.QLPLEVDLTKAKR.Q (Gln->pyro-Glu (N-term Q); Acetyl (K))
K190, 192	181 - 193	798.1000	1594.1854	1593.9090	0.2765	2	39	R.QLPLEVDLTKAKR.Q (2Acetyl (K))
K190, 192	181 - 193	798.2000	1594.3854	1593.9090	0.4765	2	27	R.QLPLEVDLTKAKR.Q (2Acetyl (K))
K190, 192	181 - 193	798.2900	1594.5654	1593.9090	0.6565	2	41	R.QLPLEVDLTKAKR.Q (2Acetyl (K))
K190, 192	181 - 193	798.3000	1594.5854	1593.9090	0.6765	2	36	R.QLPLEVDLTKAKR.Q (2Acetyl (K))
K190, 192	181 - 193	798.3500	1594.6854	1593.9090	0.7765	2	46	R.QLPLEVDLTKAKR.Q (2Acetyl (K))
K190, 192	181 - 204	950.7900	2849.3482	2847.4977	1.8504	3	33	R.QLPLEVDLTKAKRQDLEPSVEEAR.Y (2Acetyl (K))
K192	191 - 204	835.7500	1669.4854	1668.8431	0.6424	2	69	K.AKRQDLEPSVEEAR.Y (Acetyl (K))
K192	191 - 204	557.7900	1670.3482	1668.8431	1.5051	2	36	K.AKRQDLEPSVEEAR.Y (Acetyl (K))

Cleavage by Asp-N: cuts N-terminal side of DE

Acetyl site	Start-End	Observed	Mr (exp)	Mr (calc)	Delta	Miss	Ion score	Sequence (variable modifications)
K133	123 - 139	883.6200	1765.2254	1763.9305	1.2949	1	37	E.DVGYGVSEGLKSKALSL.E (Acetyl (K))
K177	170 - 184	935.0900	1868.1654	1867.0679	1.0975	0	21	K.DYLRSLNKLPRQLPL.E (Acetyl (K))
K177	170 - 186	699.9500	2096.8282	2095.1789	1.6492	1	27	K.DYLRSLNKLPRQLPLEV.D (Acetyl (K))
K190, 192	187 - 194	522.1100	1042.2054	1042.5771	-0.3716	0	22	V.DLTKAKRQ.D (2Acetyl (K))
K190, 192	187 - 194	522.7700	1043.5254	1042.5771	0.9484	0	43	V.DLTKAKRQ.D (2Acetyl (K))

Results of the Identified acetylated peptide fragments are shown.
