# Supplementary material for: Novel function of HATs and HDACs in homologous recombination through acetylation of human RAD52 at double-strand break sites
Source: PLoS Genet. 2018 Mar 28;14(3):e1007277. doi: 10.1371/journal.pgen.1007277 (PMC5891081; doi:10.1371/journal.pgen.1007277)
Supplement: S4 Table — (PDF) [file pgen.1007277.s018.pdf]

S4 Table. Mascot search results of peptide fragment of acetylated RAD52 (C)

Cleavage by Trypsin: cuts C-terminal side of KR unless next residue is P

Acetyl site	Start-End	Observed	Mr (exp)	Mr (calc)	Delta	Miss	Ion score	Sequence (variable modifications)
K286	279 - 311	1132.9900	3395.9482	3394.7508	1.1974	1	55	R.VSTPSAEKSEAAPPAPPVTHSTPVTVSEPLLEK.D (Acetyl (K))

Cleavage by Asp-N: cuts N-terminal side of DE

Acetyl site	Start-End	Observed	Mr (exp)	Mr (calc)	Delta	Miss	Ion score	Sequence (variable modifications)
K274	273 - 284	686.9700	1371.9254	1395.7973	1.2101	0	35	M.EKQQVRVSTPSA.E (Acetyl (K))
K286	273 - 287	858.8200	1715.6254	1395.7973	0.7405	1	39	M.EKQQVRVSTPSAEKS.E (Acetyl (K))
K286	285 - 305	1072.8300	2143.0797	1593.9090	0.5657	1	52	A.EKSEAAPPAPPVTHSTPVTVS.E (Acetyl (K))

Results of the Identified acetylated peptide fragments are shown.
